# Divergent Evolutionary Patterns of NAC Transcription Factors Are Associated with Diversification and Gene Duplications in Angiosperm

**DOI:** 10.3389/fpls.2017.01156

**Published:** 2017-06-30

**Authors:** Xiaoli Jin, Jing Ren, Eviatar Nevo, Xuegui Yin, Dongfa Sun, Junhua Peng

**Affiliations:** ^1^Department of Agronomy and the Key Laboratory of Crop Germplasm Resource of Zhejiang Province, Zhejiang UniversityHangzhou, China; ^2^Shandong Provincial Key Laboratory of Biophysics, Institute of Biophysics, Dezhou UniversityDezhou, China; ^3^Department of Evolutionary and Environmental Biology, Institute of Evolution, University of HaifaHaifa, Israel; ^4^Department of Biotechnology, College of Agriculture, Guangdong Ocean UniversityZhanjiang, China; ^5^Department of Agronomy, College of Plant Science and Technology, Huazhong Agricultural UniversityWuhan, China; ^6^Life Science & Technology Center, and the State Key Lab of Crop Breeding Technology Innovation and Integration, China National Seed Group Co., Ltd.Wuhan, China

**Keywords:** NAC transcription factors, gene duplication, evolution rate, divergent selection, angiosperm

## Abstract

NAC (NAM/ATAF/CUC) proteins constitute one of the biggest plant-specific transcription factor (TF) families and have crucial roles in diverse developmental programs during plant growth. Phylogenetic analyses have revealed both conserved and lineage-specific NAC subfamilies, among which various origins and distinct features were observed. It is reasonable to hypothesize that there should be divergent evolutionary patterns of NAC TFs both between dicots and monocots, and among NAC subfamilies. In this study, we compared the gene duplication and loss, evolutionary rate, and selective pattern among non-lineage specific NAC subfamilies, as well as those between dicots and monocots, through genome-wide analyses of sequence and functional data in six dicot and five grass lineages. The number of genes gained in the dicot lineages was much larger than that in the grass lineages, while fewer gene losses were observed in the grass than that in the dicots. We revealed (1) uneven constitution of Clusters of Orthologous Groups (COGs) and contrasting birth/death rates among subfamilies, and (2) two distinct evolutionary scenarios of NAC TFs between dicots and grasses. Our results demonstrated that relaxed selection, resulting from concerted gene duplications, may have permitted substitutions responsible for functional divergence of NAC genes into new lineages. The underlying mechanism of distinct evolutionary fates of NAC TFs shed lights on how evolutionary divergence contributes to differences in establishing NAC gene subfamilies and thus impacts the distinct features between dicots and grasses.

## Introduction

Gene duplication plays significant roles in evolution of organisms. Polyploidization, also known as whole-genome duplication (WGD), is now recognized as an important evolutionary force in the origin and diversification of plants (De Bodt et al., 2005; Freeling and Thomas, 2006; Soltis et al., 2009; Sarilar et al., 2013). Most of the flowering plant lineages had experienced one or more rounds of ancient WGD through the evolutionary history (Cui et al., 2006; Jiao et al., 2011). WGD results in genetic redundancy, which is acted upon by selection, drift, and mutation, and represents with the emergence of new gene functions (Ohno, 1970; Zhang, 2003; Sarilar et al., 2013). Indeed, gene duplication is an important source of novelty, especially for gene family diversification. Paralogous genes derived from gene duplication have identical sequences and functions but tend to diverge in both regulating and coding regions. The genes retained in duplicates are not evenly distributed among different functional categories. For example, three evident ancient polyploidization events in the *Arabidopsis thaliana* genome account for over 90% of the duplications in transcription factors (TFs) and signal transducer gene families (Maere et al., 2005). Therefore, genes relating with transcription regulation, signal transduction and development are preferentially maintained following genome duplications (Seoighe and Gehring, 2004; De Bodt et al., 2005; Maere et al., 2005).

TFs and their corresponding *cis*-regulatory sequences act as molecular switches for gene expression, regulating their temporal and spatial expression. A large portion of the plant genome is dedicated to TFs, with up to 2,500 TFs being coded into 64 gene families in the *Arabidopsis* genome (Perez-Rodriguez et al., 2010). In plants, TFs often belong to large gene families, such as the NAC (NAM, ATAF1, 2, and CUC2) TF gene family (Zhao et al., 2016). The NAC acronym was derived from three genes that were initially discovered to contain highly conserved NAC domains: *NAM* (*no apical meristem*), *ATAF1* and *-2*, and *CUC2* (*cupshaped cotyledon*) (Souer et al., 1996; Aida et al., 1997; Zhao et al., 2016; http://www.ncbi.nlm.nih.gov). Compared to the NAC domains, the C-terminal regions of NAC TFs are rather variable (Ooka et al., 2003; Zhu et al., 2012; http://www.ncbi.nlm.nih.gov) and result in the diversity of the transcriptional activities showed by NAC proteins (Xie et al., 2000; Yamaguchi et al., 2008; Jensen et al., 2010; http://www.ncbi.nlm.nih.gov). The NAC proteins form one of the largest families of plant-specific TFs, with <100 members discovered in *Arabidopsis*, rice, and other major angiosperm lineages, which probably expanded via WGD and other gene duplication events (Zhu et al., 2012). NAC TFs are crucial for plant growth and are associated with diverse biological programs, including various developmental processes (Olsen et al., 2005), senescence (Uauy et al., 2006; Yang et al., 2011), secondary walls or wood formation (Zhong et al., 2010b), and biotic (Christianson et al., 2010; Zhu et al., 2014) and abiotic stress responses (Tran et al., 2010; Zhu et al., 2014).

There are less than 100 members of NAC TFs s discovered in *Arabidopsis*, rice, poplar, and other major angiosperm lineages (Ooka et al., 2003; Zhu et al., 2012; http://www.ncbi.nlm.nih.gov), whereas only approximate 30 NAC TFs are present in early land plants (e.g., *Selaginella moellendorffii*) (Zhu et al., 2012). Such extensive expansions were presumably mainly obtained via WGD and other small-scale duplication events (Zhu et al., 2012). Phylogenetic analyses of the NAC TFs of a diverse set of land plants revealed clearly-classified subfamilies (Ooka et al., 2003; Zhu et al., 2012). Among these NAC subfamilies, some share the evolutionary history dated before the divergence of dicots and monocots (termed non-lineage-specific subfamilies; e.g., Ia, IIIc, and Vb subfamilies), while some contain exclusively either dicot members or monocot members (termed dicot/monocot-specific subfamilies; e.g., VIa, VIb, and X subfamilies) (Zhu et al., 2012). As described in great detail by Raven and Johnson (2002), angiosperms were classified into monocots and dicots, between which the differences in morphological and physiological features were observed. The existence of lineage specific subfamilies suggested some various origins of NAC subfamilies such that they should harbor distinct evolutionary features through diverse evolutionary events (e.g., polyploidy) specific to either of the lineages. In addition, albeit the extensive functional diversification, NAC subfamilies could be classified into two major categories according to their functions, the relatively conserved processes and the relatively specific processes (Zhu et al., 2012). Thus, we would expect underlying factors that drive such functional divergence.

Taking all these observations together, it is reasonable for us to come up with the hypothesis that there should be divergent evolutionary patterns of NAC TFs both between dicots and monocots, and among NAC subfamilies as well. And the underlying mechanism should shed lights on how evolutionary divergence contributes to differences in establishing NAC gene subfamilies and thus impacts the distinct morphological and physiological features between dicots and grasses. However, few comprehensive studies have been conducted to prove this point of view. Therefore, to clarify this speculation and to present a picture on the possible evolutionary fates of NAC TFs, and more importantly to address the question of whether NAC genes with identical function evolve through specific mechanisms, we compared gene duplication and loss, evolutionary rate, and selective pattern among NAC subfamilies, as well as that between dicots and monocots. Using six dicot and five grass genomes representing diverse lineages of the flowering plants, evidence supporting our hypothesis was revealed and some subsequent deductions were made.

## Materials and methods

### Data retrieval and construction of COGs

Protein-coding transcripts of *A. thaliana* (release TAIR10, The Arabidopsis Genome Initiative), *Brassica rapa* (release v1.1) (Wang et al., 2011), *Glycine max* (release Glyma1.1, Soybean Genome Project, DoE Joint Genome Institute; Li et al., 2012), *Medicago truncatula* (release Mt3.5v4, *M. truncatula* genome sequencing project; Li et al., 2012), *Populus trichocarpa* (release Poptr JGIv3.0, The Joint Genome Institute; Li et al., 2012), *Ricinus communis* (release v0.1) (Chan et al., 2010; Li et al., 2012), *Brachypodium distachyon* (release Brachy1.2, International Brachypodium Initiative; Li et al., 2012), *Oryza sativa* (MSU Release 7.0) (Ouyang et al., 2007; Li et al., 2012), *Setaria italica* (release v2.1, DoE Joint Genome Institute), *Sorghum bicolor* (release v1.0) (Paterson et al., 2009; Li et al., 2012), and *Zea mays* (Release B73 RefGen_v2, Maize Genome Project) were downloaded from JGI (http://www.phytozome.net/) or EnsemblePlants (http://plants.ensembl.org/index.html) to construct a local BLAST database by using BLAST v2.2.27.

Multiple query NAC sequences from different lineages were used for each subfamily to obtain comprehensive samples through BLAST searches of our local database. Sequences collected from the same species with DNA identity> 95% and without indels were regarded as possible alleles (Zhang et al., 2001), thus were removed from the resulting data sets. Sequences shorter than 400 bp, as well as sequences with many ambiguous base calls were excluded from further analyses. All the non-redundant putative NAC protein sequences were manually checked for the NAC domain through CDD (http://www.ncbi.nlm.nih.gov/sites/cdd; Zhu et al., 2012), with PFAM NAM domain (PF02365) as guidance.

Clusters of Orthologous Groups (COGs; Li et al., 2012) were generated following the protocol of Li et al. (2012), which was modified from Tatusov et al. (2000) and could be used to detect orthologs among the slowly and rapidly evolving genes. The COGs reflect one-to-many and many-to-many orthologous relationships as well as simple one-to-one relationships (Tatusov et al., 2000).

### Sequence alignment and phylogenetic analysis

Protein sequences for each COG were first aligned with ClustalX v2.1 (Larkin et al., 2007) and extensively adjusted manually with BioEdit (http://www.mbio.ncsu.edu/BioEdit/BioEdit.html) when necessary. In order to objectively assess alignment quality, the column scores of each amino acid site were estimated in ClustalX v2.1 (Larkin et al., 2007) and sites with a column score greater than 12 were retained for tree reconstruction (Shan et al., 2007). Phylogenetic tree reconstruction was performed with both Maximum Likelihood (ML) and Neighbor Joining (NJ) approaches using the amino acid sequences. The ML analysis was conducted with the program PhyML version 3.0.1 (Guindon and Gascuel, 2003), using the LG model recommended by ProtTest v2.2 (Abascal et al., 2005), an estimated gamma distribution parameter, and a Shimodaira-Hasegawa-like approximate likelihood ratio test. ML searches were initiated with a BIONJ tree (Guindon and Gascuel, 2003). The PHYLIP package version 3.69 (Felsenstein, 1989) was used to perform 1000 bootstrap replicas of a NJ tree based on a JTT distance matrix.

### Estimation of gene duplication and loss events

Gene duplication and loss within each subfamily were analyzed mainly by checking each COG manually. Each COG is normally assumed to have at least two subgroups (the dicot and grass subgroup) and at least six and five members from dicot and grass species, respectively (Li et al., 2012; Figure S3). If two or more dicot or grass subgroups were detected, or if these subgroups have two or more members from the same species, we deduced that one or more duplication events happened. On the other hand, if there is a member missing from any species within the dicot or grass subgroup, it was deduced to be a gene loss event. Every gene loss was further validated by checking with GenBank (Li et al., 2012).

### Molecular evolution analysis

A codon sequence alignment of each COG was generated from its corresponding amino acid alignment using the software aa2dna (http://www.bio.psu.edu/People/Faculty/Nei/Lab/software.htm). The ratio of non-synonymous substitutions per non-synonymous site (*d*_N_) to synonymous substitutions per synonymous site (*d*_S_) (ω value) of homologous gene pairs (Li et al., 2012) was calculated using the likelihood method in Codeml from the PAML package v4.6 (Yang, 2007). Saturation effects were avoided by discarding the gene pairs for which *d*_S_ > 2 (Ramsay et al., 2009; Li et al., 2012).

The branch models embedded in Codeml could reveal the ratio variation among branches in the phylogeny and detect positive selection acting on particular lineages (Yang, 1998; Yang and Nielsen, 2002), and thus were used to test variation of the ω ratio among different branches in gene trees (Wang et al., 2015) and were compared with the one-ratio model that assumes a constant ω value across all branches using the likelihood ratio test (LRT) (Li et al., 2012). In these processes, truncated sequences were removed and tree topologies were manually adjusted (Li et al., 2012).

In order to reveal the molecular evolutionary patterns of the COGs within NAC subfamilies, we further conducted likelihood analysis on the same data sets used in *d*_N_/*d*_S_ ratio estimation previously under a nested set of codon-substitution models with Fitmodel v0.5.3 (Guindon et al., 2004), which allows changes between selection patterns to occur through time. As did in Shan et al. (2009), we obtained the trees and alignments used in the Fitmodel analyses, and the ML estimates for other parameters including branch length, substitution rate ratios (ω_1_, ω_2_, and ω_3_), and equilibrium frequencies for sites in the three rate ratio classes (*p*_1_, *p*_2_, and *p*_3_). Comparison between models of rate heterogeneity variation across sites without vs. with switching among substitution rate ratio (M3 vs. M3 + S1) was conducted, and the chi-squared test was employed to estimate the significance of difference.

## Results

### Homologs from most of the NAC subfamilies are divided into more than one COGs

**Figure 1:**
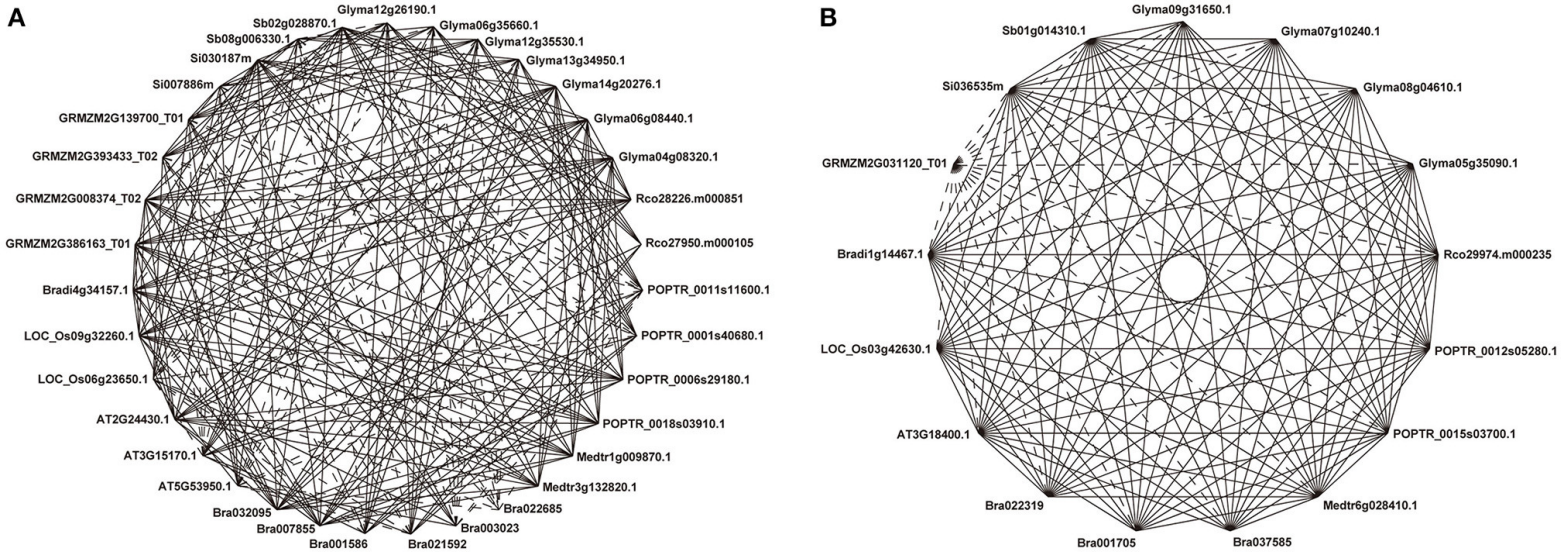
Representative COGs of the Ia subfamily. Ia_1 **(A)** and Ia_4 **(B)**. Solid lines show reciprocal BeTs (Best Hits) and broken lines show asymmetrical BeTs. Genes from the same species are adjacent. Each gene ID is indicated, and the prefix “Rco” denotes genes from *Ricinus communis*.

Our previous analysis based on 837 NAC sequences of land plants demonstrates a 21-subfamily configuration of NAC proteins, some of which are dicot-/monocot-specific (Zhu et al., 2012). To investigate the divergent evolutionary patterns of NAC genes between dicots and grasses, we focused on those subfamilies that contain both dicot and grass members in the present study. In these 13 non-lineage-specific NAC subfamilies (Ia, Ib, Ic, II, IIIa, IIIb, IIIc, IVb, IVc, IVd, Va, Vb, and VIc subfamilies), 31 COGs were obtained (Figure 1, Figure S1). Homologs from all subfamilies could be divided into more than one COGs except for IIIc, IVc, Vb, and VIc subfamilies, from which homologs are included in a single COG (Figure S1). The appearance of various COGs within one subfamily suggests more complicated divergences during the evolutionary process of such NAC subfamilies than that of others.

**Figure 2:**
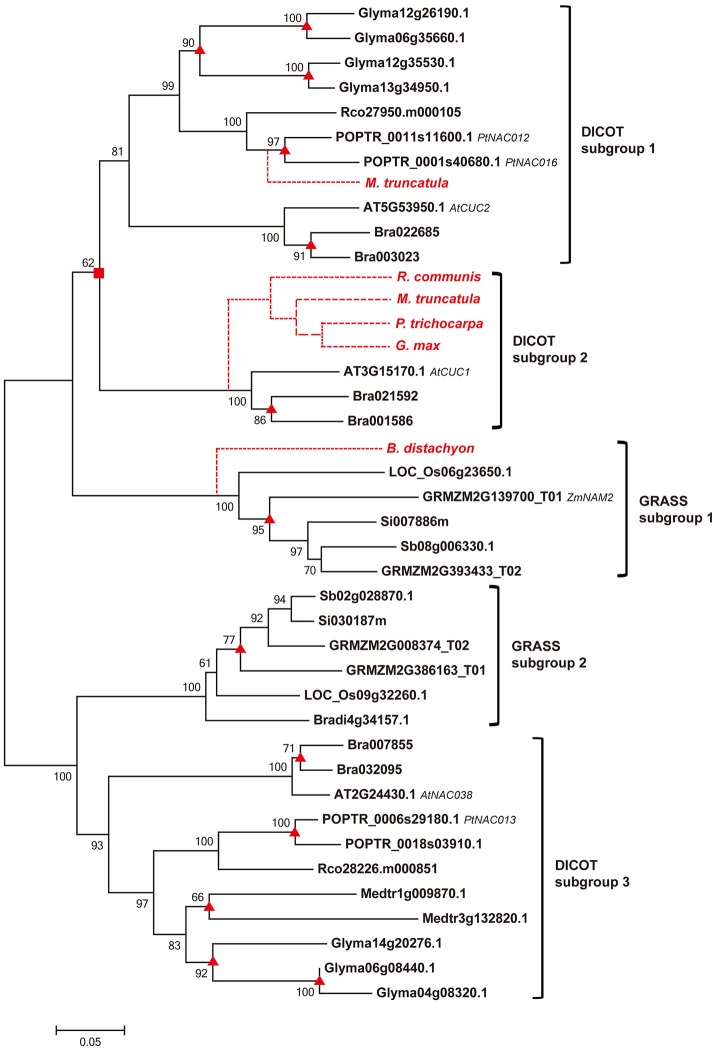
Phylogenetic relationship of sequences within the COG Ia_1 by NJ method with bootstrap support above 50% shown at the nodes. Square boxes indicate ancient duplication events; the triangles indicate recently occurred species-specific duplication events; and the broken lines indicate absent genes, either lost from those species or not yet sequenced. Gene names, based on previous references, are listed after the gene IDs.

To unravel the evolutionary relationships among homologs of COGs, we constructed phylogenetic tree for each COG by using both NJ and ML approaches. The NJ analyses show that genes from different dicot or grass species cluster together in compact clades with high support values (mostly >90% bootstrap value; Li et al., 2012) in all the phylogenetic trees (Figure 2, Figure S2). The ML analyses yielded identical results (data not shown). Based on the topology of trees, we defined dicot and grass subgroups for each COG, and the relationships among homologous genes in each subgroup are largely consistent with the angiosperm phylogeny (Figure 2, Figure S2).

### Contrasting changes in expansion of the NAC genes

**Figure 3:**
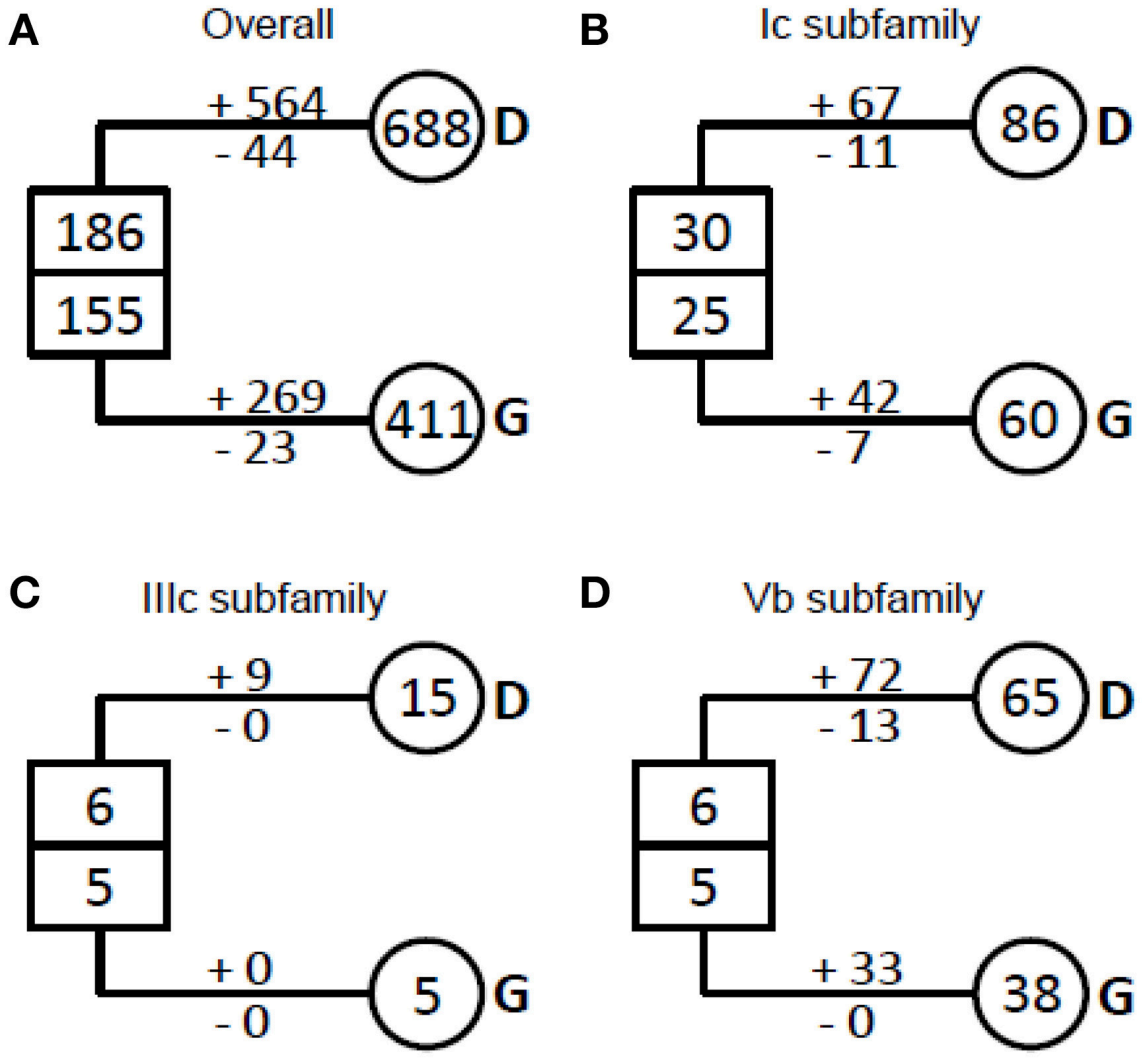
Evolutionary change of the number of NAC TFs in plants. **(A)** The overall NAC TFs examined in plants; **(B)** The Ic subfamily of the NAC TFs examined; **(C)** The IIIc subfamily of the NAC TFs examined; **(D)** The IVb subfamily of the NAC TFs examined. The numbers in circles and rectangles represent the numbers of genes in extant and ancestral species, respectively. The numbers with plus and minus signs indicate the number of gene gains and losses, respectively, for each branch (D, dicots and G, grasses).

NAC proteins were considered to have undergone extensive expansion after the divergence of vascular plants (Zhu et al., 2012). To well understand how NAC genes have evolved in angiosperms, we estimated the number of NAC genes in the most recent common ancestor (MRCA) of the extant angiosperms including dicots and grasses (Figure 3, Table 1). We noticed that there were 341 ancestral NAC genes in the MRCA of angiosperms (Figure 3A). When the numbers of ancestral genes were compared with those in the MRCA of dicots and grasses, it appeared that the NAC gene family has almost tripled in size since the divergence of dicots and grasses some 145 Mya (Davies et al., 2004; Anderson et al., 2005). However, the expansion was uneven among NAC subfamilies and between angiosperm lineages. In several subfamilies (e.g., IIIc), the number of genes remained unchanged, or nearly so, since the dicot/grass split (Figure 3C, Table 1). To the contrary, in many other subfamilies, the number had increased dramatically. In the Ic subfamily, for example, there were 86 and 60 genes in the MRCA of dicots and grasses, respectively, far more than the estimated number of genes (55 in total) in the MRCA of angiosperms (Figure 3B, Table 1). Dicots and grasses have gained 67 and 42 genes and lost 11 and 7 genes, respectively, in total, since their splits (Figure 3B). Clearly, the number of genes gained in the dicot lineages was much greater than that gained in the grass lineages (Figure 3, Table 1).

**Table 1:** Gene duplication and loss in each of the NAC TFs COGs/subfamilies.

COG/Subfamily^*^	**Gene duplication**				**Gene loss**	
	**Ancient**		**Recent**			
	**Dicot-specific**	**Grass-specific**	**Dicot species-specific**	**Grass species-specific**	**Dicot-specific**	**Grass-specific**
**Ia/NAM/CUC3**^(i)^						
Ia_1	2	1	11	2	5	1
Ia_2	–	2	10	4	–	–
Ia_3	2	2	18	3	7	–
Ia_4	–	–	3	–	–	1
Ia_5	–	–	6	–	–	–
**Ib/NAC1**^(i)^						
Ib_1	–	2	4	1	–	–
Ib_2	–	3	6	3	2	4
**Ic/SND**^(i)^						
Ic_1	3	4	16	3	8	3
Ic_2	–	1	3	2	–	–
Ic_3	–	1	10	–	–	3
Ic_4	1	1	10	1	3	1
Ic_5	–	–	4	1	–	–
**II/ONAC4**^(i)^						
II_1	2	1	10	–	7	1
II_2	–	1	5	3	1	–
II_3	1	2	6	1	4	1
II_4	–	–	5	3	–	–
**IIIa/TIP**^(ii)^						
IIIa_1	1	1	11	1	2	–
IIIa_2	–	–	7	1	–	–
**IIIb/NAC2**^(i)^						
IIIb_1	5	4	28	1	8	1
IIIb_2	–	1	10	1	–	1
IIIb_3	–	–	10	–	2	–
IIIc/ANAC11^(ii)^	–	–	9	–	–	–
**IVb/ANAC34**^(i)^^(ii)^						
IVb_1	–	2	4	2	1	–
IVb_2	1	1	4	–	4	–
IVc/TERN^(i)^	1	2	13	2	3	–
**IVd/ONAC22**^(i)^^(ii)^						
IVd_1	–	2	11	1	–	2
IVd_2	1	2	11	1	1	1
**Va/NAP**^(i)^^(ii)^						
Va_1	–	2	6	1	–	2
Va_2	–	–	7	3	–	–
Vb/SNAC^(ii)^	6	6	36	3	13	–
VIc/ONAC6^(i)^	1	1	10	–	3	1
TOTAL	27	45	324	44	74	23

* *(i) and (ii) designate categories of NAC subfamilies, where (i) represents those involved in the relatively conserved processes (such as embryogenesis, cell division, seedling development, floral development, and senescence) and (ii) represents those involved in the relatively specific processes (such as biotic and abiotic stress responses)*.

### Distinct patterns of gene duplication and loss between dicots and grasses

To obtain detailed insights into the different duplication and loss patterns of NAC genes between dicots and grasses, we defined two types of duplication events, (a) the ancient duplications which occurred in the MRCA of dicot (dicot-specific duplication) or grass (grass-specific duplication) before their divergence, and (b) the recent duplications which are specific to a particular organismal lineage (one or few closely related species) of dicot (dicot species-specific duplication) or grass (grass species-specific duplication). We separately examined these two types of duplications in each one of the 31 COGs, and summarized the results in Table 1, Figure 2, and Figure S2.

Our results demonstrate that most COGs (23/31) have undergone one or more ancient duplications, of which more occurred in grass (45 in total) than in dicot (27 in total) (Table 1). As to the recent duplications, the highest frequency in grass COGs is 4 times in Ia_2, while it occurred no less than 4 times in nearly all the COGs of dicot (except for Ia_4 and Ic_2), with the highest frequency 36 in Vb COG (subfamily). Given the evidence that recent polyploidization events are often observed in the dicot *Glycine max*, having the most recent event occurred 13 Mya (Schmutz et al., 2010), NAC genes in grasses tend to diverge through ancient duplications, most probably from whole genome duplication events in the MRCA of grasses before their divergence, while dicot NAC genes experienced more recent duplication events within specific dicot species.

According to their functions, NAC subfamilies were previously classified into two major categories, one involved in the relatively conserved processes (category 1) and the other involved in the relatively specific processes (category 2) (Zhu et al., 2012). We noticed that subfamilies in category 1 (Ia, Ib, Ic, II, IIIb, IVc, and VIc subfamilies) often experienced more ancient duplications than those from category 2 (IIIa, IIIc, and Vb subfamilies). Moreover, some NAC subfamilies appeared before the divergence of angiosperms (Ia, Ic, IIIc, IVb, IVd, and Vb subfamilies; Zhu et al., 2012). Therefore, it is not surprising to observe more ancient duplications in these older subfamilies than in the recently diverged ones.

We also found that dicot-specific gene losses occurred in nearly all the subfamilies analyzed and majority of the missing members are from *Medicago truncatula* (Table 1, Figure 2, and Figure S2; Li et al., 2012). By contrast, the grass-specific gene losses only occurred in the Ia, Ib, Ic, II, IIIb, IVd, and VIc subfamilies, all of which contain only category 1 genes, except for subfamily IVd. Obviously, there are fewer gene losses in the grass than that in the dicots (Table 1).

### More rapid evolution of NAC genes occurred in grasses

To understand whether the NAC genes from grasses and dicots are under different evolutionary constraints, we applied two approaches to estimate the ω values of each COG, i.e., the pairwise comparison and the branch model.

**Figure 4:**
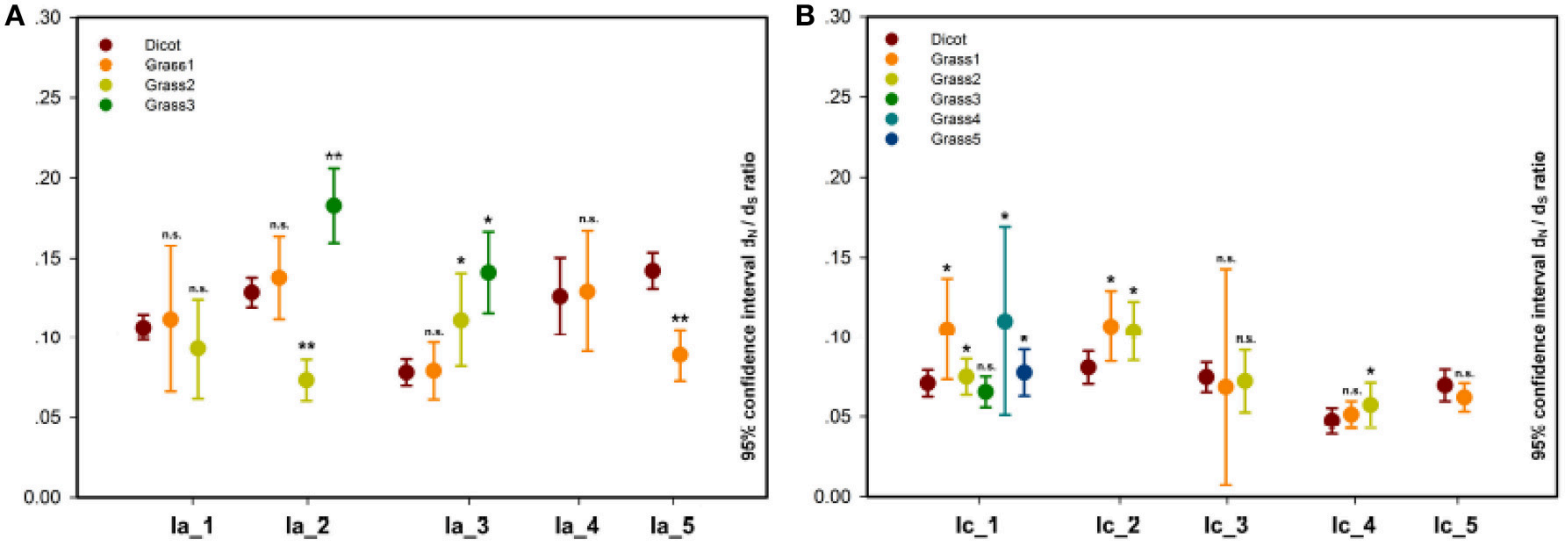
Pairwise estimation of ω values in each dicot or grass subgroup within subfamilies. **(A)** Ia_1-5; **(B)** Ic_1-5. In each of the subfamilies, a significance test on the difference between dicot and grass subgroup was carried out, and its result, listed in Table S1, is indicated by n.s. (not significant), ^*^P < 0.05, and ^**^P < 0.01.

In the pairwise comparison approach, we calculated the pairwise ω values within each grass or dicot subgroup and compared their averages between these two subgroups. The result showed that the average ω values of 30 grass subgroups (subgroup 3 of Ia_2, subgroups 2 and 3 of Ia_3/II_3, subgroups 1, 2, and 3 of Ib_1, subgroups 3 and 4 of Ib_2, subgroups 1, 2, 4, and 5 of Ic_1, subgroups 1 and 2 of Ic_2/II_2/IIIb_2, subgroups 2 of Ic_3/Ic_4/IVd_1, subgroups 1 of II_4/IIIa_1/IIIa_2/IIIb_3, subgroups 2 and 5 of IIIb_1, and subgroups 1 and 7 of Vb) are significantly higher than that of the corresponding subfamilies composed of the homologs from the dicot; and 11 grass subgroups (subgroup 2 of Ia_2/IVb_1, subgroup 1 of Ia_5/II_1/IVc/Va_1/Vb_2/VIc, subgroup 4 of IIIb_1, and subgroup 1 and 2 of IVb_2) show the opposite result (Figure 4, Figure S3, and Table S1).

In order to examine changes in the evolutionary constraints across different subgroups, we also applied the branch-specific model to the phylogenetic trees of each COG because evolution rate of the most recent common ancestor in the subgroup is not taken into account in the pairwise comparison approach. In this approach, the model assumes two or more ω ratios, which correspond to the number of subgroups (Yang, 2007; Li et al., 2012). The result indicated that this model was favored over the one-ratio model by the LRT (*P* < 0.05) test in all the phylogenetic trees (Table 2; Li et al., 2012). The ω values of the subgroups estimated from branch model confirm most of the result derived from the pairwise comparison approach (Table 2; Li et al., 2012). More importantly, when the most recent common ancestor is involved (e.g., subgroup 2 of Ia_2 or subgroup of Ia_5), some of the grass subgroups that exhibited a lower ω value than their dicot subgroups in the pairwise comparison approach demonstrated a higher ω value. It is worth mentioning that homologous NAC genes from nearly all the grass subgroups with a lower ω value than their corresponding dicot subgroups are involved in the relatively conserved biological processes (category 1).

**Table 2:** Statistics of the parameters from branch model of PAML.

**Subfamily/COG**	**Null hypothesis**		**Alternative hypothesis**	
	**−ln *L***	**ω**	**−ln *L***	**ω**
**Ia/NAM/CUC3**				
Ia_1	8,386.28	0.099	8,385.35	Dicot = 0.094; Grass1 = 0.120; Grass2 = 0.101
Ia_2	8,992.08	0.152	8,989.04	Dicot = 0.136; Grass1 = 0.192; Grass2 = 0.150; Grass3 = 0.163
Ia_3	7,245.47	0.129	7,202.71	Dicot = 0.087; Grass1 = 0.158; Grass2 = 0.215; Grass3 = 0.328
Ia_4	6,369.06	0.174	6,364.97	Dicot = 0.155; Grass = 0.248
Ia_5	6,161.63	0.181	6,159.19	Dicot = 0.165; Grass = 0.228
**Ib/NAC1**				
Ib_1	7,596.32	0.128	7,584.47	Dicot = 0.108; Grass1 = 0.113; Grass2 = 0.205; Grass3 = 0.092
Ib_2	7,130.28	0.125	7,127.05	Dicot = 0.111; Grass1 = 0.139; Grass2 = 0.093; Grass3 = 0.162; Grass4 = 0.138
**Ic/SND**				
Ic_1	7,504.70	0.060	7,487.05	Dicot = 0.049; Grass1 = 0.048; Grass2 = 0.061; Grass3 = 0.033; Grass4 = 0.109; Grass5 = 0.107
Ic_2	7,064.77	0.129	7,057.95	Dicot = 0.103; Grass1 = 0.148; Grass2 = 0.173
Ic_3	5,570.71	0.089	5,560.92	Dicot = 0.087; Grass1 = 0.197; Grass2 = 0.057
Ic_4	7,536.80	0.093	7,519.97	Dicot = 0.072; Grass1 = 0.120; Grass2 = 0.162
Ic_5	5,354.24	0.088	5,351.12	Dicot = 0.078; Grass = 0.120
**II/ONAC4**				
II_1	7,819.42	0.096	7,815.66	Dicot = 0.105; Grass1 = 0.062; Grass2 = 0.082
II_2	7,137.84	0.220	7,126.78	Dicot = 0.169; Grass1 = 0.320; Grass2 = 0.252
II_3	5,299.31	0.061	5,209.24	Dicot = 0.019; Grass1 = 0.043; Grass2 = 0.129; Grass3 = 0.236
II_4	9,105.86	0.196	9,104.73	Dicot = 0.186; Grass = 0.223
**IIIa/TIP**				
IIIa_1	9,158.06	0.150	9,155.96	Dicot = 0.142; Grass1 = 0.195; Grass2 = 0.157
IIIa_2	9,005.51	0.196	8,985.61	Dicot = 0.158; Grass = 0.361
**IIIb/NAC2**				
IIIb_1	8,008.27	0.041	7,986.81	Dicot = 0.044; Grass1 = 0.021; Grass2 = 0.048; Grass3 = 0.002; Grass4 = 0.051; Grass5 = 0.081
IIIb_2	10,374.08	0.206	10,373.51	Dicot = 0.200; Grass1 = 0.196; Grass2 = 0.226
IIIb_3	11,422.90	0.229	11,418.33	Dicot = 0.211; Grass = 0.300
IIIc/ANAC11	5,054.30	0.115	5,032.59	Dicot = 0.085; Grass = 0.241
**IVb/ANAC34**				
IVb_1	7,100.19	0.112	7,092.41	Dicot = 0.121; Grass1 = 0.153; Grass2 = 0.116; Grass3 = 0.079
IVb_2	6,228.24	0.124	6,220.74	Dicot = 0.144; Grass1 = 0.152; Grass2 = 0.081
IVc/TERN	7,762.88	0.179	7,759.01	Dicot = 0.160; Grass1 = 0.196; Grass2 = 0.227; Grass3 = 0.232
**IVd/ONAC22**				
IVd_1	9,018.96	0.138	9,018.52	Dicot = 0.143; Grass1 = 0.132; Grass2 = 0.143; Grass3 = 0.122
IVd_2	7,229.97	0.097	7,227.56	Dicot = 0.101; Grass1 = 0.109; Grass2 = 0.108; Grass3 = 0.073
**Va/NAP**				
Va_1	7,207.26	0.182	7,198.03	Dicot = 0.144; Grass1 = 0.230; Grass2 = 0.259
Va_2	6,352.65	0.172	6,346.95	Dicot = 0.143; Grass = 0.226
Vb/SNAC	14,926.52	0.043	14,895.68	Dicot = 0.037; Grass1 = 0.090; Grass2 = 0.018; Grass3 = 0.082; Grass4 = 0.055; Grass5 = 0.087; Grass6 = 0.042; Grass7 = 0.083
VIc/ONAC6	6,302.10	0.180	6,300.73	Dicot = 0.194; Grass1 = 0.144; Grass2 = 0.167

### Differential selection between dicots and grasses

To assess the substitution process of these NAC subfamilies, we performed likelihood analyses by using Fitmodel (Guindon et al., 2004), which takes into account the variability of selection regimes across sites and lineages, and compared the sites under positive selection between the grass and the dicot subgroups within each phylogenetic tree of COG. Table 3 shows that log likelihoods improved significantly (*P* < 0.001) as parameters were added to the nested substitution models (Table 3), suggesting that the M3+S1 model was favored over the M3 model for each of data sets analyzed in our study. The parameter estimates of the M3+S1 model demonstrate that 66% sites (*p*_1_ + *p*_2_) are identified to be under purifying selection with ω_1_ < 0.023 and ω_2_ < 0.724 in all the COGs (Table 3). On the other hand, 2 to 22% (*p*_3_) of sites within the genes in COGs are identified to be under positive selection with ω_3_ values considerably larger than 1.0. Among these positive selection sites, the number of sites for genes in COGs of Ib_1, II_2, Va_1, Va_2, and Vb is significantly asymmetric across dicot and grass families, suggesting that the selection mainly occurred either in the dicot or in the grass subclade of these phylogenetic trees. This result provides evidence of divergent selection between dicots and grasses (Table 3). Since ω_3_ values approaching 1.0 are described as indicative of relaxed selection, we cannot discount the possibility that positive selection may also have driven evolution in the COGs of Ia_4, II_1, IIIa_1, IIIb_3, and IVd_2, although no or slightly positive selection sign (ω_3_ < 1.096) was observed (Table 3). Functionally critical (i.e., adaptive) substitutions at sites in the ω_3_ class could be identified experimentally.

**Table 3:** Likelihood ratio test statistic and parameters estimated from the M3 and M3 + S1 models in Fitmodel.

**Subfamily/COG**	**M3 Model^a^ (Heterogeneity across sites)**				**M3 + S1 Model (Shifting across branches)**				**Positive sites (M3 + S1 Model)**	
	**−ln *L***	***p*_1_** **ω_1_**	***p*_2_** **ω_2_**	***p*_3_** **ω_3_**	**−ln *L***	***p*_1_** **ω_1_**	***p*_2_** **ω_2_**	***p*_3_** **ω_3_**	**Dicots specific**	**Grasses specific**
**Ia/NAM/CUC3**										
Ia_1	7,987.10	0.488	0.263	0.249	7,945.83^**^	0.587	0.231	0.181	153, 154, 159, 167	30, 172, 173, 177, 202
		0.001	0.081	0.437		<0.001	0.103	0.671		
Ia_2	8,695.22	0.505	0.305	0.191	8,617.30^**^	0.595	0.258	0.147	33, 155, 166, 170, 176, 177, 180	135, 161, 172, 173, 187, 188, 174, 175
		0.014	0.225	0.957		<0.001	0.243	1.658		
Ia_3	7,089.74	0.548	0.377	0.075	7,029.95^**^	0.614	0.308	0.079	136	120, 135
		0.024	0.242	0.997		0.001	0.335	2.041		
Ia_4	6,151.02	0.472	0.286	0.242	6,140.29^**^	0.513	0.246	0.241	11, 178, 185, 189, 208, 220, 245, 255	182, 207, 208, 228
		0.009	0.235	0.925		0.003	0.207	1.096		
Ia_5	5,945.22	0.518	0.233	0.249	5,903.54^**^	0.665	0.276	0.059	—	225
		0.014	0.281	0.863		0.011	0.699	3.616		
**Ib/NAC1**										
Ib_1	7,286.55	0.498	0.202	0.299	7,241.32^**^	0.641	0.236	0.123	88, 193	94, 126, 141, 145, 170, 180, 183, 191
		0.006	0.118	0.489		0.005	0.290	1.152		
Ib_2	6,771.42	0.624	0.263	0.113	6,745.78^**^	0.671	0.261	0.068	155, 162	159, 181, 166, 178
		0.013	0.274	0.906		0.006	0.350	2.233		
**Ic/SND**										
Ic_1	9,314.31	0.592	0.318	0.091	9,266.30^**^	0.735	0.221	0.044	163	145, 151
		0.007	0.111	0.488		0.005	0.206	1.274		
Ic_2	6,893.55	0.537	0.334	0.128	6,873.30^**^	0.589	0.282	0.129	—	167, 170, 176, 177, 202, 204
		0.009	0.235	0.979		<0.001	0.240	1.193		
Ic_3	5,718.94	0.682	0.243	0.075	5,711.67^**^	0.705	0.218	0.077	183	162, 164, 171
		0.014	0.259	0.997		0.008	0.279	1.151		
Ic_4	7,459.08	0.644	0.276	0.080	7,419.25^**^	0.732	0.120	0.068	144, 149, 169, 217	199, 200, 140, 142
		0.012	0.237	1.088		0.009	0.369	1.728		
Ic_5	5,286.13	0.520	0.224	0.257	5,272.27^**^	0.670	0.286	0.015	167	—
		0.006	0.111	0.470		0.007	0.464	2.997		
**II/ONAC4**										
II_1	7,535.33	0.635	0.225	0.140	7,527.86^**^	0.670	0.203	0.128	16, 146, 207	—
		0.009	0.187	0.738		0.005	0.204	0.867		
II_2	7,642.77	0.227	0.498	0.235	7,606.47^**^	0.379	0.400	0.220	8, 11, 13, 14, 15, 16, 35, 67, 129, 145, 146, 202, 204, 205, 207, 208, 210	22, 31, 73, 144, 167, 225
		0.009	0.225	0.784		<0.001	0.233	1.138		
II_3	7,027.97	0.568	0.289	0.142	7,003.30^**^	0.621	0.211	0.168	2, 206, 227	58, 211, 212, 213
		0.010	0.129	0.532		0.002	0.147	0.633		
II_4	8,916.62	0.411	0.399	0.190	8,781.10^**^	0.673	0.285	0.042	249, 342	—
		0.025	0.265	0.860		0.015	0.724	>>1		
**IIIa/TIP**										
IIIa_1	9,189.64	0.422	0.278	0.301	9,160.07^**^	0.496	0.293	0.211	161, 163, 165, 175	100, 159, 162, 187, 189, 195
		0.007	0.137	0.589		0.002	0.186	0.852		
IIIa_2	8,769.59	0.290	0.352	0.358	8,726.21^**^	0.349	0.315	0.337	171, 196, 219, 226, 230, 231, 238	157, 158, 222, 234, 236, 239, 241
		0.006	0.113	0.615		0.002	0.101	0.781		
**IIIb/NAC2**										
IIIb_1	11,715.75	0.666	0.309	0.025	11,609.68^**^	0.685	0.266	0.049	127	92
		0.010	0.109	0.562		0.002	0.123	0.614		
IIIb_2	9,955.97	0.298	0.342	0.360	9,913.68^**^	0.548	0.240	0.211	183, 201, 203, 213, 218, 238	176, 185,196, 200, 204, 215
		0.011	0.133	0.682		0.023	0.315	1.233		
IIIb_3	11,351.55	0.327	0.337	0.336	11,319.73^**^	0.408	0.281	0.311	4, 176, 177, 179, 201, 250, 259, 280, 337, 339	—
		0.016	0.218	0.717		0.006	0.242	0.885		
IIIc/ANAC11	5,331.14	0.482	0.337	0.180	5,280.02^**^	0.578	0.246	0.176	2, 98, 110, 162, 176, 178, 181, 183, 152, 162, 163	150, 153, 154, 155, 158, 160, 165, 168, 169, 170, 173, 174, 177, 184
		0.007	0.160	1.051		<0.001	0.157	1.613		
**IVb/ANAC34**										
IVb_1	7,232.44	0.587	0.238	0.174	7,188.84^**^	0.655	0.209	0.136	190, 191, 192	2, 172, 196, 197, 201
		0.011	0.224	0.823		0.002	0.218	1.324		
IVb_2	6,123.96	0.404	0.399	0.197	6,086.42^**^	0.583	0.286	0.131	1, 3, 176	157, 163, 185
		0.003	0.122	0.587		<0.001	0.188	1.137		
IVc/TERN	7,932.92	0.581	0.298	0.121	7,898.20^**^	0.641	0.260	0.098	149	42
		0.038	0.400	1.115		0.021	0.487	1.628		
**IVd/ONAC22**										
IVd_1	9,087.07	0.483	0.267	0.250	9,072.53^**^	0.520	0.255	0.226	181, 187, 197, 210, 220, 234	178, 179, 183, 199, 206, 213, 229
		0.007	0.167	0.711		0.002	0.173	0.834		
IVd_2	7,912.34	0.535	0.279	0.186	7,896.29^**^	0.605	0.272	0.123	143, 50, 126, 146, 147, 148, 149, 150, 154	153, 158
		0.006	0.152	0.717		0.002	0.191	1.026		
**Va/NAP**										
Va_1	6,872.05	0.488	0.308	0.204	6,833.05^**^	0.566	0.285	0.148	95, 150, 156, 184, 185, 186, 187, 190, 191, 201, 205, 209	162, 164, 197, 206, 208
		0.007	0.239	1.030		<0.001	0.335	1.746		
Va_2	6,093.17	0.466	0.282	0.252	6,066.48^**^	0.544	0.252	0.203	116, 148, 150, 195, 196, 201	163, 191, 202, 208, 133, 156, 158, 159, 209
		0.006	0.177	0.892		0.004	0.253	1.260		
Vb/SNAC_11	15,736.23	0.594	0.229	0.177	15,647.48^**^	0.612	0.222	0.165	52	28, 47, 50, 80, 115, 148, 151, 152
		0.003	0.067	0.233		<0.001	0.054	0.349		
VIc/ONAC6	6,176.96	0.382	0.533	0.086	6,159.23^**^	0.525	0.428	0.046	114	82
		0.027	0.262	0.887		0.006	0.395	2.033		

a *p_i_ proportion of sites that fall into the ω_i_ site class, i = 1, 2, 3*.

** *P < 0.01, and the probabilities are obtained by LRT (df = 2) of the models M3 and M3 + S1*.

## Discussion

In the present study, we were seeking evidence of divergent evolutionary patterns among NAC TFs by comparing serial evolutionary features of non-lineage specific NAC subfamilies and uncovered patterns of duplication and diversification in each subfamily. As a result, several distinct evolutionary features were observed, (1) uneven constitution of COGs and contrasting birth/death rates among subfamilies, (2) distinct patterns of gene duplication and loss between dicots and grasses, (3) a relatively higher evolution rate in grass than in dicot in most subfamilies analyzed, and (4) asymmetrical selection pattern between dicots and grasses. Clearly, with these findings, a general picture has emerged for the mode, tempo, and consequence of the evolution of NAC TFs during the angiosperm evolution, and several relevant conclusions can now be addressed.

### Different scenarios for NAC TFs evolution in dicots and grasses

We have demonstrated that in a given COG the copy number of grass genes is always less than that of dicot genes. This phenomenon might attribute to two observations. First, dicots underwent more recent duplications. Comparative analyses have revealed up to two additional rounds of recent genome duplication in *B. rapa* after its divergence from *A. thaliana*: one triplication that arose approximately 13 Mya (Rana et al., 2004; Mun et al., 2009), and one allopolyploidization (Mun et al., 2009; Wang et al., 2011). A very recent WGD was also observed in *G. max* (Schmutz et al., 2010). Second, Ohno (1970) pointed out that following gene duplications a relaxed constraint at some sites was expected. This is very much concordant with our observations that NAC genes always show lower ω values in dicot species within one COG/subfamily. This conclusion is favored when we consider only the COGs/subfamilies with contrasting numbers of recent duplications. Meanwhile, some studies have also showed that genes having low evolution rate tend to stay in the species after gene duplication and might be more easily sub-functionalized (Davis and Petrov, 2004; Brunet et al., 2006; Semon and Wlofe, 2008).

The above observations guaranteed dicots recruited more members of NAC TFs than monocots but usually with a lower evolution rate within certain COGs/subfamilies. Therefore, two diverse evolutionary scenarios can be proposed, the relatively more copies of NAC TFs in dicots might interact with each other as part of a network through sub-functionalization, on the other hand, with high evolution rate, grass NAC TFs were always accompanied by diversifying selection (positive selection). In fact, the poplar PtrWND TFs (Ic subfamily) interact with each other and perform a diverse combination of heterodimers together with their downstream TFs to form a transcriptional network, thus activating the secondary wall biosynthetic program during wood formation (Zhong et al., 2011). In contrast with dicots, the frequent diversifying selection in grass history and following relaxed selection constraints might imply the increased allelic diversity at some sites and increased opportunity for novel gene interactions. For instance, our another study on allelic variation of *NAM-B1* gene (Va subfamily) through 41 populations of wild emmer wheat revealed great diversity in *NAM-B1* locus both between and within populations. Meanwhile, the polymorphism of *NAM-B1* gene was significantly correlated with multiple eco-environmental factors (unpublished data). Thus, it is possible that the *NAM-B1* divergence may contribute to fitness as an adaptive evolution strategy to novel ecological challenges.

### Newly-born NAC TFs contribute to the evolution complexity of the higher plants

Several NAC subfamilies possessed stable copy number, but many others had more dynamic birth-and-death evolution, much higher birth rate than death rate (Table 1, Figure 3). Moreover, in some subfamilies, the estimated birth rates are obviously higher than that for other well-known rapidly-duplicating genes, such as Type I MADS-box genes (Nam et al., 2004), SKP1-like genes (Kong et al., 2007), and F-box genes (Xu et al., 2009). The obviously high birth rates elicited the question why plants have maintained so many new genes during the evolution process.

The possible explanation is that members of the rapidly-duplicating gene subfamilies interact with variable and/or changing targets. As shown previously, the C-terminal regions of NAC proteins, essential for target recognition, are highly variable even within the same subfamily (Ooka et al., 2003; Zhu et al., 2012). The most direct evidence could be addressed in Vb subfamily. Vb subfamily is one of the three most ancient subfamilies (Ia, Ic, and Vb subfamilies) and had been established since the origin of early-diverged land plant *Physcomitrella patens* (Zhu et al., 2012). Our results showed that all of the members in Vb subfamily were included in one COG, suggesting an extraordinarily high birth rate from a single ancestral sequence (Figure 3D, Table 1). The unusual increase of the gene number in Vb subfamily might be because of their function in balancing the cross talk between stress-related networks or pathogen-borne targets (see discussion below) in a precisely controlled fashion because almost all the members in this family contribute to response to biotic and abiotic stresses.

Coincidentally, the NAC TFs from Ic subfamily were found to function as master switches in the secondary wall biosynthesis, one of the pivotal features that vascular plants have evolved to enable themselves to conquer dry land around 430 Mya (Raven and Johnson, 2002; Zhong et al., 2010a). Genetic and functional analyses of secondary wall NACs (SWNs) in diverse species have indicated that the SWN-mediated activation of the secondary wall biosynthetic process is a conserved mechanism (Zhong et al., 2010b) in both seedless vascular plants (e.g., *S. moellendorffii*) and seed plants, including gymnosperms and angiosperms (Zhong et al., 2010b). Our results demonstrate that the SWN homologs exist in both herbaceous plants, such as *Arabidopsis*, and woody poplar; and the number of SWNs in poplar is nearly twice of *Arabidopsis*. This increase in the number of SWNs in poplar was probably due to a salicoid duplication event, a WGD event that occurred near the emergence of Salix and Populus lineages 60-65 Mya (Tuskan et al., 2006). Given the fact that homologs of SWNs in poplar display a different transcriptional program regulating the biosynthesis of secondary walls during wood formation apart from their *Arabidopsis* counterparts (Zhong et al., 2011), this might imply that woody angiosperm plant lineages have co-opted the pre-existing ancestral NAC genes, through duplication and specialization, into new functions to coordinate the activation of the secondary wall biosynthesis in wood formation.

However, several studies suggest that the number of genes in a family initially depends on their functional requirement. However, after the number reached a sufficient level, the number can fluctuate occasionally (Nei, 2007; Nei et al., 2008). In our case, NAC TFs share relatively identical function within the same subfamily (Zhu et al., 2012). Although some recently duplicated NAC genes were found to be functionally redundant (Hasson et al., 2011; Zhong et al., 2011), which suggests that small gene number changes do not necessarily change in the physiological requirement, we still cannot exclude the possibility that the gain or loss of some NAC genes, at least in some subfamilies, were due to random events.

### High evolution rates drive NAC TFs into versatile functions

The whole-genome expression profiling studies in *Arabidopsis* and rice have revealed NAC genes to be induced by at least one type of abiotic stress such as salinity, drought, cold, or abscisic acid (Fang et al., 2008; Kawaura et al., 2008), whereas in several other species, NAC genes were also shown to be induced by jasmonic acid, salicylic acid, and ethylene (Tran et al., 2004; Nakashima et al., 2007; Xia et al., 2010; Yoshii et al., 2010). Meanwhile, NAC proteins were also proven to have an affirmative role in the regulation of plant defense against different pathogens as well as wounding and insect feeding (Lin et al., 2007; Wang et al., 2009; Xia et al., 2010). Members involved in these stress-response processes include all the COGs of IIIa, IIIc, IVb, IVc, IVd, Va, and Vb subfamilies and part of COGs in Ia, Ib, II, and IIIb subfamilies.

Among the stress-related NAC subfamilies, the Vb subfamily possessed the smallest ω value, with >9 sites evolving under relaxed selection, suggesting that members of this subfamily are more conserved than others. It was shown that members of Vb subfamily tend to function at the convergence point of abiotic stress signaling and pathogen defense that was induced by multiple stresses (Fujita et al., 2004; Delessert et al., 2005; Jensen et al., 2008; Puranik et al., 2012), suggesting members of Vb subfamily are key factors involved in cross talk between the abiotic- and biotic-stress cellular network (Fujita et al., 2009). Thus, it makes sense to conclude that changes in one key element of a specific network can be constrained by its interactions with other elements, which lead to a relatively low evolution rate and less variable function of members in the Vb subfamily. On the contrary, members from other stress-related subfamilies (e.g., II, IIIa, IIIb, and IVb subfamilies) have integrated responses to environmental stresses into modulation of plant development processes, such as lateral root development (He et al., 2005; Hao et al., 2011; Puranik et al., 2012; Wei et al., 2016), seed germination (Kim et al., 2008; Seo et al., 2010; Wei et al., 2016), flowering (Kim et al., 2007; Wei et al., 2016), and senescence (Balazadeh et al., 2010; Seo and Park, 2011; Yang et al., 2011). Such versatility of NAC functions might result in greater ω values and more sites under positive selection in order to ensure plants' longevity, survival, and reproductive success under various environmental stresses.

Another example emerges from the evolutionary process of *CUCs*, all of which belong to Ia subfamily. After the *CUC3* gave birth to *CUC1/2* through gene duplication event at the base of extant seed plants (Vialette-Guiraud et al., 2011), the novel *CUCs* evolved differentially since the initial duplication. *CUC2* did not diverge much from the ancestral gene, while *CUC1* evolved more profoundly from its ancestor through neo-functionalization (Hasson et al., 2011). Meanwhile, Ia subfamily genes appear to have obtained novel functions in defense responses while retaining their ancestral function in the establishment of the shoot organ boundaries (Aida et al., 1997; Yamaguchi et al., 2008; Takeda et al., 2011).

## Conclusion

In this study, we reported divergent evolutionary patterns of NAC TFs, both among subfamilies and between dicots and grasses. Observations also showed that the distinct fates of NAC TFs are associated with plant lineage diversification and gene duplication events. Considering our results along with functional data, we hypothesize that rapidly born NAC TFs and relaxed selection in certain NAC COGs/subfamilies may have permitted substitutions that are responsible for functional divergence into new lineages. Meanwhile, the high evolution rates have increased complexity of interaction of NAC TFs following the concerted duplications from a MRCA of all the extant angiosperms.

## Author contributions

XJ and JR were in charge of data collection, analyses, and manuscript drafting and finalizing. EN and XY worked on revising and finalizing the manuscript. JP took care of the whole project, i.e., applying for grants, designing the experiments, outlining and finalizing the manuscript. All relevant authors and institutions have approved the submission for publication of this manuscript; and all persons entitled to authorship have been so named and have agreed to the submitted version of the manuscript.

### Conflict of interest statement

The authors declare that the research was conducted in the absence of any commercial or financial relationships that could be construed as a potential conflict of interest.
