# Supplementary material for: Divergent Evolutionary Patterns of NAC Transcription Factors Are Associated with Diversification and Gene Duplications in Angiosperm
Source: Front Plant Sci. 2017 Jun 30;8:1156. doi: 10.3389/fpls.2017.01156 (PMC5492850; doi:10.3389/fpls.2017.01156)
Supplement: Supplementary file 1 [file Table1.DOCX]

**Table S1** Significance test for *ω* value between dicot and grass subgroups

| COG | Subgroup | Mean | 95% Confidence Intervals | | P-value^a^ |
| --- | --- | --- | --- | --- | --- |
|  |  |  | **Lower** | **Upper** |  |
| Ia_1 | dicot | 0.107±0.044 | 0.098 | 0.115 |  |
|  | grass1 | 0.112±0.060 | 0.066 | 0.158 | n.s. (0.935) |
|  | grass2 | 0.093±0.057 | 0.061 | 0.124 | n.s. (0.124) |
| Ia_2 | dicot | 0.128±0.038 | 0.119 | 0.138 |  |
|  | grass1 | 0.138±0.016 | 0.112 | 0.164 | n.s. (0.454) |
|  | grass2 | 0.073±0.023 | 0.060 | 0.086 | ** |
|  | grass3 | 0.183±0.033 | 0.159 | 0.206 | ** |
| Ia_3 | dicot | 0.078±0.075 | 0.070 | 0.086 |  |
|  | grass1 | 0.079±0.025 | 0.061 | 0.097 | n.s. (0.184) |
|  | grass2 | 0.111±0.041 | 0.082 | 0.141 | * (0.002) |
|  | grass3 | 0.141±0.010 | 0.116 | 0.167 | * (0.012) |
| Ia_4 | dicot | 0.126±0.071 | 0.102 | 0.150 |  |
|  | grass | 0.129±0.036 | 0.091 | 0.167 | n.s. (0.553) |
| Ia_5 | dicot | 0.142±0.046 | 0.131 | 0.153 |  |
|  | grass | 0.079±0.012 | 0.072 | 0.105 | ** |
| Ib_1 | dicot | 0.111±0.048 | 0.101 | 0.150 |  |
|  | grass1 | 0.123±0.016 | 0.166 | 0.221 | ** |
|  | grass2 | 0.194±0.048 | 0.173 | 0.229 | ** |
|  | grass3 | 0.137±0.055 | 0.136 | 0.207 | * (0.005) |
| Ib_2 | dicot | 0.118±0.039 | 0.102 | 0.133 |  |
|  | grass1 | 0.110±0.045 | 0.080 | 0.128 | n.s. (0.129) |
|  | grass3 | 0.125±0.060 | 0.041 | 0.322 | * (0.047) |
|  | grass4 | 0.102±0.025 | 0.127 | 0.156 | * (0.003) |
| Ic_1 | dicot | 0.071±0.049 | 0.062 | 0.079 |  |
|  | grass1 | 0.105±0.030 | 0.073 | 0.137 | * (0.005) |
|  | grass2 | 0.075±0.020 | 0.063 | 0.086 | * (0.034) |
|  | grass3 | 0.065±0.017 | 0.056 | 0.075 | n.s. (0.380) |
|  | grass4 | 0.110±0.024 | 0.051 | 0.169 | * (0.021) |
|  | grass5 | 0.077±0.016 | 0.063 | 0.092 | * (0.041) |
| Ic_2 | dicot | 0.081±0.030 | 0.070 | 0.091 |  |
|  | grass1 | 0.107±0.040 | 0.085 | 0.129 | * (0.013) |
|  | grass2 | 0.104±0.018 | 0.085 | 0.122 | * (0.016) |
| Ic_3 | dicot | 0.074±0.051 | 0.065 | 0.084 |  |
|  | grass1 | 0.068±0.036 | 0.021 | 0.158 | n.s. (0.993) |
|  | grass2 | 0.072±0.031 | 0.052 | 0.091 | n.s. (0.641) |
| Ic_4 | dicot | 0.047±0.039 | 0.039 | 0.055 |  |
|  | grass1 | 0.051±0.007 | 0.043 | 0.059 | n.s. (0.106) |
|  | grass2 | 0.057±0.022 | 0.043 | 0.071 | * (0.025) |
| Ic_5 | dicot | 0.069±0.029 | 0.059 | 0.079 |  |
|  | grass | 0.062±0.016 | 0.053 | 0.071 | n.s. (0.556) |
| II_1 | dicot | 0.092±0.034 | 0.085 | 0.099 |  |
|  | grass1 | 0.042±0.011 | 0.033 | 0.050 | ** |
|  | grass2 | 0.071±0.019 | 0.052 | 0.091 | n.s. (0.104) |
| II_2 | dicot | 0.187±0.085 | 0.143 | 0.231 |  |
|  | grass1 | 0.321±0.104 | 0.264 | 0.379 | ** |
|  | grass2 | 0.250±0.060 | 0.216 | 0.283 | * (0.011) |
| II_3 | dicot | 0.052±0.188 | 0.045 | 0.059 |  |
|  | grass1 | 0.036±0.016 | 0.025 | 0.047 | n.s. (0.077) |
|  | grass2 | 0.111±0.032 | 0.088 | 0.134 | ** |
|  | grass3 | 0.185±0.083 | 0.097 | 0.272 | ** |
| II_4 | dicot | 0.181±0.070 | 0.153 | 0.208 |  |
|  | grass | 0.222±0.039 | 0.192 | 0.252 | * (0.025) |
| IIIa_1 | dicot | 0.142±0.049 | 0.131 | 0.152 |  |
|  | grass1 | 0.233±0.124 | 0.144 | 0.322 | ** |
|  | grass2 | 0.158±0.033 | 0.134 | 0.181 | n.s. (0.127) |
| IIIa_2 | dicot | 0.182±0.061 | 0.154 | 0.209 |  |
|  | grass | 0.394±0.094 | 0.343 | 0.446 | ** |
| IIIb_1 | dicot | 0.080±0.091 | 0.066 | 0.093 |  |
|  | grass1 | 0.047±0.025 | 0.033 | 0.061 | n.s. (0.537) |
|  | grass2 | 0.182±0.077 | 0.127 | 0.237 | ** |
|  | grass3 | 0.019±0.007 | 0.008 | 0.030 | * (0.019) |
|  | grass4 | 0.063±0.016 | 0.052 | 0.075 | n.s. (0.197) |
|  | grass5 | 0.098±0.030 | 0.077 | 0.120 | * (0.009) |
| IIIb_2 | dicot | 0.200±0.060 | 0.176 | 0.223 |  |
|  | grass1 | 0.273±0.072 | 0.197 | 0.348 | * (0.013) |
|  | grass2 | 0.251±0.037 | 0.231 | 0.272 | * (0.004) |
| IIIb_3 | dicot | 0.224±0.053 | 0.200 | 0.247 |  |
|  | grass | 0.291±0.055 | 0.252 | 0.330 | * (0.003) |
| IIIc | dicot | 0.098±0.039 | 0.082 | 0.113 |  |
|  | grass | 0.104±0.011 | 0.002 | 0.205 | n.s. (0.349) |
| IVb_1 | dicot | 0.130±0.032 | 0.107 | 0.153 |  |
|  | grass1 | 0.112±0.039 | 0.090 | 0.135 | n.s. (0.252) |
|  | grass2 | 0.086±0.028 | 0.064 | 0.108 | * (0.006) |
|  | grass3 | 0.109±0.021 | 0.094 | 0.125 | n.s. (0.064) |
| IVb_2 | dicot | 0.113±0.037 | 0.100 | 0.120 |  |
|  | grass1 | 0.104±0.017 | 0.065 | 0.089 | * (0.037) |
|  | grass2 | 0.078±0.035 | 0.034 | 0.058 | ** |
| IVc | dicot | 0.119±0.071 | 0.109 | 0.128 |  |
|  | grass1 | 0.080±0.008 | 0.075 | 0.085 | ** |
|  | grass2 | 0.130±0.054 | 0.092 | 0.169 | n.s. (0.682) |
|  | grass3 | 0.128±0.017 | 0.119 | 0.137 | n.s. (0.171) |
| IVd_1 | dicot | 0.136±0.055 | 0.122 | 0.151 |  |
|  | grass1 | 0.138±0.033 | 0.119 | 0.156 | n.s. (0.404) |
|  | grass2 | 0.261±0.137 | 0.118 | 0.405 | * (0.003) |
|  | grass3 | 0.143±0.032 | 0.109 | 0.177 | n.s. (0.415) |
| IVd_2 | dicot | 0.091±0.035 | 0.086 | 0.096 |  |
|  | grass1 | 0.096±0.049 | 0.045 | 0.147 | n.s. (0.258) |
|  | grass2 | 0.071±0.016 | 0.058 | 0.083 | n.s. (0.063) |
|  | grass3 | 0.078±0.012 | 0.072 | 0.086 | n.s. (0.221) |
| Va_1 | dicot | 0.146±0.035 | 0.135 | 0.156 |  |
|  | grass1 | 0.096±0.034 | 0.067 | 0.124 | ** |
|  | grass2 | 0.128±0.028 | 0.109 | 0.148 | n.s. (0.257) |
| Va_2 | dicot | 0.133±0.071 | 0.111 | 0.154 |  |
|  | grass | 0.095±0.027 | 0.080 | 0.110 | n.s. (0.069) |
| Vb | dicot | 0.050±0.028 | 0.049 | 0.052 |  |
|  | grass1 | 0.062±0.015 | 0.052 | 0.073 | * (0.049) |
|  | grass2 | 0.029±0.009 | 0.023 | 0.035 | * (0.007) |
|  | grass3 | 0.037±0.006 | 0.032 | 0.042 | n.s. (0.146) |
|  | grass4 | 0.045±0.018 | 0.031 | 0.058 | n.s. (0.731) |
|  | grass5 | 0.060±0.028 | 0.044 | 0.075 | n.s. (0.184) |
|  | grass6 | 0.052±0.027 | 0.038 | 0.065 | n.s. (0.874) |
|  | grass7 | 0.073±0.022 | 0.058 | 0.089 | * (0.004) |
| VIc | dicot | 0.180±0.086 | 0.154 | 0.205 |  |
|  | grass1 | 0.122±0.048 | 0.072 | 0.172 | * (0.012) |
|  | grass2 | 0.196±0.033 | 0.172 | 0.220 | n.s. (0.448) |

^a^: Two-tailed P-value derived from t-test for *ω* value between dicot and grass subgroups, n.s.=not significant, *=significant (P< 5% ) and **= highly significant (P<1% ).
