# Supplementary material for: Divergent Evolutionary Patterns of NAC Transcription Factors Are Associated with Diversification and Gene Duplications in Angiosperm
Source: Front Plant Sci. 2017 Jun 30;8:1156. doi: 10.3389/fpls.2017.01156 (PMC5492850; doi:10.3389/fpls.2017.01156)
Supplement: Supplementary file 2 [file Presentation1.PDF]

Ia_2

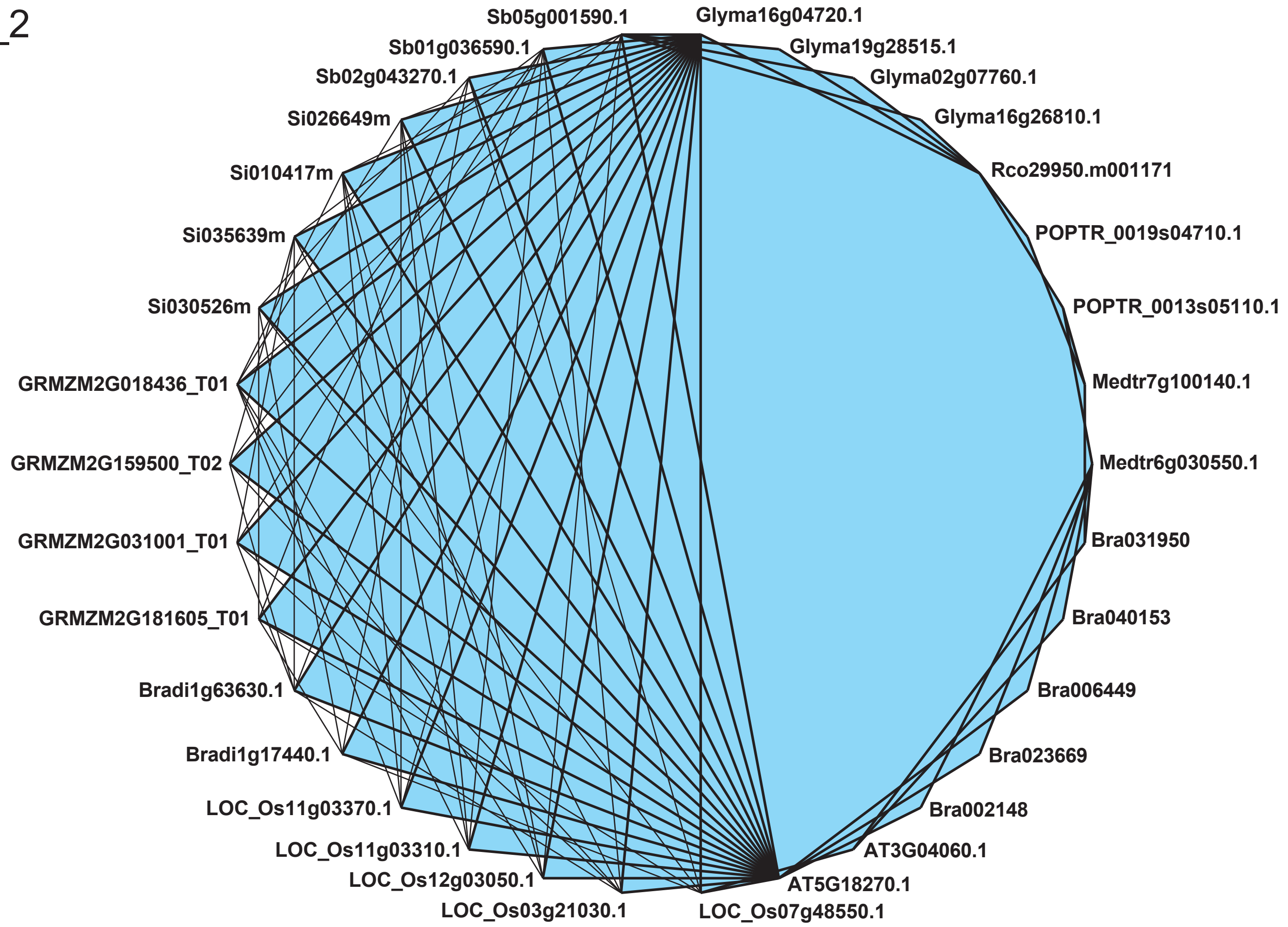
Sb05g001590.1
Glyma16g04720.1
Sb01g036590.1
Glyma19g28515.1
Sb02g043270.1
Glyma02g07760.1
Si026649m
Glyma16g26810.1
Si010417m
Rco29950.m001171
Si035639m
POPTR_0019s04710.1
Si030526m
POPTR_0013s05110.1
GRMZM2G018436_T01
Medtr7g100140.1
GRMZM2G159500_T02
Medtr6g030550.1
GRMZM2G031001_T01
Bra031950
GRMZM2G181605_T01
Bra040153
Bradi1g63630.1
Bra006449
Bradi1g17440.1
Bra023669
LOC_Os11g03370.1
Bra002148
LOC_Os11g03310.1
AT3G04060.1
LOC_Os12g03050.1
AT5G18270.1
LOC_Os03g21030.1
LOC_Os07g48550.1

Ia_3

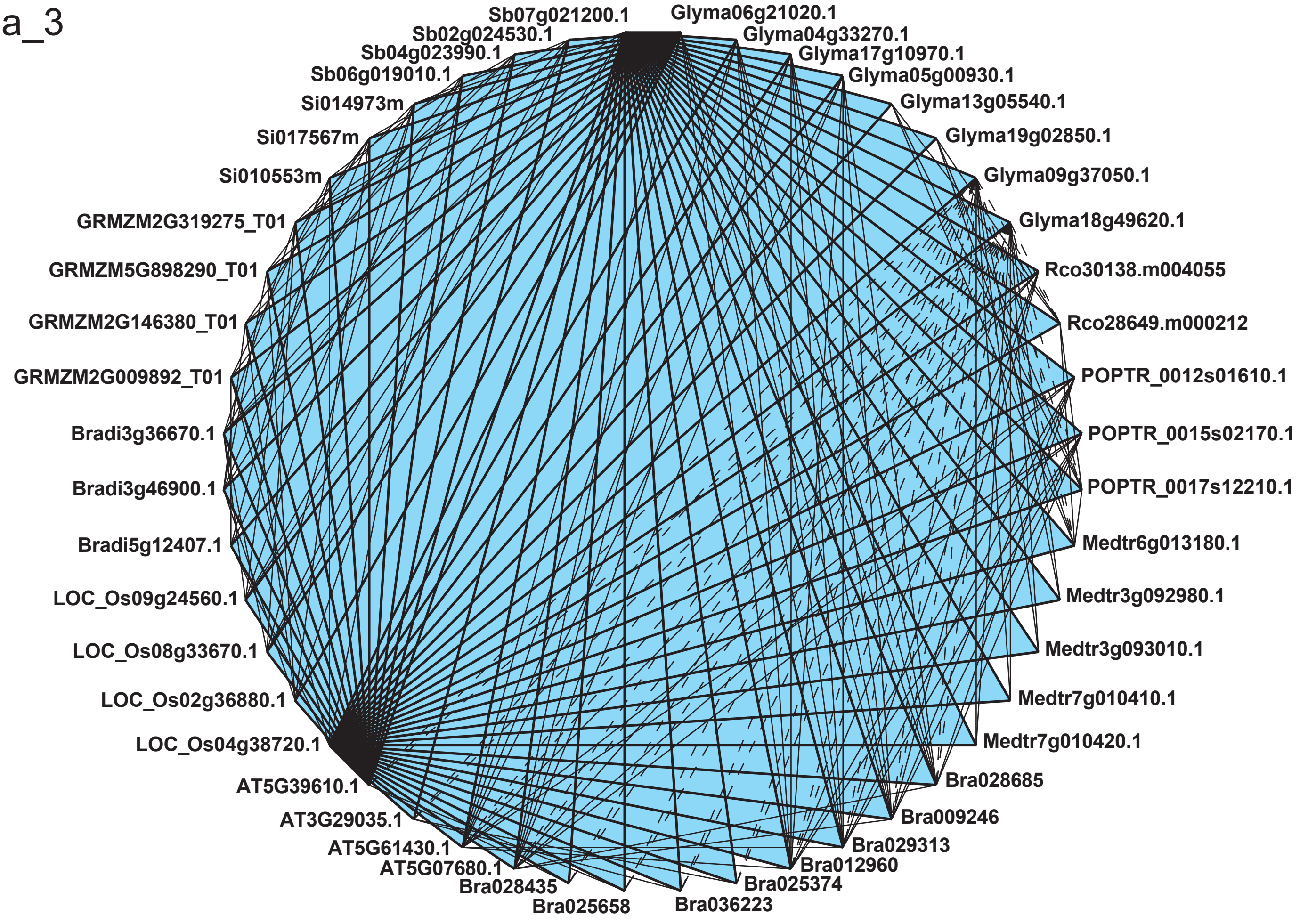
Sb07g021200.1
Glyma06g21020.1
Sb02g024530.1
Glyma04g33270.1
Sb04g023990.1
Glyma17g10970.1
Sb06g019010.1
Glyma05g00930.1
Si014973m
Glyma13g05540.1
Si017567m
Glyma19g02850.1
Si010553m
Glyma09g37050.1
GRMZM2G319275_T01
Glyma18g49620.1
GRMZM5G898290_T01
Rco30138.m004055
GRMZM2G146380_T01
Rco28649.m000212
GRMZM2G009892_T01
POPTR_0012s01610.1
Bradi3g36670.1
POPTR_0015s02170.1
Bradi3g46900.1
POPTR_0017s12210.1
Bradi5g12407.1
Medtr6g013180.1
LOC_Os09g24560.1
Medtr3g092980.1
LOC_Os08g33670.1
Medtr3g093010.1
LOC_Os02g36880.1
Medtr7g010410.1
LOC_Os04g38720.1
Medtr7g010420.1
AT5G39610.1
Bra028685
AT3G29035.1
Bra009246
AT5G61430.1
Bra029313
AT5G07680.1
Bra012960
Bra028435
Bra025374
Bra025658
Bra036223

Ia_5

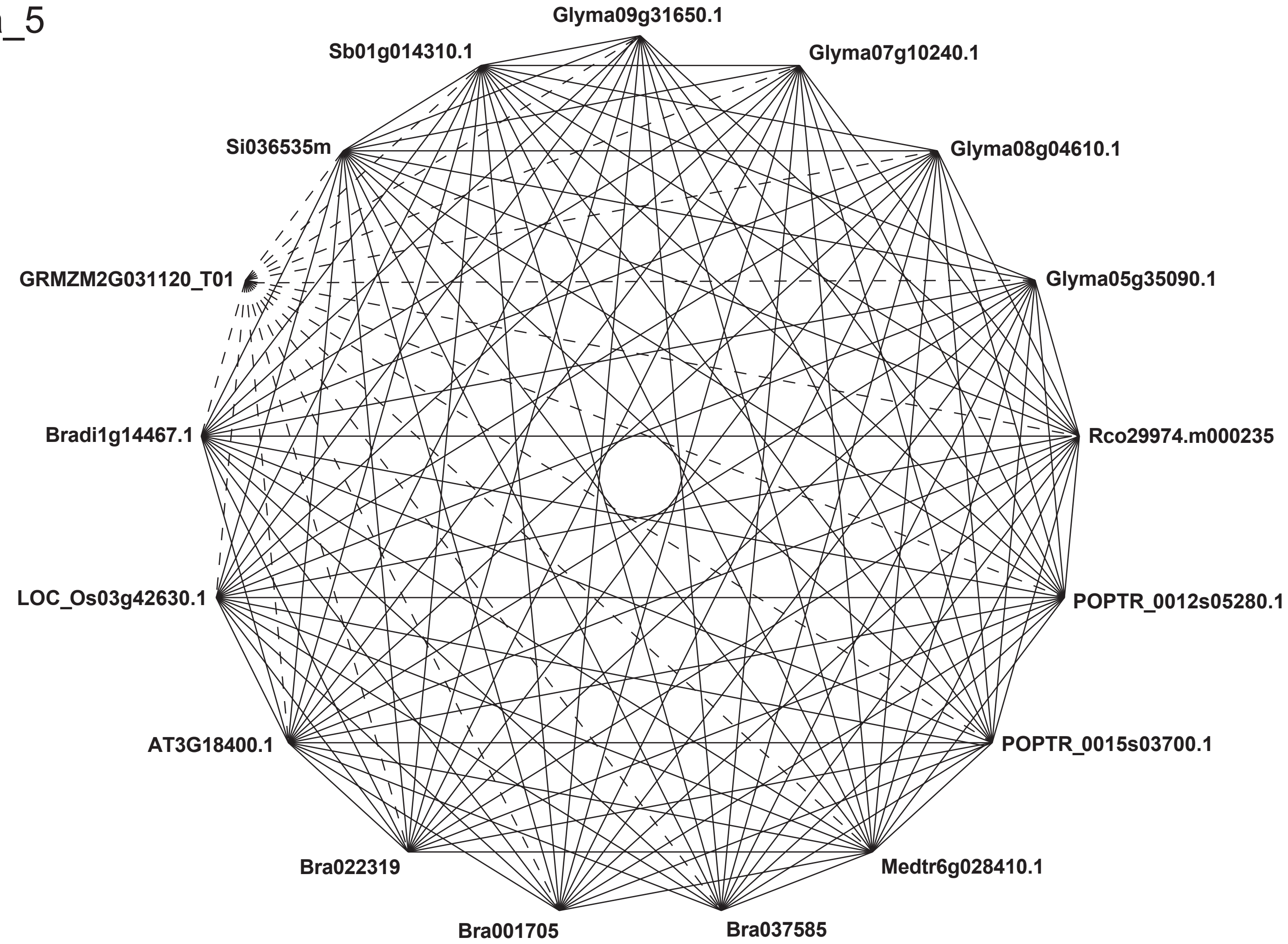
Glyma09g31650.1
Sb01g014310.1
Glyma07g10240.1
Si036535m
Glyma08g04610.1
GRMZM2G031120_T01
Glyma05g35090.1
Bradi1g14467.1
Rco29974.m000235
LOC_Os03g42630.1
POPTR_0012s05280.1
AT3G18400.1
POPTR_0015s03700.1
Bra022319
Medtr6g028410.1
Bra001705
Bra037585

Ib_1

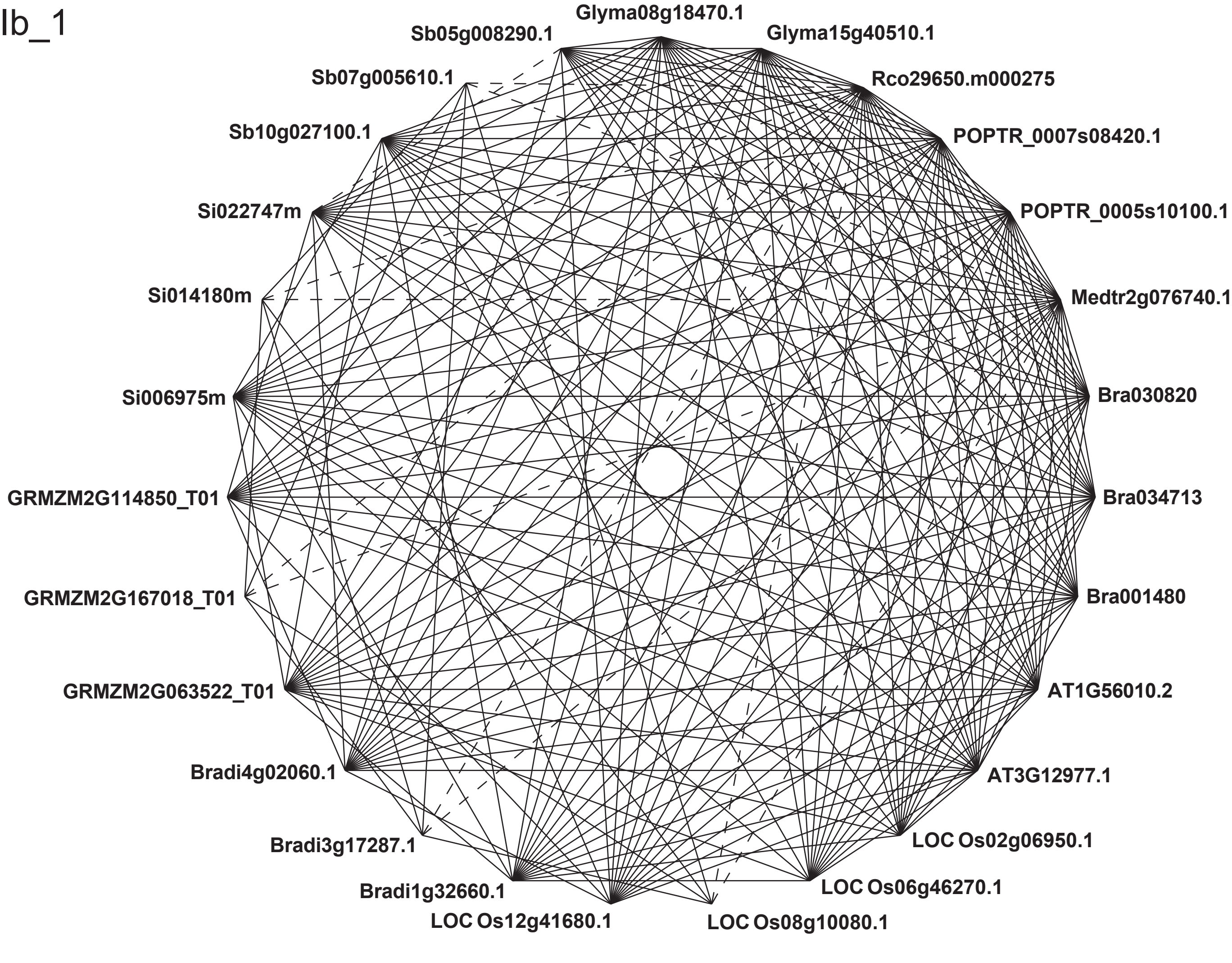
Glyma08g18470.1
Sb05g008290.1
Glyma15g40510.1
Sb07g005610.1
Rco29650.m000275
Sb10g027100.1
POPTR_0007s08420.1
Si022747m
POPTR_0005s10100.1
Si014180m
Medtr2g076740.1
Si006975m
Bra030820
GRMZM2G114850_T01
Bra034713
GRMZM2G167018_T01
Bra001480
GRMZM2G063522_T01
AT1G56010.2
Bradi4g02060.1
AT3G12977.1
Bradi3g17287.1
LOC Os02g06950.1
Bradi1g32660.1
LOC Os06g46270.1
LOC Os12g41680.1
LOC Os08g10080.1

Ib_2

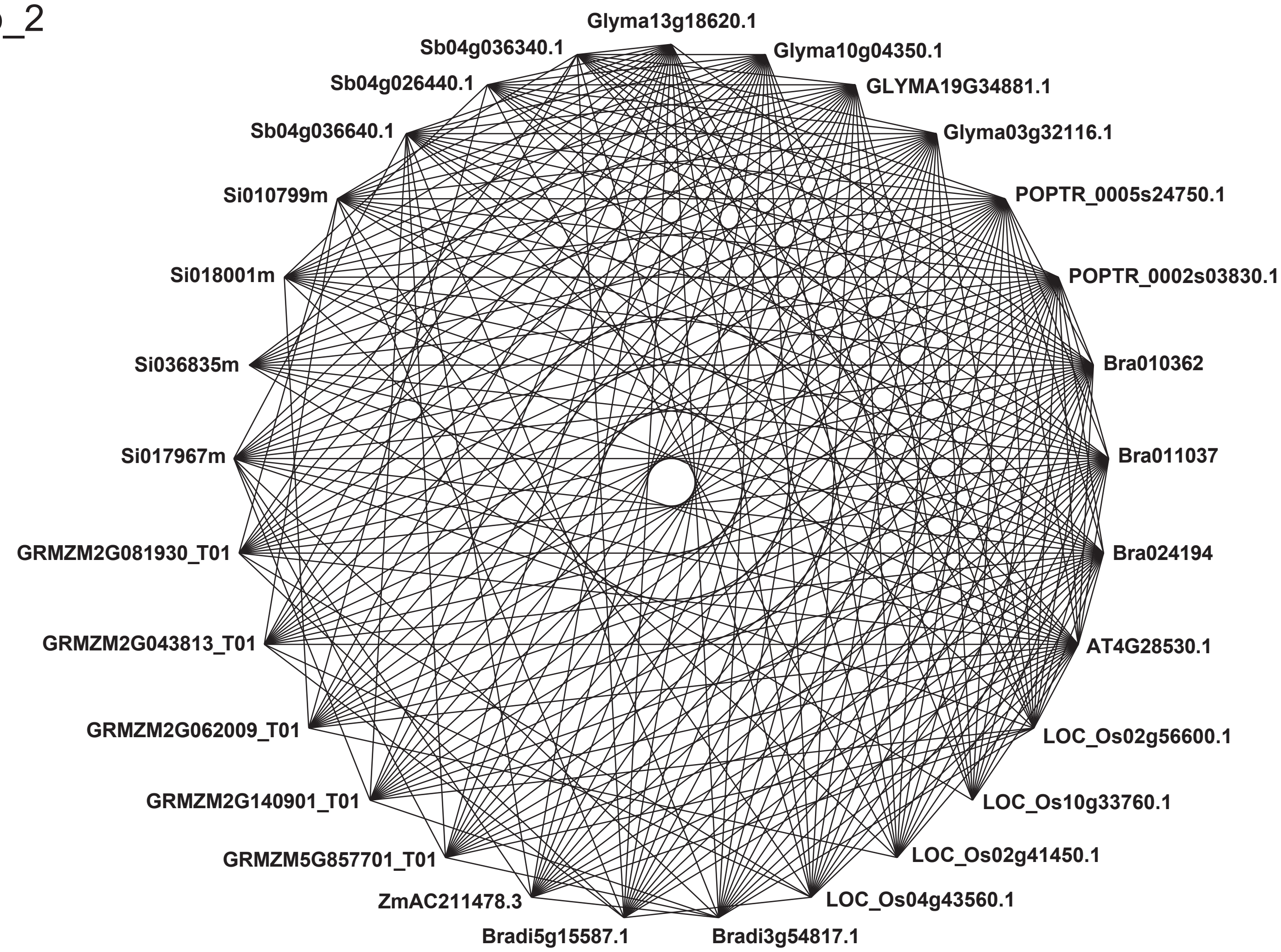
Glyma13g18620.1
Glyma10g04350.1
GLYMA19G34881.1
Glyma03g32116.1
POPTR_0005s24750.1
POPTR_0002s03830.1
Bra010362
Bra011037
Bra024194
AT4G28530.1
LOC_Os02g56600.1
LOC_Os10g33760.1
LOC_Os02g41450.1
LOC_Os04g43560.1
Bradi3g54817.1
Bradi5g15587.1
ZmAC211478.3
GRMZM5G857701_T01
GRMZM2G140901_T01
GRMZM2G062009_T01
GRMZM2G043813_T01
GRMZM2G081930_T01
Si017967m
Si036835m
Si018001m
Si010799m
Sb04g036640.1
Sb04g026440.1
Sb04g036340.1

Ic_2

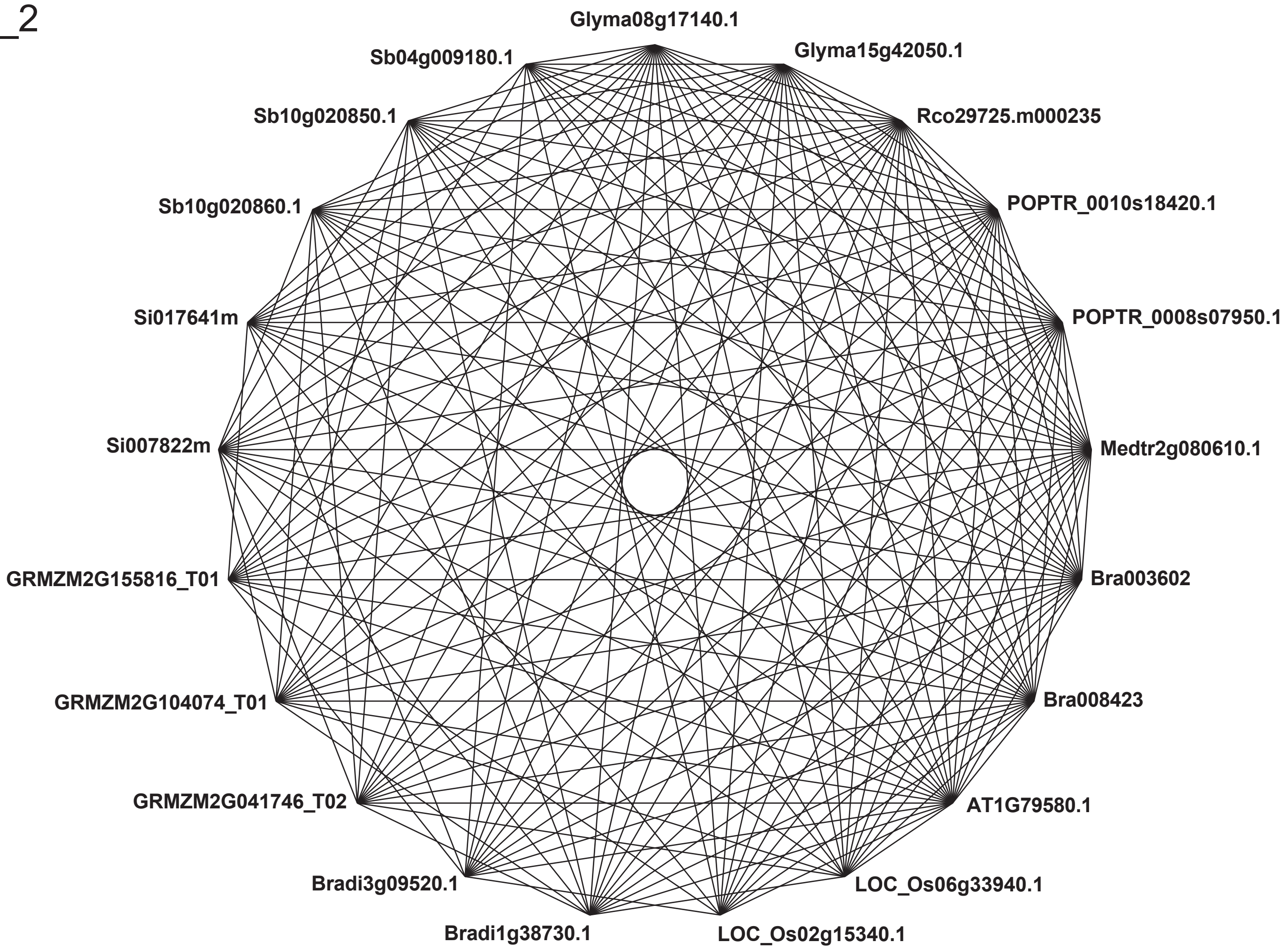
Glyma08g17140.1
Glyma15g42050.1
Rco29725.m000235
POPTR_0010s18420.1
POPTR_0008s07950.1
Medtr2g080610.1
Bra003602
Bra008423
AT1G79580.1
LOC_Os06g33940.1
LOC_Os02g15340.1
Bradi1g38730.1
Bradi3g09520.1
GRMZM2G041746_T02
GRMZM2G104074_T01
GRMZM2G155816_T01
Si007822m
Si017641m
Sb10g020860.1
Sb10g020850.1
Sb04g009180.1

Ic_3

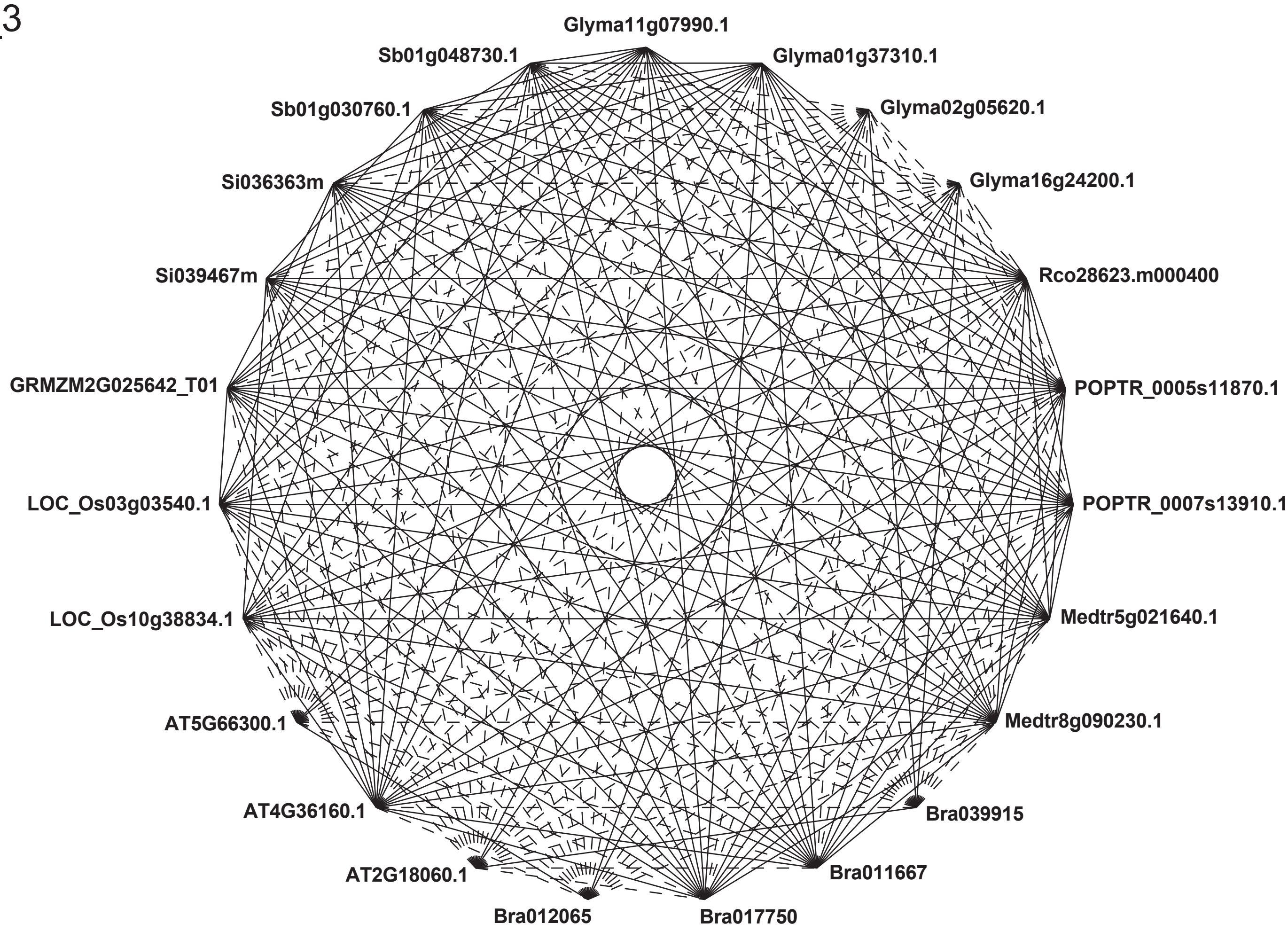
Glyma11g07990.1
Sb01g048730.1
Glyma01g37310.1
Sb01g030760.1
Glyma02g05620.1
Si036363m
Glyma16g24200.1
Si039467m
Rco28623.m000400
GRMZM2G025642_T01
POPTR_0005s11870.1
LOC_Os03g03540.1
POPTR_0007s13910.1
LOC_Os10g38834.1
Medtr5g021640.1
AT5G66300.1
Medtr8g090230.1
AT4G36160.1
Bra039915
AT2G18060.1
Bra011667
Bra012065
Bra017750

Ic_4

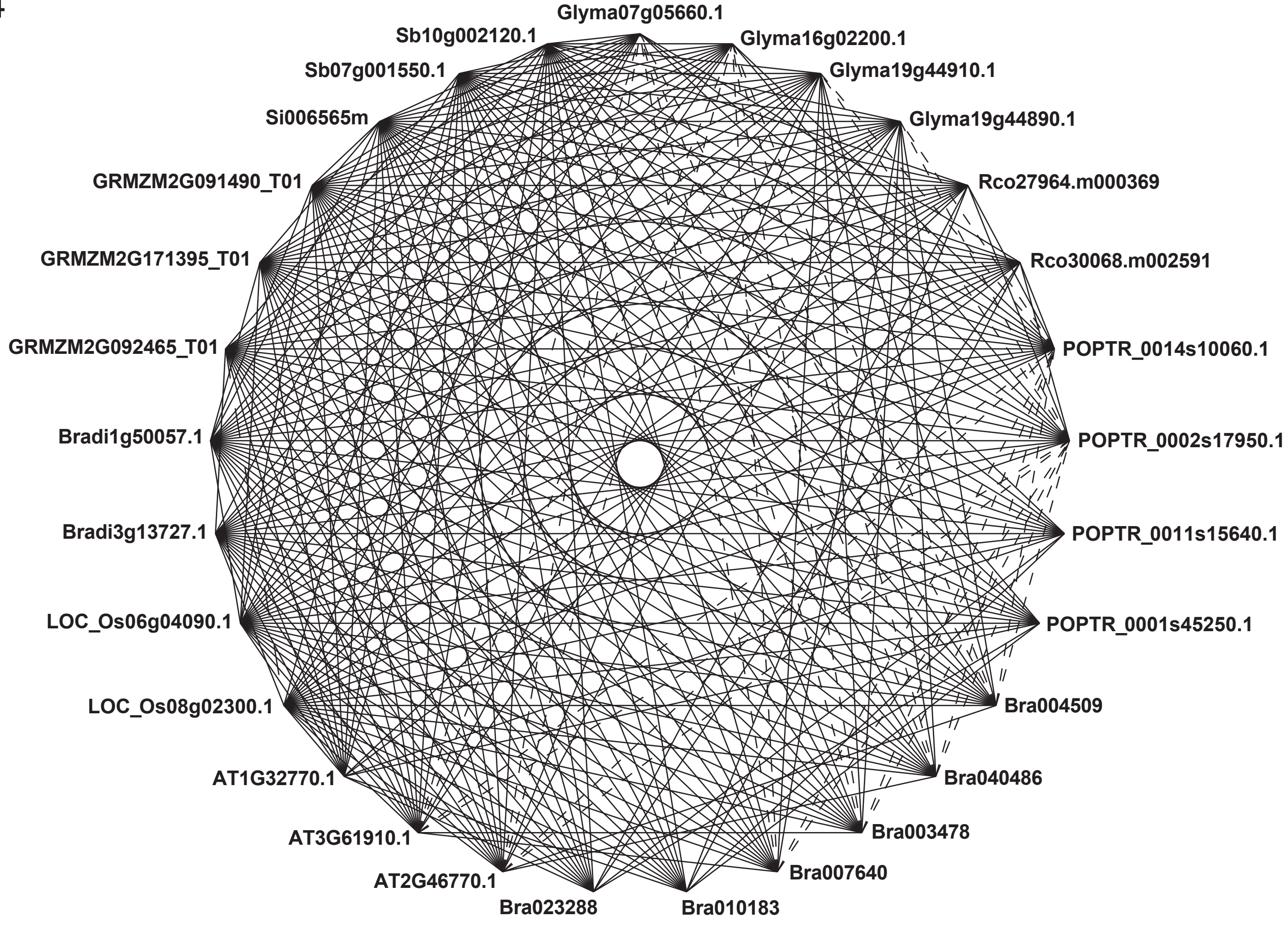
Glyma07g05660.1
Sb10g002120.1
Glyma16g02200.1
Sb07g001550.1
Glyma19g44910.1
Si006565m
Glyma19g44890.1
GRMZM2G091490_T01
Rco27964.m000369
GRMZM2G171395_T01
Rco30068.m002591
GRMZM2G092465_T01
POPTR_0014s10060.1
Bradi1g50057.1
POPTR_0002s17950.1
Bradi3g13727.1
POPTR_0011s15640.1
LOC_Os06g04090.1
POPTR_0001s45250.1
LOC_Os08g02300.1
Bra004509
AT1G32770.1
Bra040486
AT3G61910.1
Bra003478
AT2G46770.1
Bra007640
Bra023288
Bra010183

Ic_5

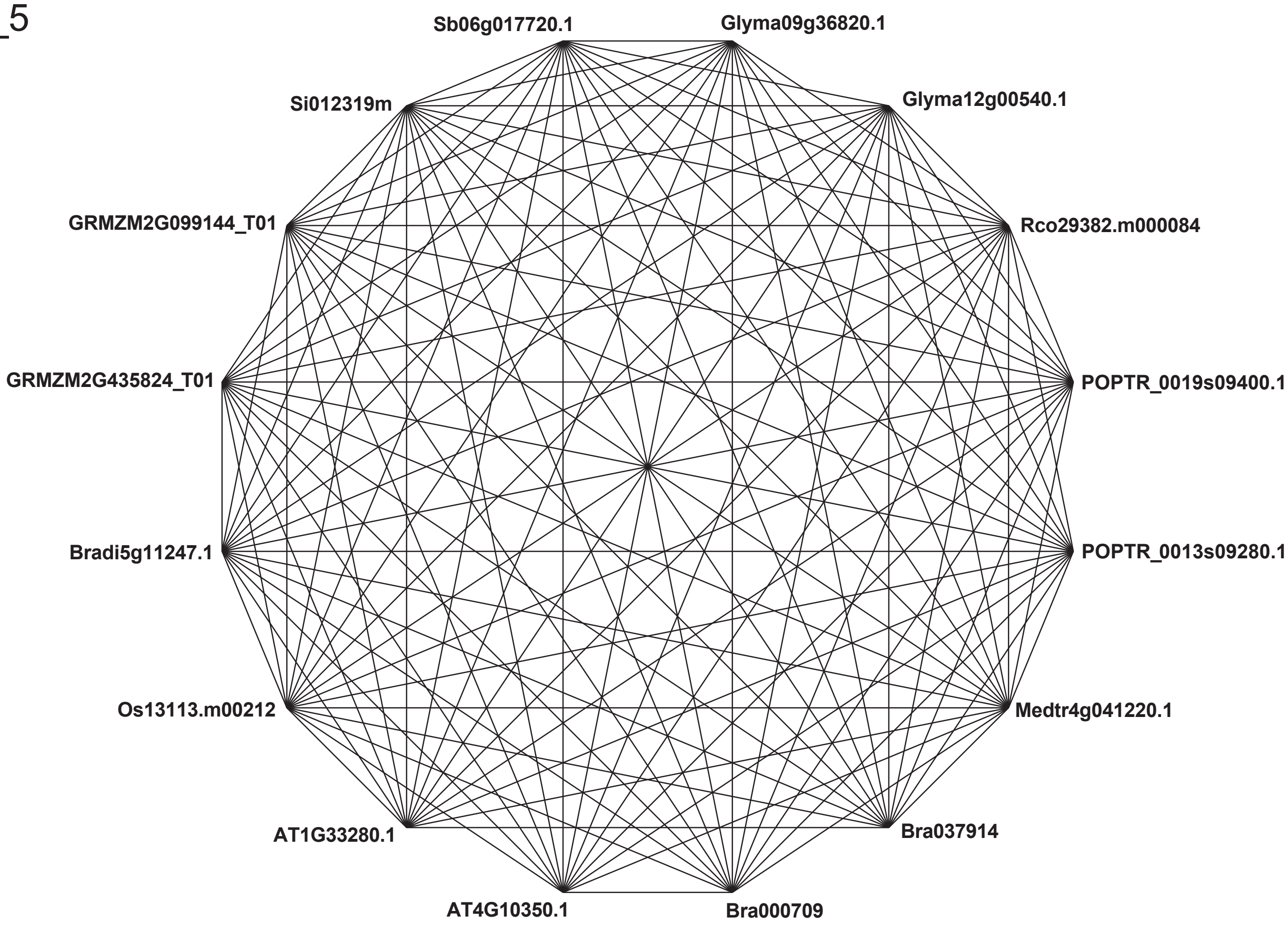
Sb06g017720.1
Glyma09g36820.1
Si012319m
Glyma12g00540.1
GRMZM2G099144_T01
Rco29382.m000084
GRMZM2G435824_T01
POPTR_0019s09400.1
Bradi5g11247.1
POPTR_0013s09280.1
Os13113.m00212
Medtr4g041220.1
AT1G33280.1
Bra037914
AT4G10350.1
Bra000709

II_1

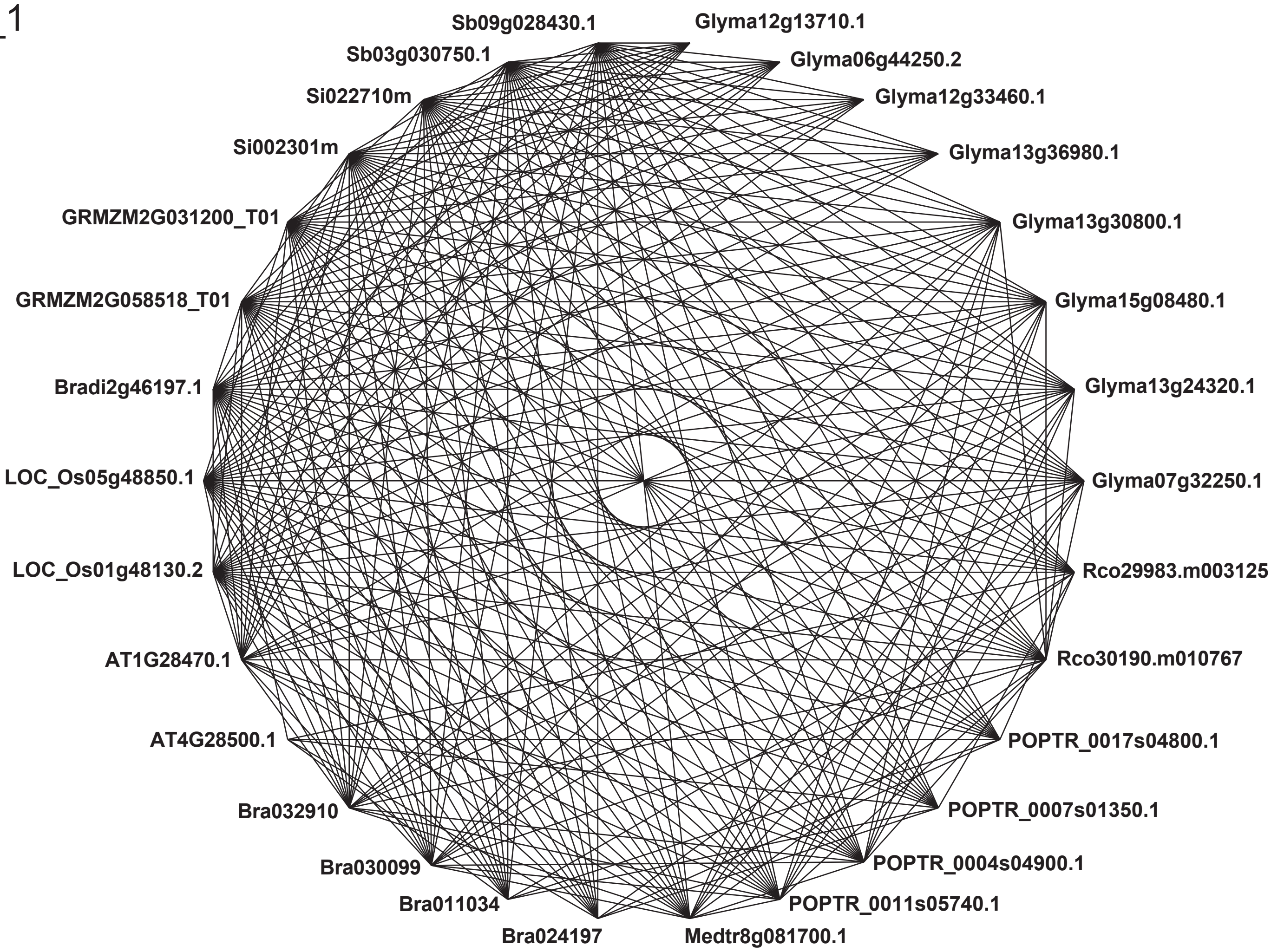
Sb09g028430.1
Glyma12g13710.1
Sb03g030750.1
Glyma06g44250.2
Si022710m
Glyma12g33460.1
Si002301m
Glyma13g36980.1
GRMZM2G031200_T01
Glyma13g30800.1
GRMZM2G058518_T01
Glyma15g08480.1
Bradi2g46197.1
Glyma13g24320.1
LOC_Os05g48850.1
Glyma07g32250.1
LOC_Os01g48130.2
Rco29983.m003125
AT1G28470.1
Rco30190.m010767
AT4G28500.1
POPTR_0017s04800.1
Bra032910
POPTR_0007s01350.1
Bra030099
POPTR_0004s04900.1
Bra011034
POPTR_0011s05740.1
Bra024197
Medtr8g081700.1

II_2

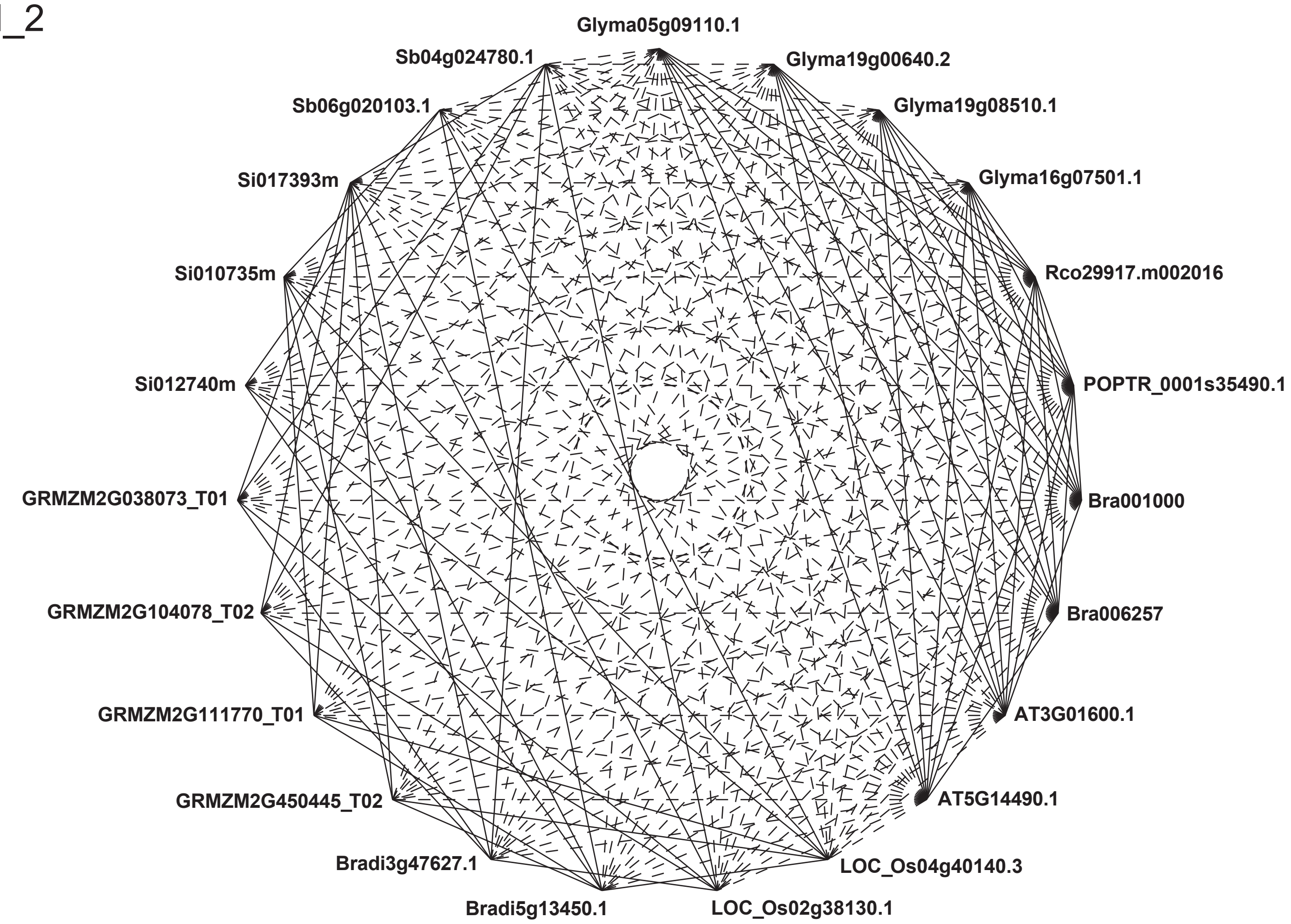
Glyma05g09110.1
Sb04g024780.1
Glyma19g00640.2
Sb06g020103.1
Glyma19g08510.1
Si017393m
Glyma16g07501.1
Si010735m
Rco29917.m002016
Si012740m
POPTR_0001s35490.1
GRMZM2G038073_T01
Bra001000
GRMZM2G104078_T02
Bra006257
GRMZM2G111770_T01
AT3G01600.1
GRMZM2G450445_T02
AT5G14490.1
Bradi3g47627.1
LOC_Os04g40140.3
Bradi5g13450.1
LOC_Os02g38130.1

II_3

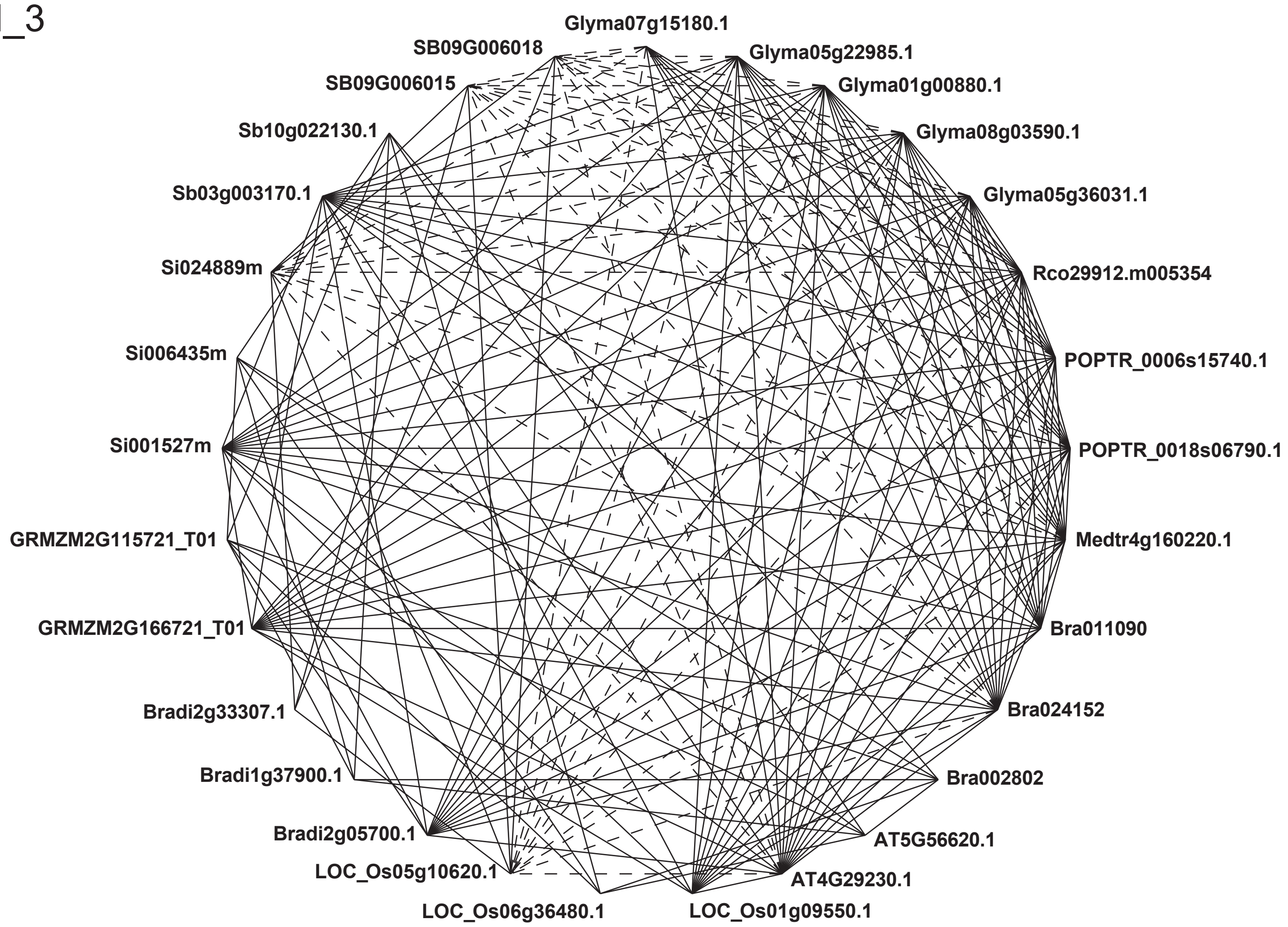
Glyma07g15180.1
SB09G006018
Glyma05g22985.1
SB09G006015
Glyma01g00880.1
Sb10g022130.1
Glyma08g03590.1
Sb03g003170.1
Glyma05g36031.1
Si024889m
Rco29912.m005354
Si006435m
POPTR_0006s15740.1
Si001527m
POPTR_0018s06790.1
GRMZM2G115721_T01
Medtr4g160220.1
GRMZM2G166721_T01
Bra011090
Bradi2g33307.1
Bra024152
Bradi1g37900.1
Bra002802
Bradi2g05700.1
AT5G56620.1
LOC_Os05g10620.1
AT4G29230.1
LOC_Os06g36480.1
LOC_Os01g09550.1

II_4

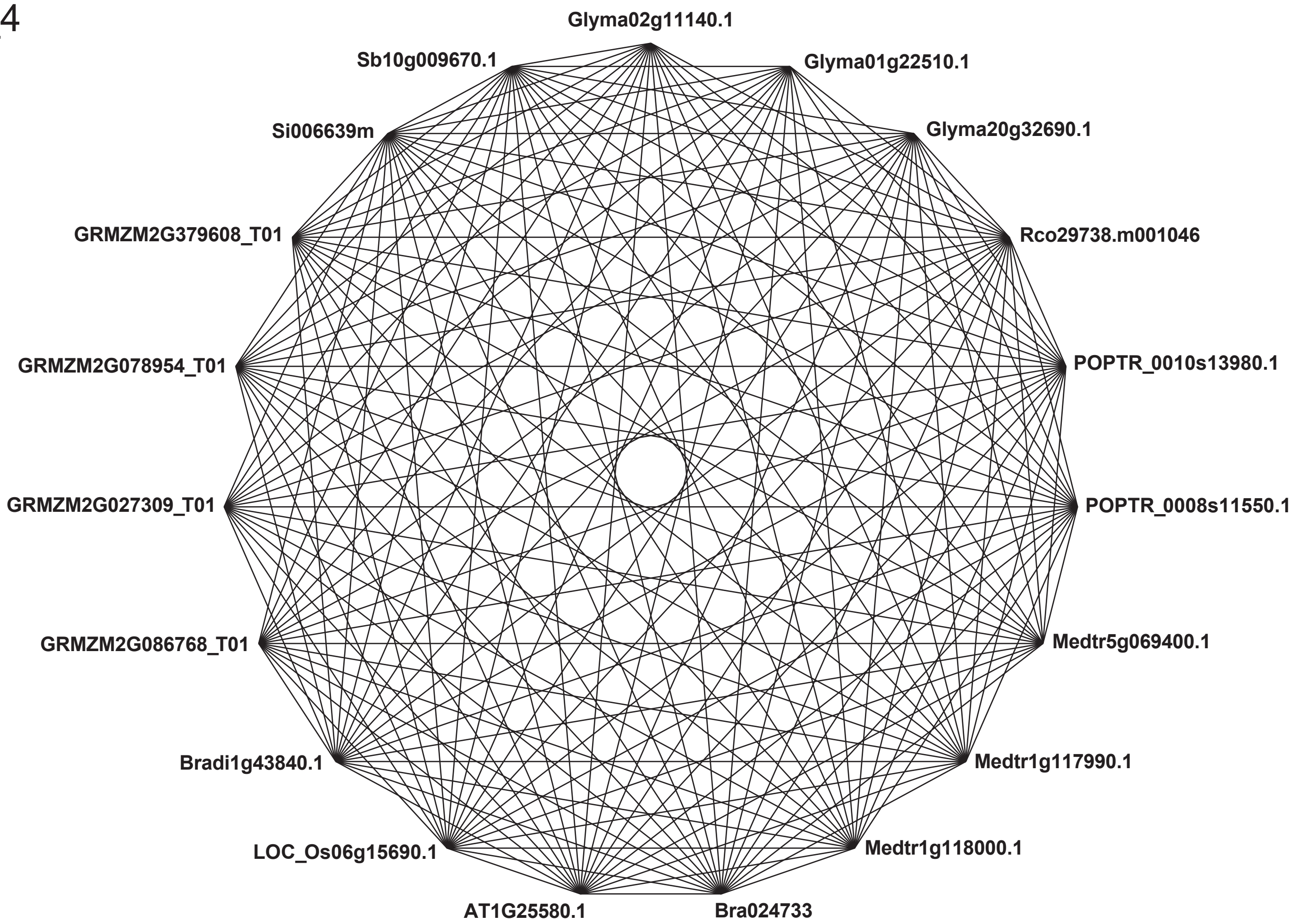
Glyma02g11140.1
Sb10g009670.1
Glyma01g22510.1
Si006639m
Glyma20g32690.1
GRMZM2G379608_T01
Rco29738.m001046
GRMZM2G078954_T01
POPTR_0010s13980.1
GRMZM2G027309_T01
POPTR_0008s11550.1
GRMZM2G086768_T01
Medtr5g069400.1
Bradi1g43840.1
Medtr1g117990.1
LOC_Os06g15690.1
Medtr1g118000.1
AT1G25580.1
Bra024733

IIIa_1

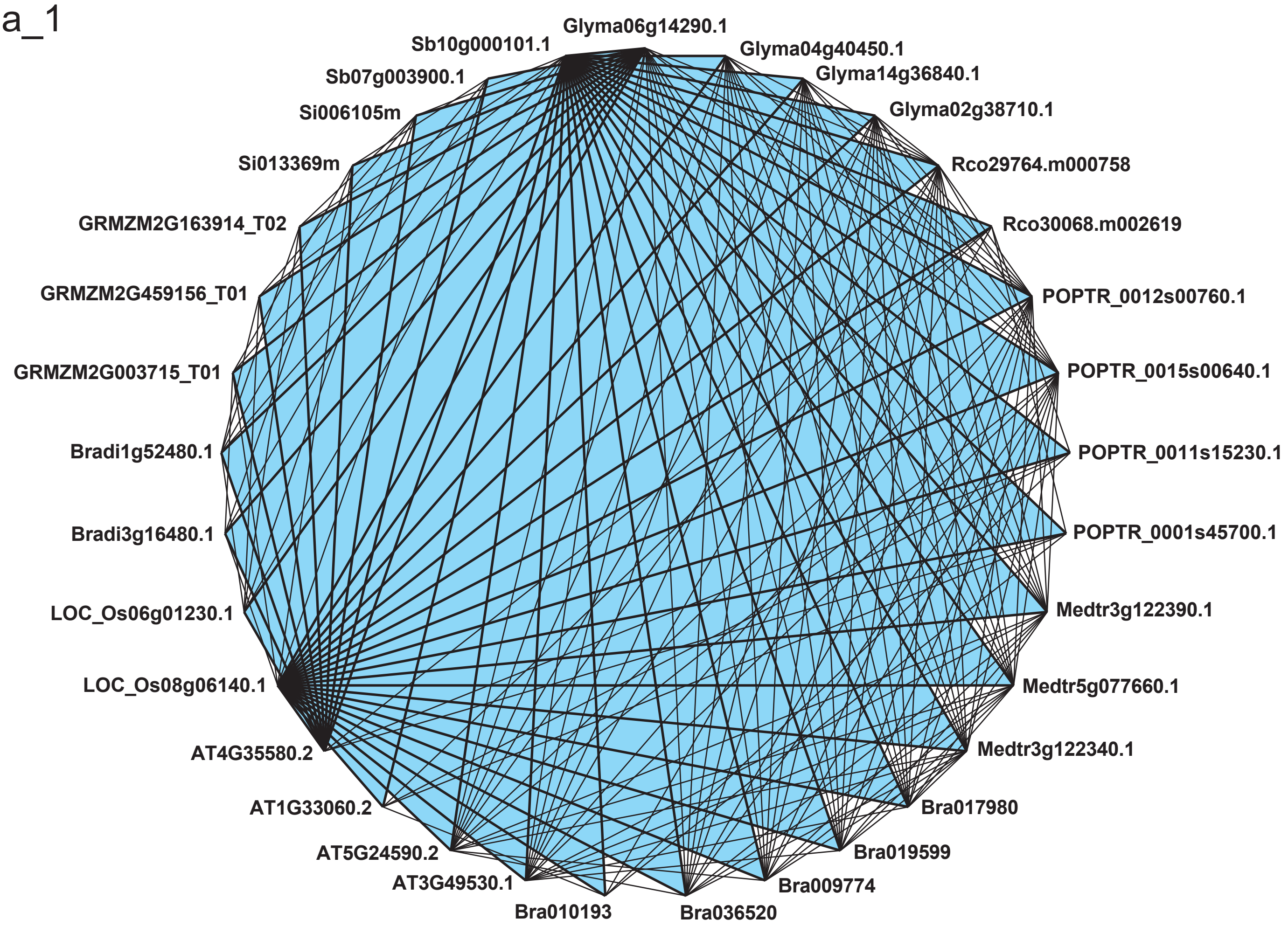
Glyma06g14290.1
Sb10g000101.1
Glyma04g40450.1
Sb07g003900.1
Glyma14g36840.1
Si006105m
Glyma02g38710.1
Si013369m
Rco29764.m000758
GRMZM2G163914_T02
Rco30068.m002619
GRMZM2G459156_T01
POPTR_0012s00760.1
GRMZM2G003715_T01
POPTR_0015s00640.1
Bradi1g52480.1
POPTR_0011s15230.1
Bradi3g16480.1
POPTR_0001s45700.1
LOC_Os06g01230.1
Medtr3g122390.1
LOC_Os08g06140.1
Medtr5g077660.1
AT4G35580.2
Medtr3g122340.1
AT1G33060.2
Bra017980
AT5G24590.2
Bra019599
AT3G49530.1
Bra009774
Bra010193
Bra036520

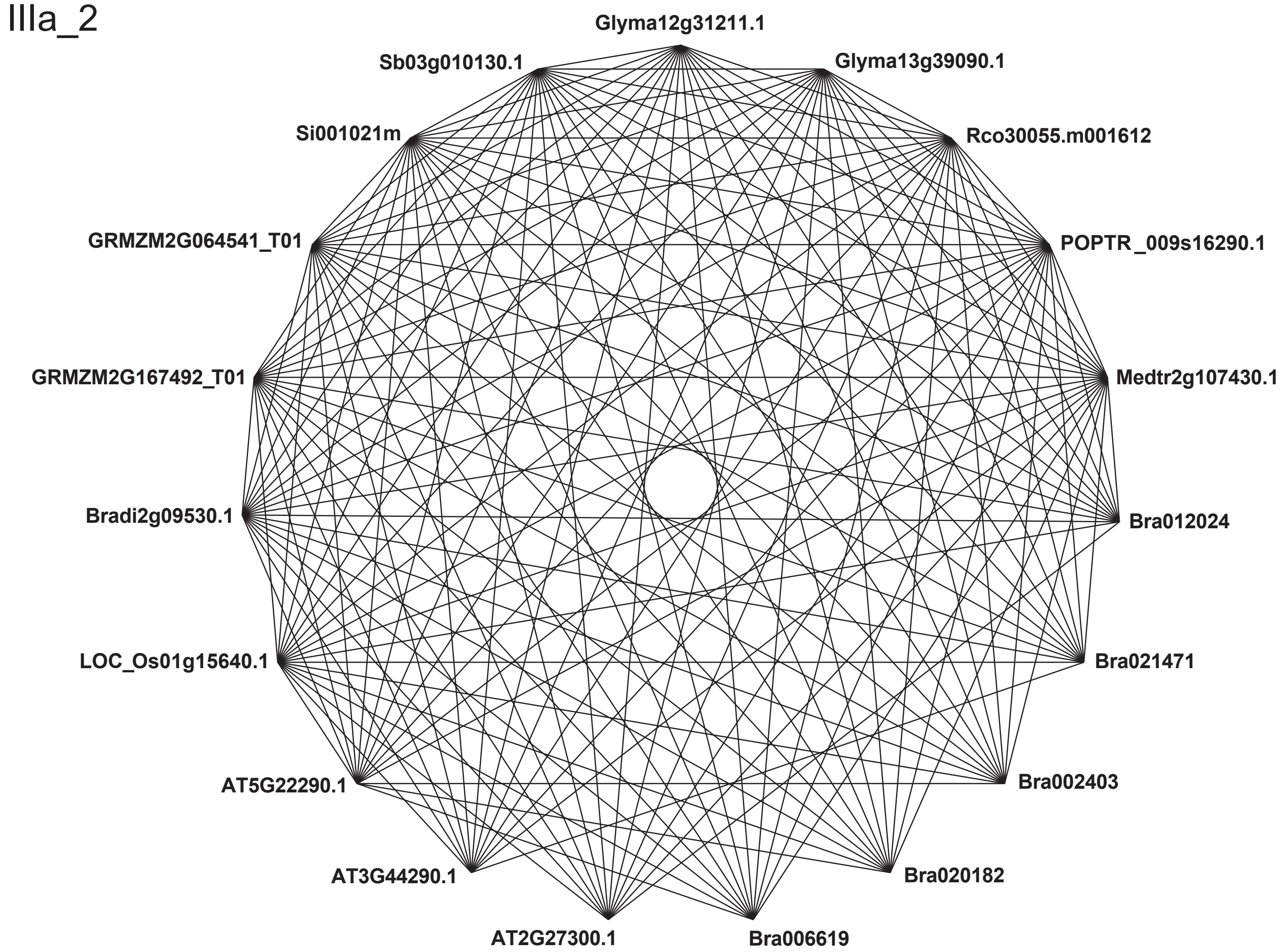
IIIa_2
Glyma12g31211.1
Sb03g010130.1
Glyma13g39090.1
Si001021m
Rco30055.m001612
GRMZM2G064541_T01
POPTR _009s16290.1
GRMZM2G167492_T01
Medtr2g107430.1
Bradi2g09530.1
Bra012024
LOC_Os01g15640.1
Bra021471
AT5G22290.1
Bra002403
AT3G44290.1
Bra020182
AT2G27300.1
Bra006619

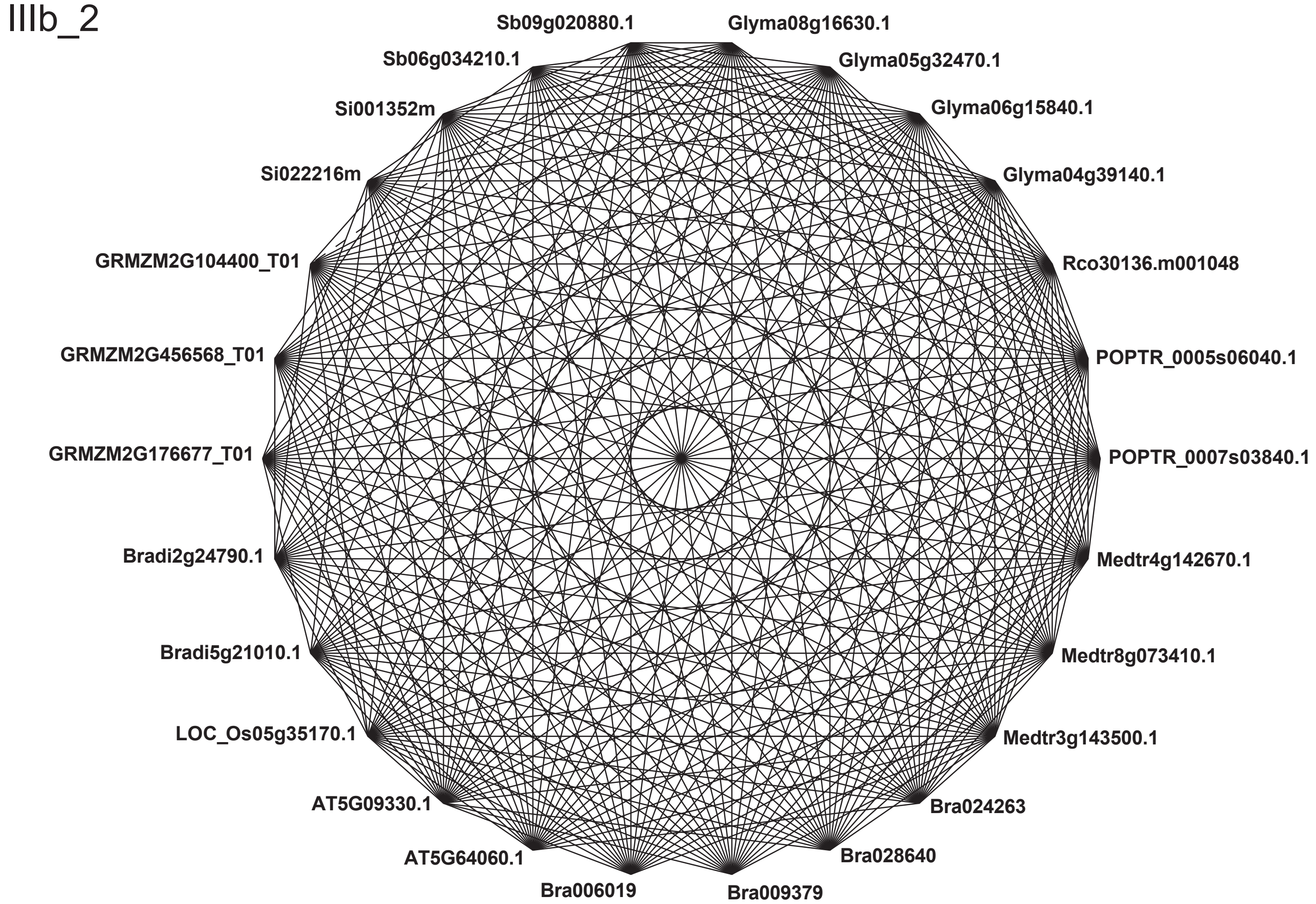
IIIb_2
Sb09g020880.1
Glyma08g16630.1
Sb06g034210.1
Glyma05g32470.1
Si001352m
Glyma06g15840.1
Si022216m
Glyma04g39140.1
GRMZM2G104400_T01
Rco30136.m001048
GRMZM2G456568_T01
POPTR_0005s06040.1
GRMZM2G176677_T01
POPTR_0007s03840.1
Bradi2g24790.1
Medtr4g142670.1
Bradi5g21010.1
Medtr8g073410.1
LOC_Os05g35170.1
Medtr3g143500.1
AT5G09330.1
Bra024263
AT5G64060.1
Bra028640
Bra006019
Bra009379

IIIb_3

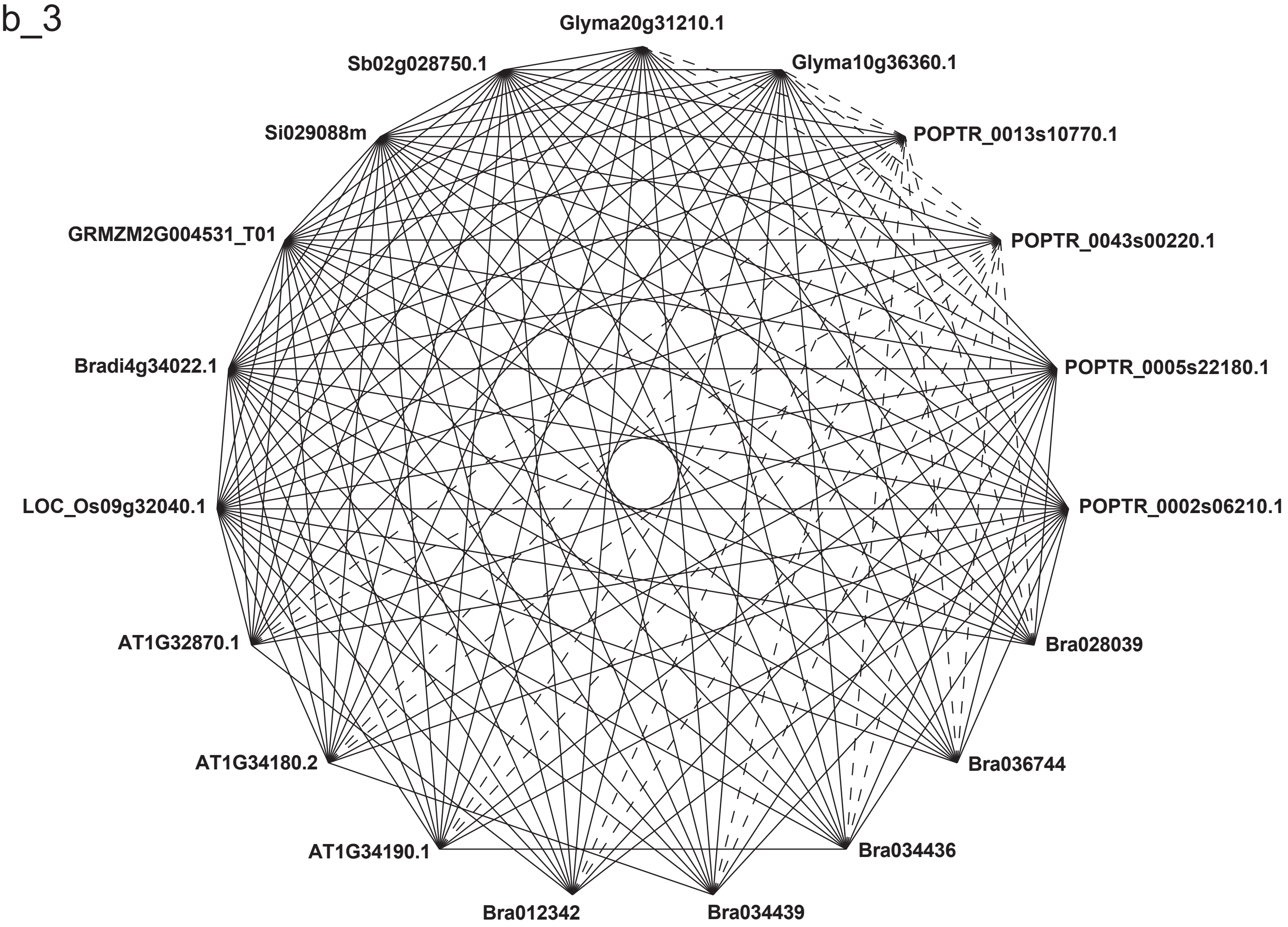

Glyma20g31210.1
Glyma10g36360.1
POPTR_0013s10770.1
POPTR_0043s00220.1
POPTR_0005s22180.1
POPTR_0002s06210.1
Bra028039
Bra036744
Bra034436
Bra034439
Bra012342
AT1G34190.1
AT1G34180.2
AT1G32870.1
LOC_Os09g32040.1
Bradi4g34022.1
GRMZM2G004531_T01
Si029088m
Sb02g028750.1

IIIc

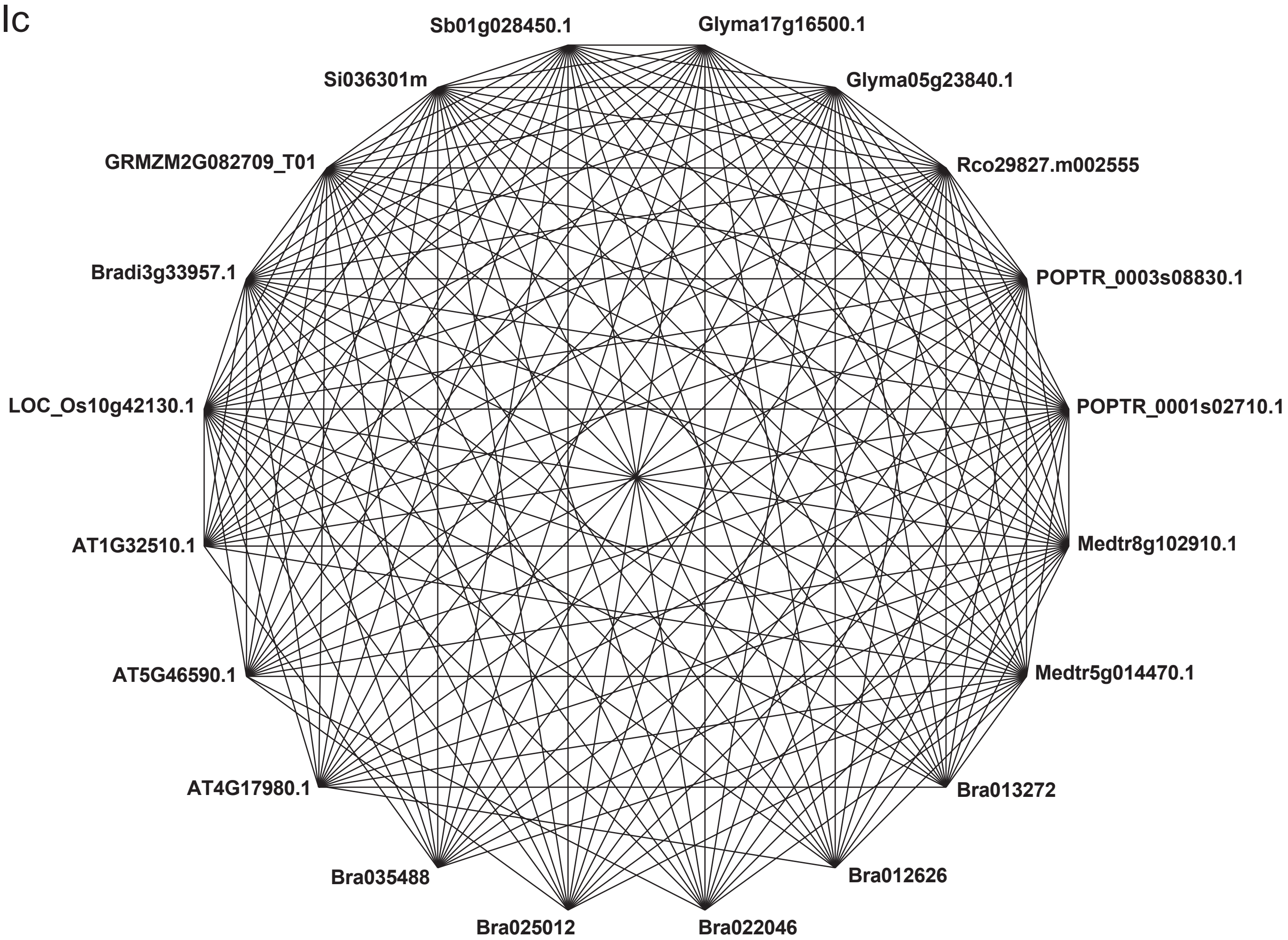

Sb01g028450.1
Glyma17g16500.1
Glyma05g23840.1
Rco29827.m002555
POPTR_0003s08830.1
POPTR_0001s02710.1
Medtr8g102910.1
Medtr5g014470.1
Bra013272
Bra012626
Bra022046
Bra025012
Bra035488
AT4G17980.1
AT5G46590.1
AT1G32510.1
LOC_Os10g42130.1
Bradi3g33957.1
GRMZM2G082709_T01
Si036301m

IVb_1

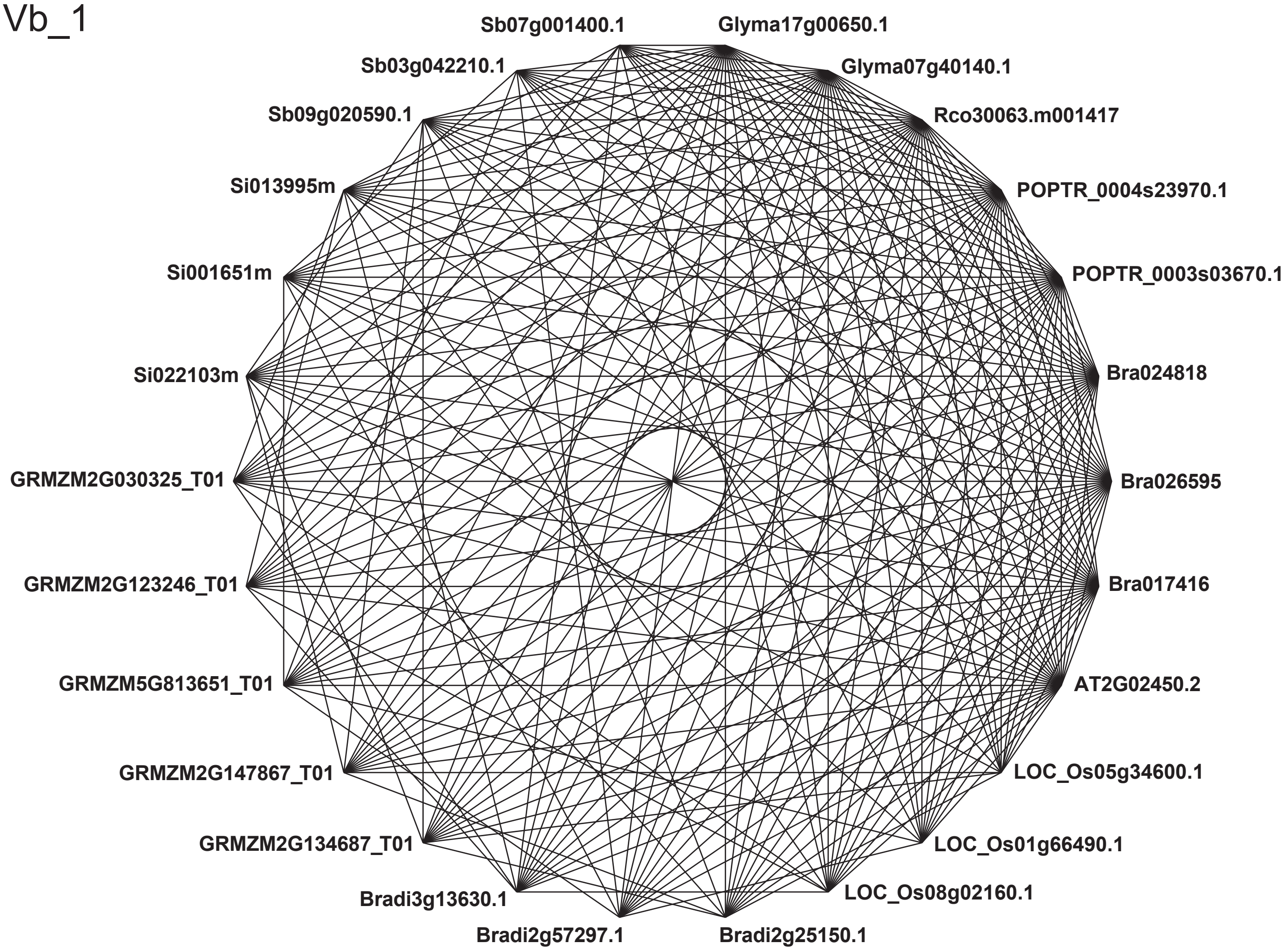
Sb07g001400.1
Glyma17g00650.1
Sb03g042210.1
Glyma07g40140.1
Sb09g020590.1
Rco30063.m001417
Si013995m
POPTR_0004s23970.1
Si001651m
POPTR_0003s03670.1
Si022103m
Bra024818
GRMZM2G030325_T01
Bra026595
GRMZM2G123246_T01
Bra017416
GRMZM5G813651_T01
AT2G02450.2
GRMZM2G147867_T01
LOC_Os05g34600.1
GRMZM2G134687_T01
LOC_Os01g66490.1
Bradi3g13630.1
LOC_Os08g02160.1
Bradi2g57297.1
Bradi2g25150.1

IVb_2

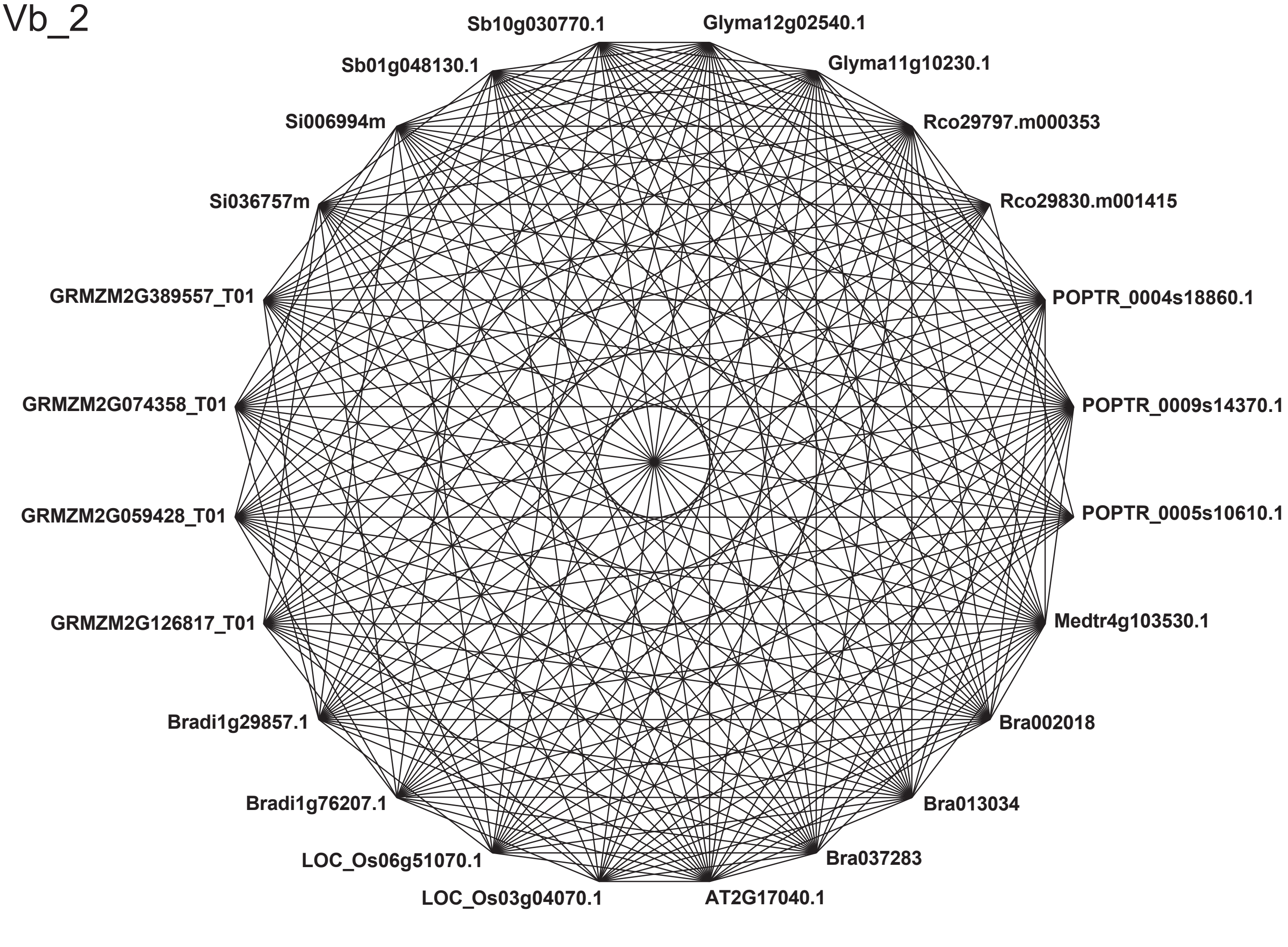
Sb10g030770.1
Glyma12g02540.1
Sb01g048130.1
Glyma11g10230.1
Si006994m
Rco29797.m000353
Si036757m
Rco29830.m001415
GRMZM2G389557_T01
POPTR_0004s18860.1
GRMZM2G074358_T01
POPTR_0009s14370.1
GRMZM2G059428_T01
POPTR_0005s10610.1
GRMZM2G126817_T01
Medtr4g103530.1
Bradi1g29857.1
Bra002018
Bradi1g76207.1
Bra013034
LOC_Os06g51070.1
Bra037283
LOC_Os03g04070.1
AT2G17040.1

IVc

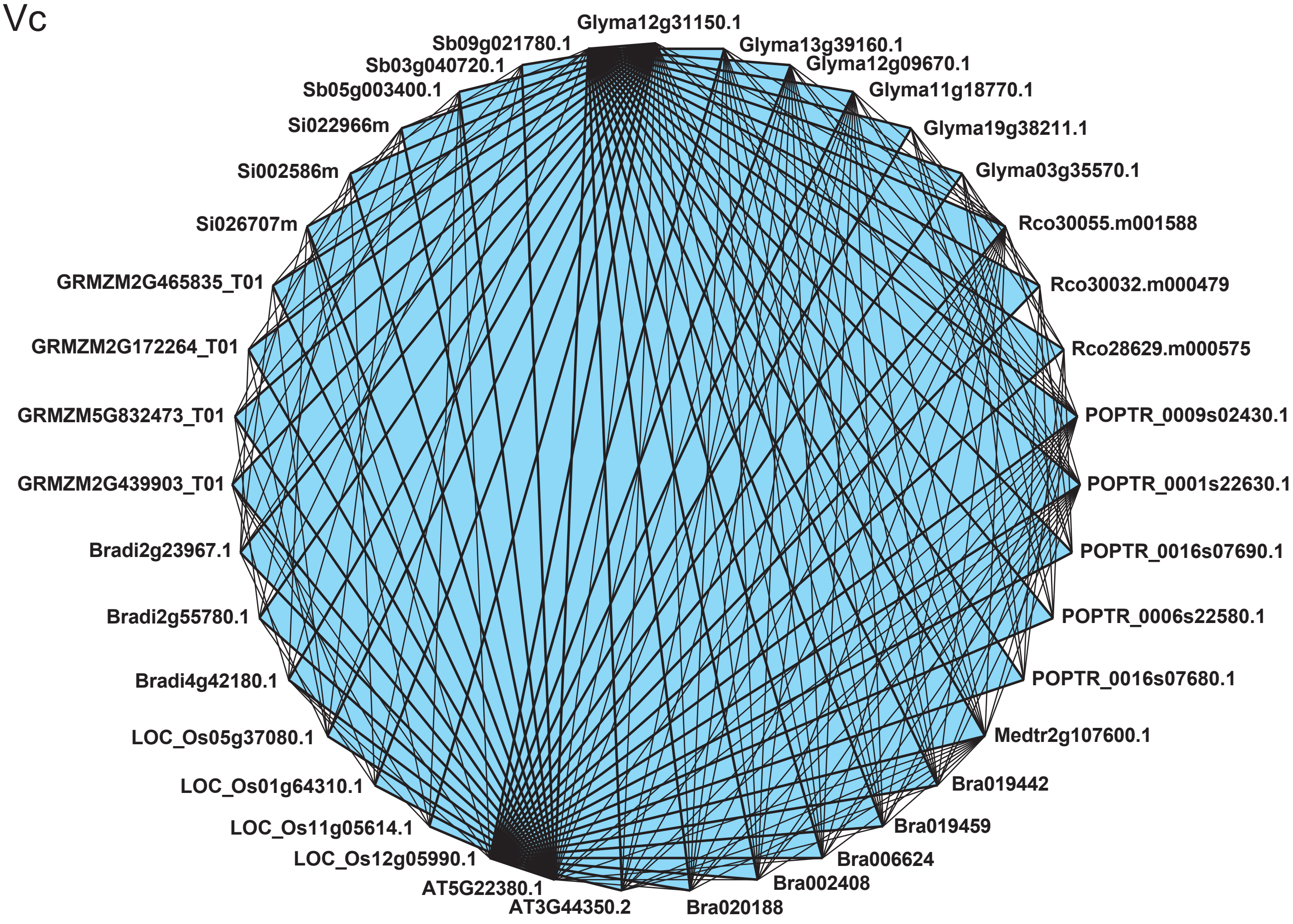
Glyma12g31150.1
Glyma13g39160.1
Glyma12g09670.1
Glyma11g18770.1
Glyma19g38211.1
Glyma03g35570.1
Rco30055.m001588
Rco30032.m000479
Rco28629.m000575
POPTR_0009s02430.1
POPTR_0001s22630.1
POPTR_0016s07690.1
POPTR_0006s22580.1
POPTR_0016s07680.1
Medtr2g107600.1
Bra019442
Bra019459
Bra006624
Bra002408
Bra020188
AT3G44350.2
AT5G22380.1
LOC_Os12g05990.1
LOC_Os11g05614.1
LOC_Os01g64310.1
LOC_Os05g37080.1
Bradi4g42180.1
Bradi2g55780.1
Bradi2g23967.1
GRMZM2G439903_T01
GRMZM5G832473_T01
GRMZM2G172264_T01
GRMZM2G465835_T01
Si026707m
Si002586m
Si022966m
Sb05g003400.1
Sb03g040720.1
Sb09g021780.1

IVd_1

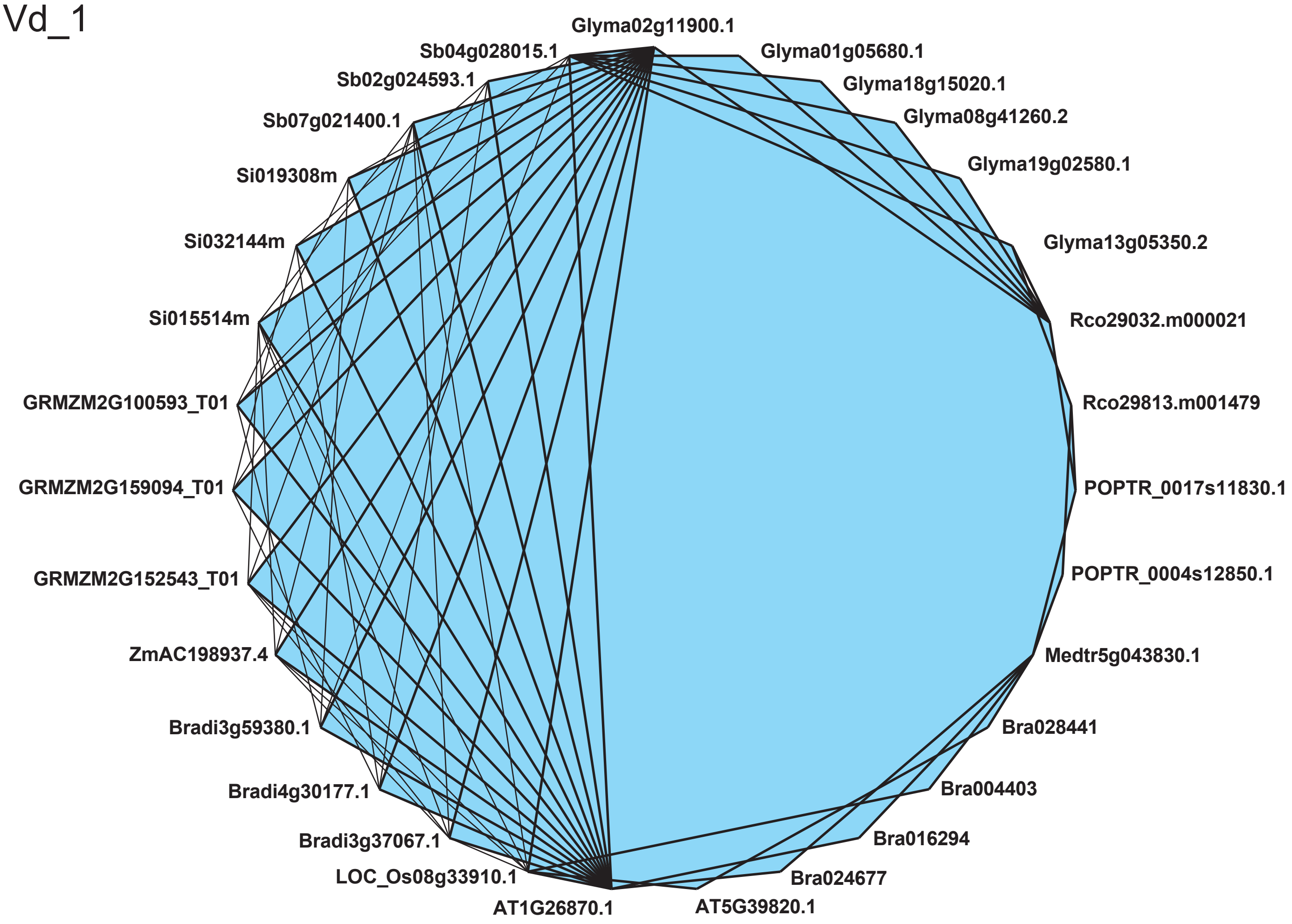
Glyma02g11900.1
Glyma01g05680.1
Glyma18g15020.1
Glyma08g41260.2
Glyma19g02580.1
Glyma13g05350.2
Rco29032.m000021
Rco29813.m001479
POPTR_0017s11830.1
POPTR_0004s12850.1
Medtr5g043830.1
Bra028441
Bra004403
Bra016294
Bra024677
AT5G39820.1
AT1G26870.1
LOC_Os08g33910.1
Bradi3g37067.1
Bradi4g30177.1
Bradi3g59380.1
ZmAC198937.4
GRMZM2G152543_T01
GRMZM2G159094_T01
GRMZM2G100593_T01
Si015514m
Si032144m
Si019308m
Sb07g021400.1
Sb02g024593.1
Sb04g028015.1

IVd_2

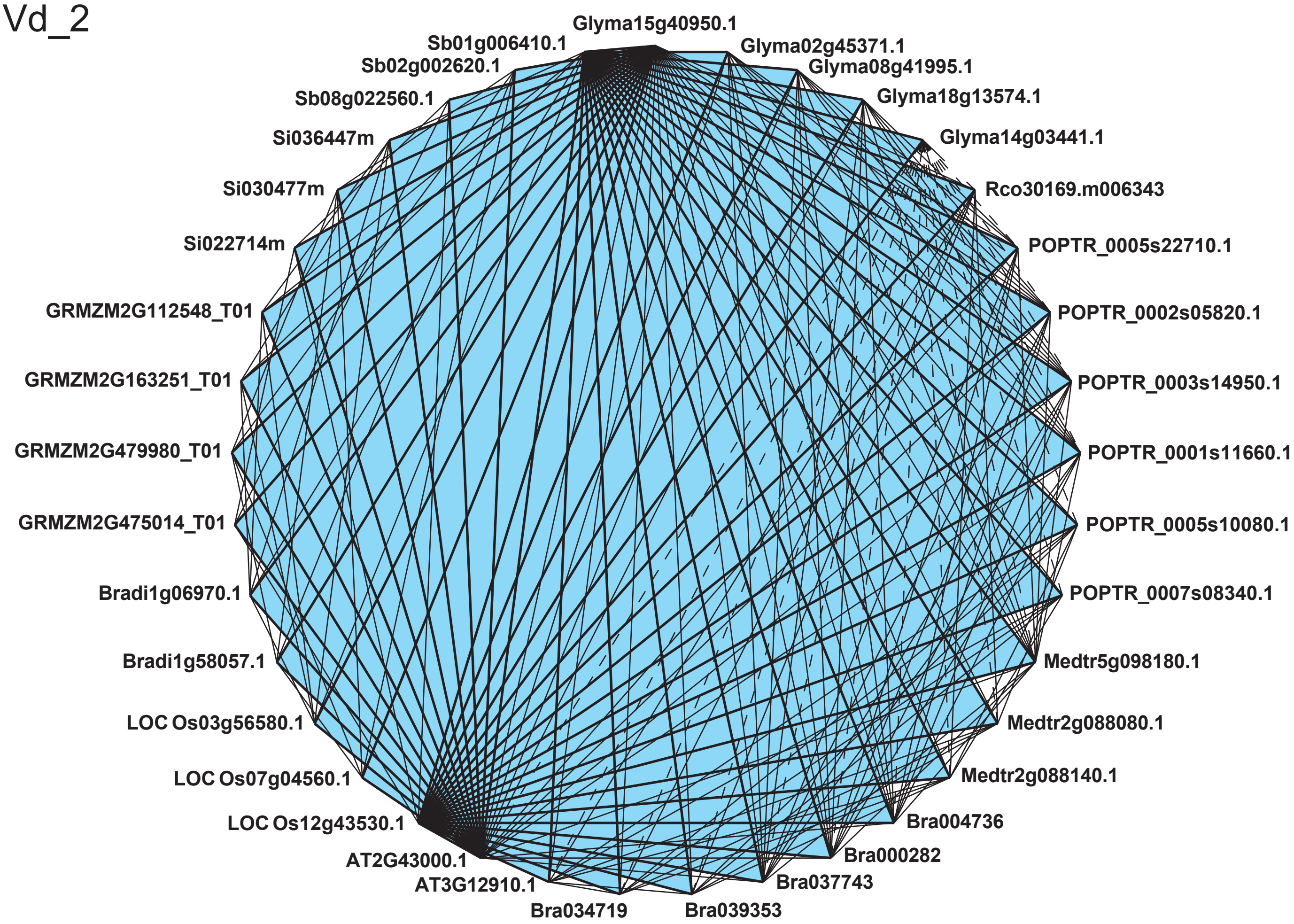
Glyma15g40950.1
Glyma02g45371.1
Glyma08g41995.1
Glyma18g13574.1
Glyma14g03441.1
Rco30169.m006343
POPTR_0005s22710.1
POPTR_0002s05820.1
POPTR_0003s14950.1
POPTR_0001s11660.1
POPTR_0005s10080.1
POPTR_0007s08340.1
Medtr5g098180.1
Medtr2g088080.1
Medtr2g088140.1
Bra004736
Bra000282
Bra037743
Bra039353
Bra034719
AT3G12910.1
AT2G43000.1
LOC Os12g43530.1
LOC Os07g04560.1
LOC Os03g56580.1
Bradi1g58057.1
Bradi1g06970.1
GRMZM2G475014_T01
GRMZM2G479980_T01
GRMZM2G163251_T01
GRMZM2G112548_T01
Si022714m
Si030477m
Si036447m
Sb08g022560.1
Sb02g002620.1
Sb01g006410.1

Va_1

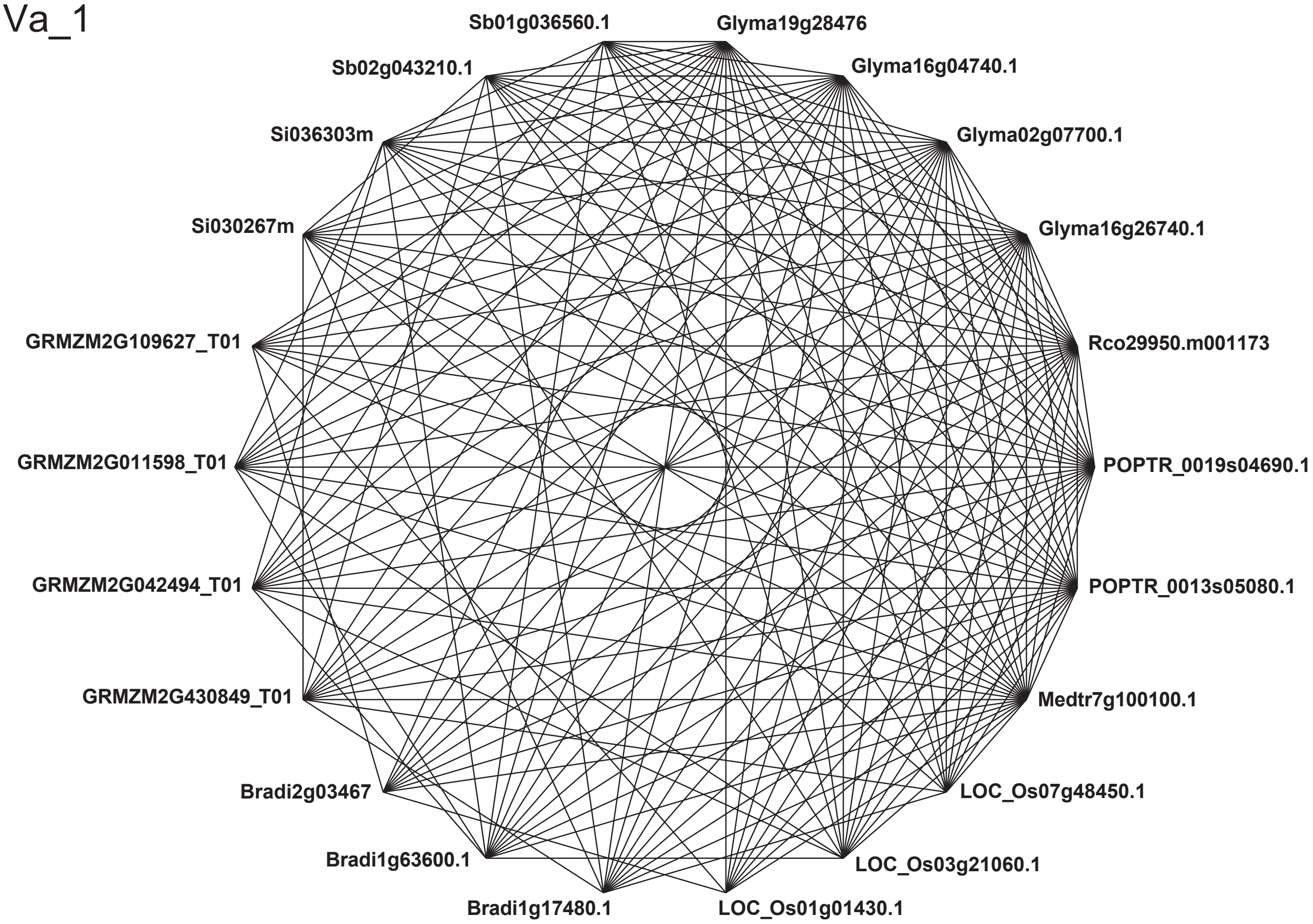
Sb01g036560.1
Glyma19g28476
Glyma16g04740.1
Glyma02g07700.1
Glyma16g26740.1
Rco29950.m001173
POPTR_0019s04690.1
POPTR_0013s05080.1
Medtr7g100100.1
LOC_Os07g48450.1
LOC_Os03g21060.1
LOC_Os01g01430.1
Bradi1g17480.1
Bradi1g63600.1
Bradi2g03467
GRMZM2G430849_T01
GRMZM2G042494_T01
GRMZM2G011598_T01
GRMZM2G109627_T01
Si030267m
Si036303m
Sb02g043210.1

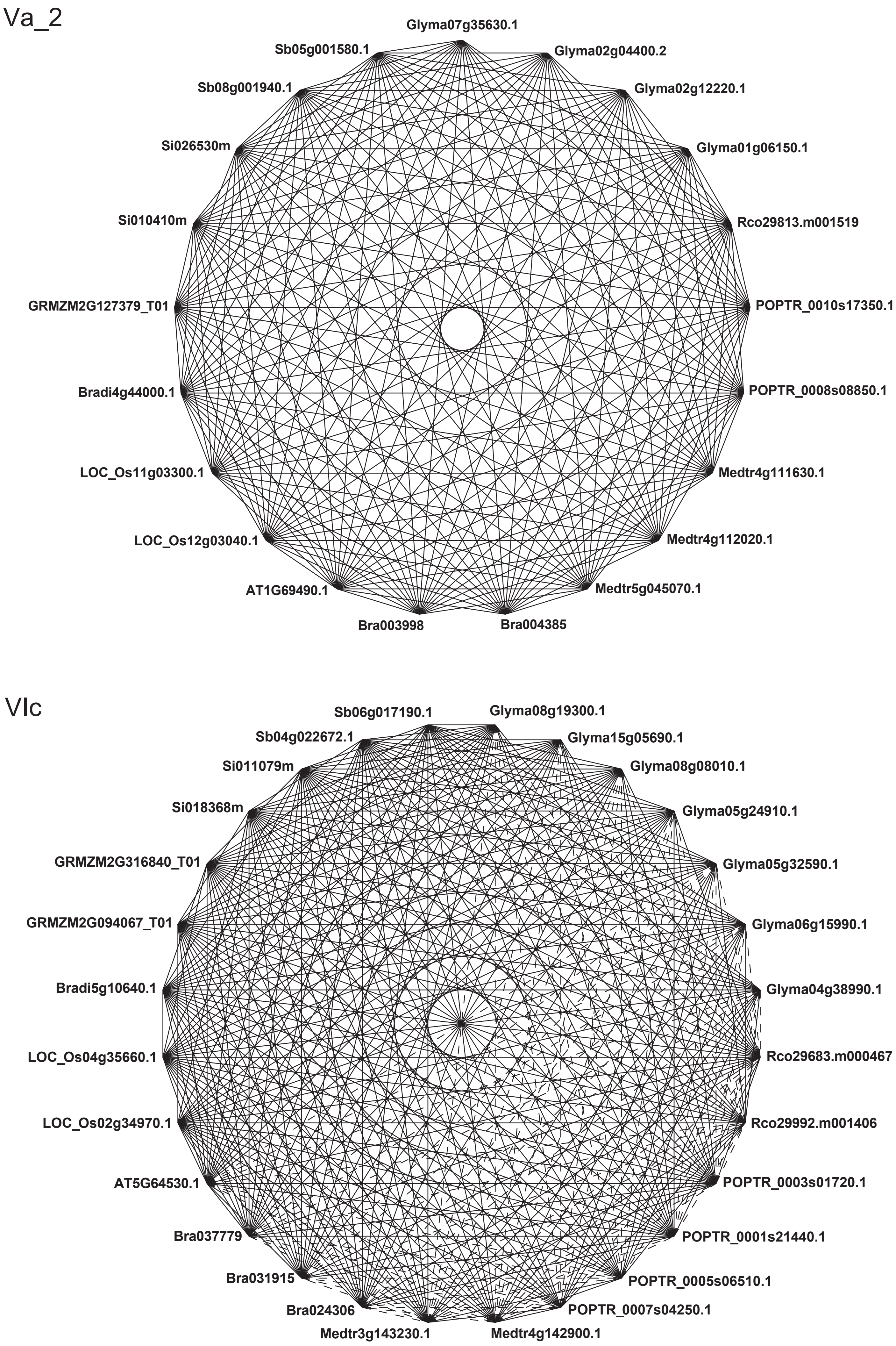

Figure S1. COGs of the subfamilies considered in this study. Solid lines show reciprocal BeTs (Best Hits) and broken lines show asymmetrical BeTs. Genes from the same species are adjacent. To avoid too many lines, in some COGs the reciprocal BeTs are simply represented with blue shades and thicken lines as the bondaries (e.g., in the COG of Ia_2, the reciprocal BeTs of LOC_Os11g03370.1 are GRMZM2G018436_T01, Si010417m, Si026649m, Sb05g001590.1, and the genes listed between AT5G18270.1 and Glyma16g04720.1, that is, all the dicot genes in this COG; while the reciprocal BeTs of Rco29950.m001171 are all the other genes in the COG). Each gene ID is indicated, and the prefix "Rco" denotes genes from *Ricinus communis*.
