# Supplementary material for: Divergent Evolutionary Patterns of NAC Transcription Factors Are Associated with Diversification and Gene Duplications in Angiosperm
Source: Front Plant Sci. 2017 Jun 30;8:1156. doi: 10.3389/fpls.2017.01156 (PMC5492850; doi:10.3389/fpls.2017.01156)
Supplement: Supplementary file 3 [file Presentation2.PDF]

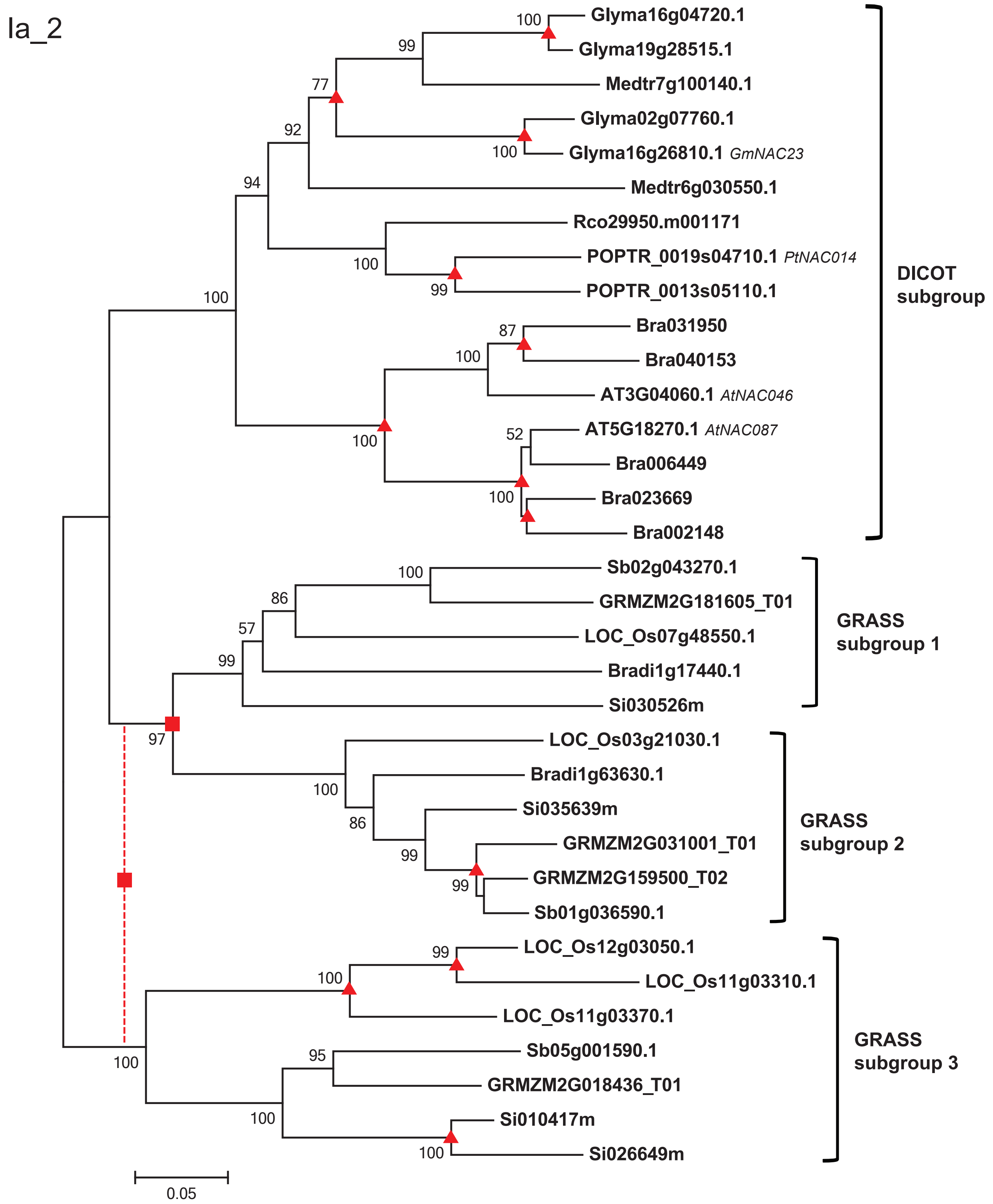
Ia_2
Glyma16g04720.1
Glyma19g28515.1
Medtr7g100140.1
Glyma02g07760.1
Glyma16g26810.1 GmNAC23
Medtr6g030550.1
Rco29950.m001171
POPTR_0019s04710.1 PtNAC014
POPTR_0013s05110.1
Bra031950
Bra040153
AT3G04060.1 AtNAC046
AT5G18270.1 AtNAC087
Bra006449
Bra023669
Bra002148
DICOT subgroup
Sb02g043270.1
GRMZM2G181605_T01
LOC_Os07g48550.1
Bradi1g17440.1
Si030526m
GRASS subgroup 1
LOC_Os03g21030.1
Bradi1g63630.1
Si035639m
GRMZM2G031001_T01
GRMZM2G159500_T02
Sb01g036590.1
GRASS subgroup 2
LOC_Os12g03050.1
LOC_Os11g03310.1
LOC_Os11g03370.1
Sb05g001590.1
GRMZM2G018436_T01
Si010417m
Si026649m
GRASS subgroup 3
0.05

Ia_3

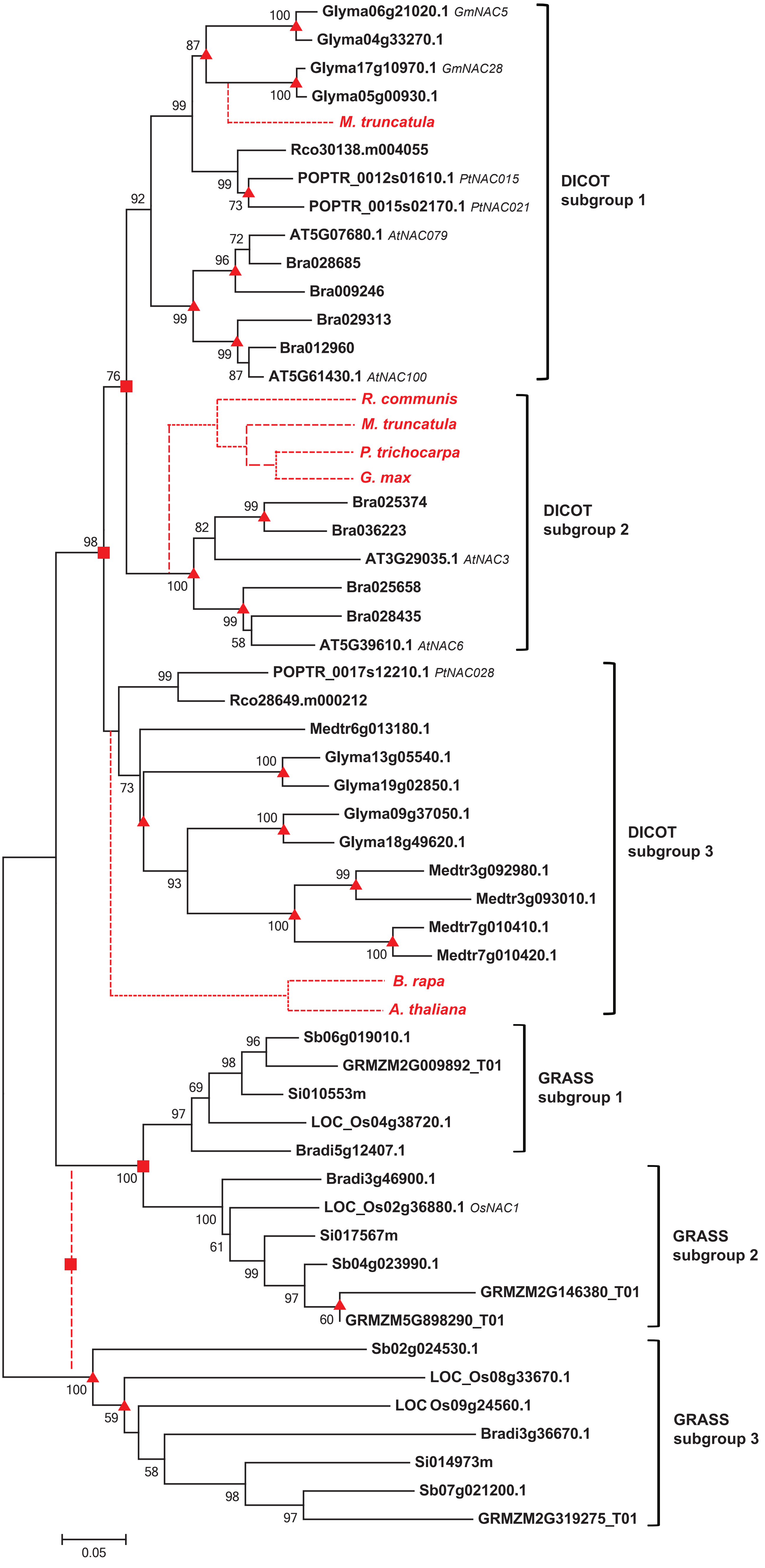
Glyma06g21020.1 GmNAC5
Glyma04g33270.1
Glyma17g10970.1 GmNAC28
Glyma05g00930.1
M. truncatula
Rco30138.m004055
POPTR_0012s01610.1 PtNAC015
POPTR_0015s02170.1 PtNAC021
AT5G07680.1 AtNAC079
Bra028685
Bra009246
Bra029313
Bra012960
AT5G61430.1 AtNAC100
DICOT subgroup 1
R. communis
M. truncatula
P. trichocarpa
G. max
Bra025374
Bra036223
AT3G29035.1 AtNAC3
Bra025658
Bra028435
AT5G39610.1 AtNAC6
DICOT subgroup 2
POPTR_0017s12210.1 PtNAC028
Rco28649.m000212
Medtr6g013180.1
Glyma13g05540.1
Glyma19g02850.1
Glyma09g37050.1
Glyma18g49620.1
Medtr3g092980.1
Medtr3g093010.1
Medtr7g010410.1
Medtr7g010420.1
B. rapa
A. thaliana
DICOT subgroup 3
Sb06g019010.1
GRMZM2G009892_T01
Si010553m
LOC_Os04g38720.1
Bradi5g12407.1
GRASS subgroup 1
Bradi3g46900.1
LOC_Os02g36880.1 OsNAC1
Si017567m
Sb04g023990.1
GRMZM2G146380_T01
GRMZM5G898290_T01
GRASS subgroup 2
Sb02g024530.1
LOC_Os08g33670.1
LOC Os09g24560.1
Bradi3g36670.1
Si014973m
Sb07g021200.1
GRMZM2G319275_T01
GRASS subgroup 3
0.05

Ia_4

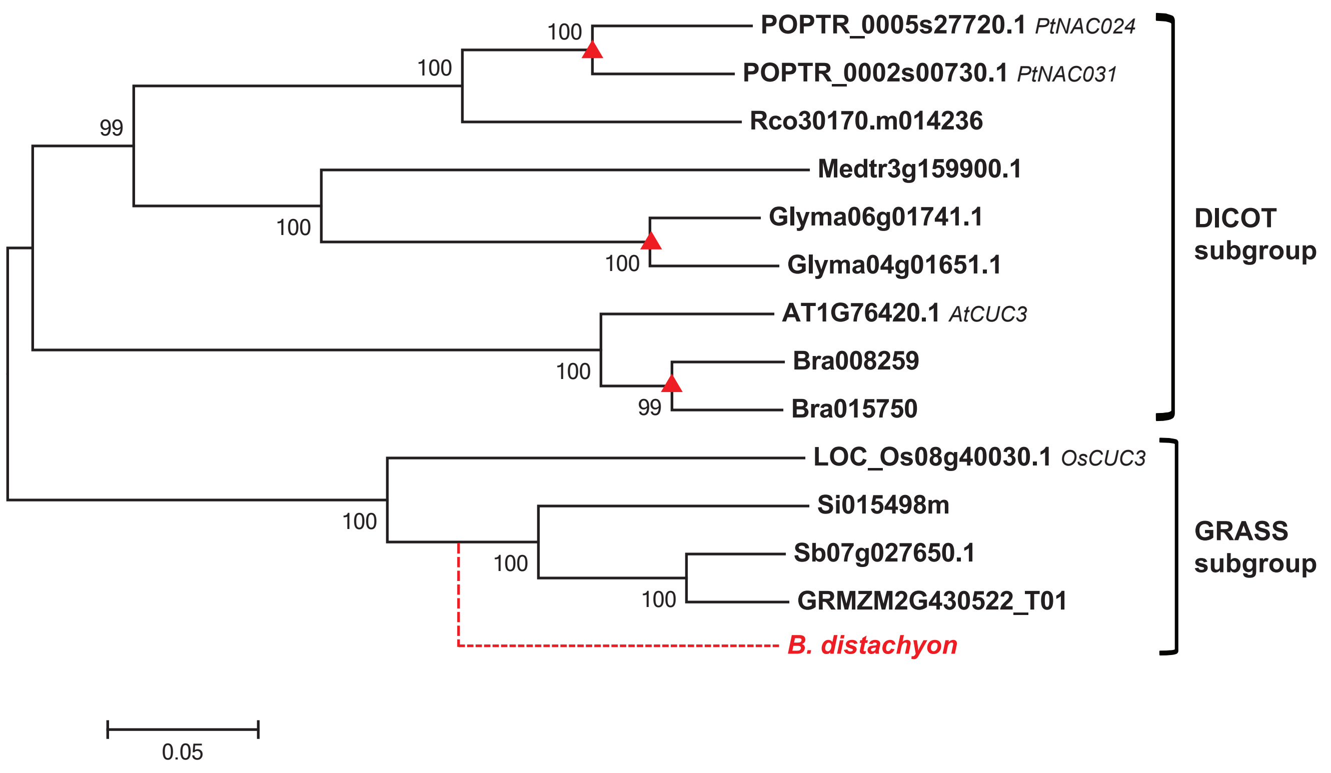
POPTR_0005s27720.1 PtNAC024
POPTR_0002s00730.1 PtNAC031
Rco30170.m014236
Medtr3g159900.1
Glyma06g01741.1
Glyma04g01651.1
AT1G76420.1 AtCUC3
Bra008259
Bra015750
LOC_Os08g40030.1 OsCUC3
Si015498m
Sb07g027650.1
GRMZM2G430522_T01
B. distachyon
DICOT subgroup
GRASS subgroup
100
100
99
100
100
100
99
100
100
100
0.05

Ia_5

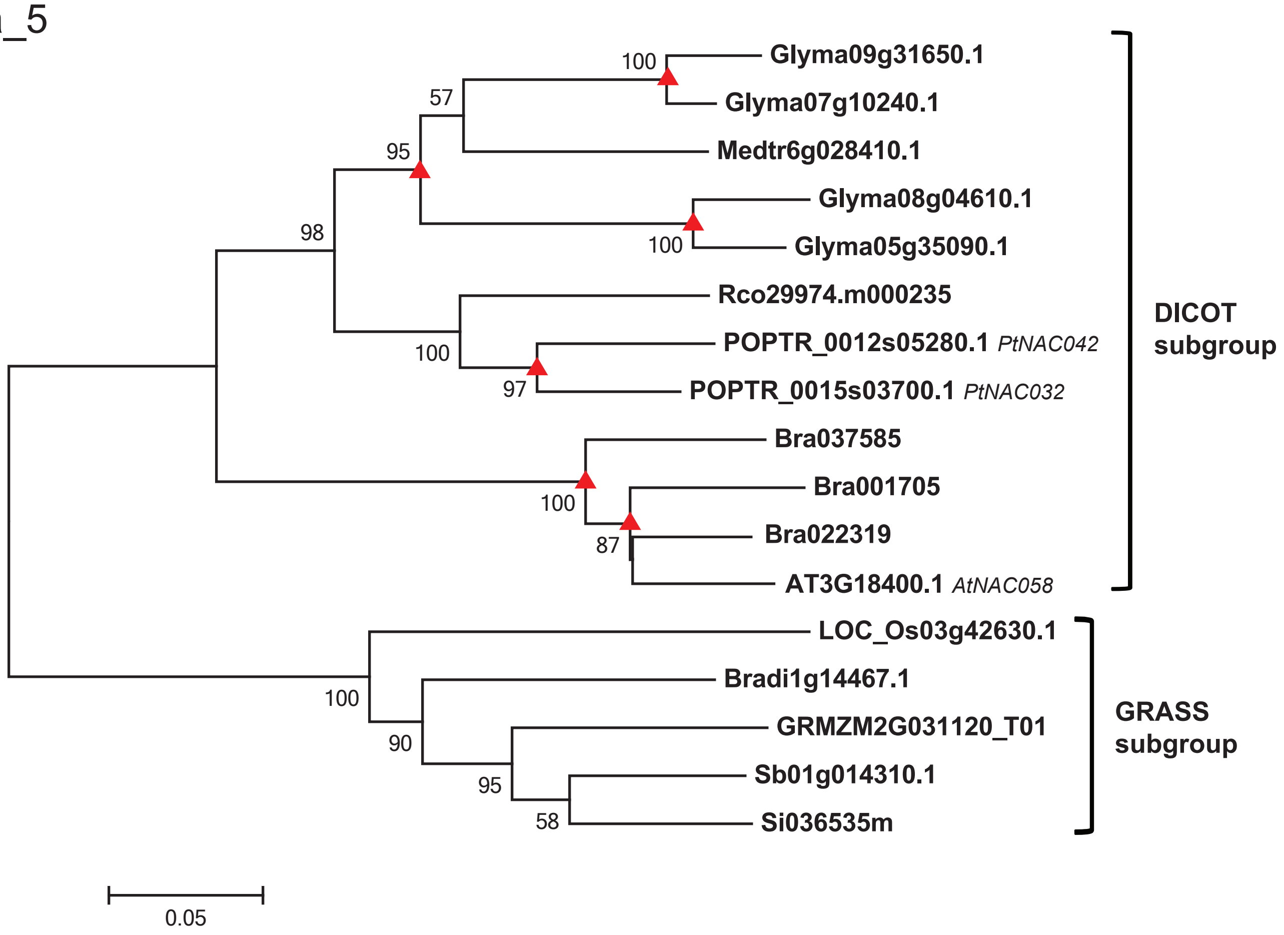
Glyma09g31650.1
Glyma07g10240.1
Medtr6g028410.1
Glyma08g04610.1
Glyma05g35090.1
Rco29974.m000235
POPTR_0012s05280.1 PtNAC042
POPTR_0015s03700.1 PtNAC032
Bra037585
Bra001705
Bra022319
AT3G18400.1 AtNAC058
LOC_Os03g42630.1
Bradi1g14467.1
GRMZM2G031120_T01
Sb01g014310.1
Si036535m
DICOT subgroup
GRASS subgroup
100
57
95
98
100
100
97
100
87
100
90
95
58
0.05

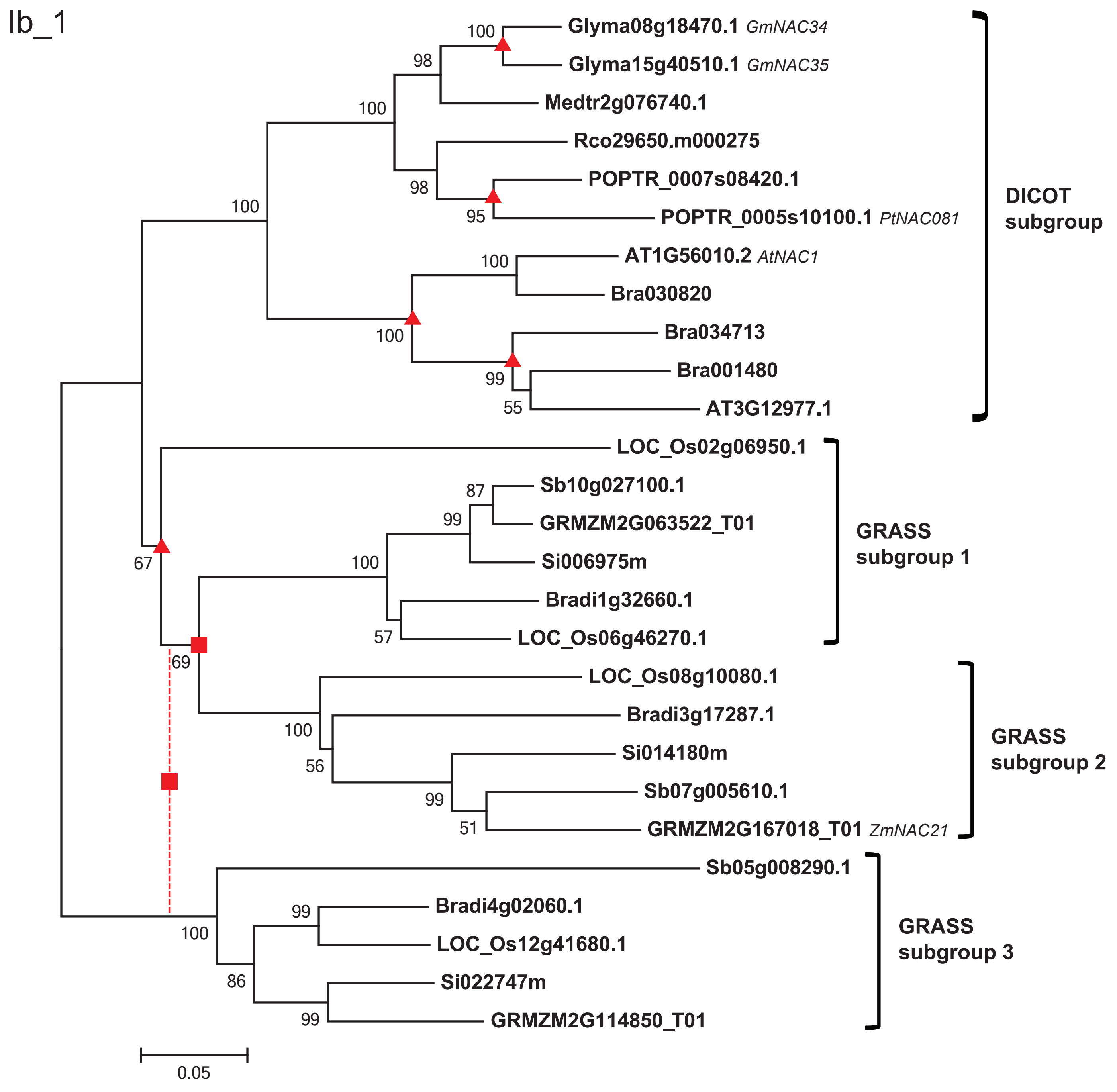
Ib_1
Glyma08g18470.1 GmNAC34
Glyma15g40510.1 GmNAC35
Medtr2g076740.1
Rco29650.m000275
POPTR_0007s08420.1
POPTR_0005s10100.1 PtNAC081
AT1G56010.2 AtNAC1
Bra030820
Bra034713
Bra001480
AT3G12977.1
DICOT subgroup
LOC_Os02g06950.1
Sb10g027100.1
GRMZM2G063522_T01
Si006975m
Bradi1g32660.1
LOC_Os06g46270.1
GRASS subgroup 1
LOC_Os08g10080.1
Bradi3g17287.1
Si014180m
Sb07g005610.1
GRMZM2G167018_T01 ZmNAC21
GRASS subgroup 2
Sb05g008290.1
Bradi4g02060.1
LOC_Os12g41680.1
Si022747m
GRMZM2G114850_T01
GRASS subgroup 3
100
98
100
98
95
100
100
100
100
99
55
67
87
99
100
57
69
100
56
99
51
100
99
86
99
0.05

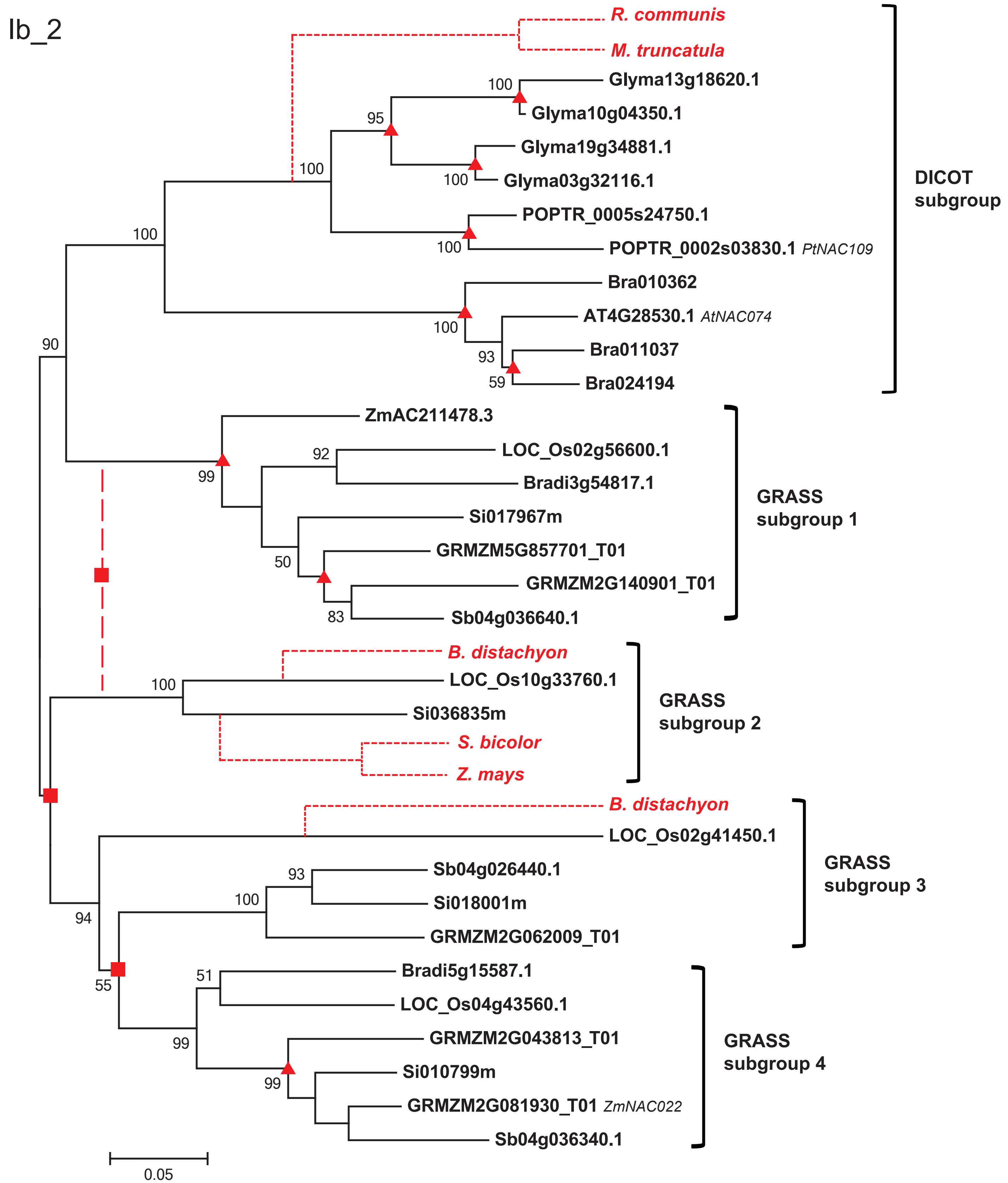
Ib_2
R. communis
M. truncatula
Glyma13g18620.1
Glyma10g04350.1
Glyma19g34881.1
Glyma03g32116.1
POPTR_0005s24750.1
POPTR_0002s03830.1 PtNAC109
Bra010362
AT4G28530.1 AtNAC074
Bra011037
Bra024194
DICOT subgroup
ZmAC211478.3
LOC_Os02g56600.1
Bradi3g54817.1
Si017967m
GRMZM5G857701_T01
GRMZM2G140901_T01
Sb04g036640.1
GRASS subgroup 1
B. distachyon
LOC_Os10g33760.1
Si036835m
S. bicolor
Z. mays
GRASS subgroup 2
B. distachyon
LOC_Os02g41450.1
Sb04g026440.1
Si018001m
GRMZM2G062009_T01
GRASS subgroup 3
Bradi5g15587.1
LOC_Os04g43560.1
GRMZM2G043813_T01
Si010799m
GRMZM2G081930_T01 ZmNAC022
Sb04g036340.1
GRASS subgroup 4
0.05

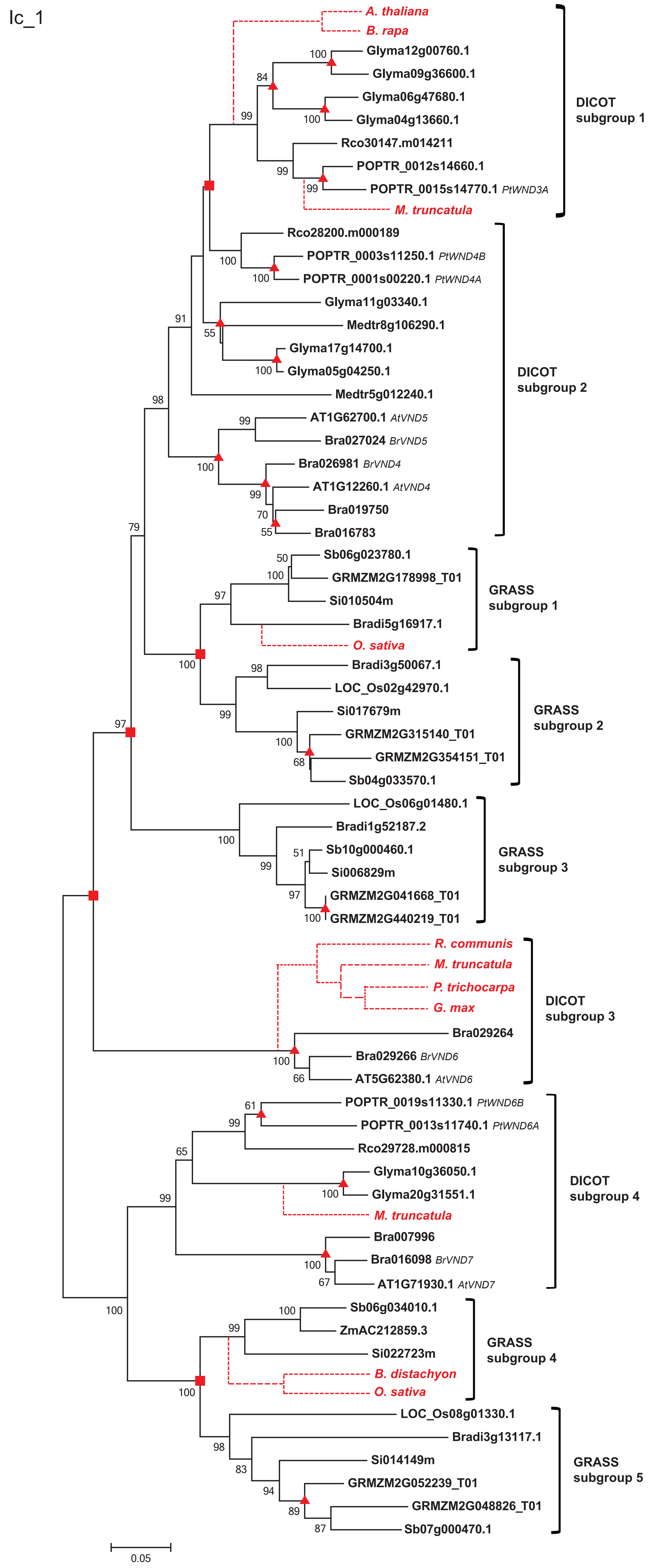
Ic_1
A. thaliana
B. rapa
Glyma12g00760.1
Glyma09g36600.1
Glyma06g47680.1
Glyma04g13660.1
Rco30147.m014211
POPTR_0012s14660.1
POPTR_0015s14770.1 PtWND3A
M. truncatula
DICOT subgroup 1
Rco28200.m000189
POPTR_0003s11250.1 PtWND4B
POPTR_0001s00220.1 PtWND4A
Glyma11g03340.1
Medtr8g106290.1
Glyma17g14700.1
Glyma05g04250.1
Medtr5g012240.1
AT1G62700.1 AtVND5
Bra027024 BrVND5
Bra026981 BrVND4
AT1G12260.1 AtVND4
Bra019750
Bra016783
DICOT subgroup 2
Sb06g023780.1
GRMZM2G178998_T01
Si010504m
Bradi5g16917.1
O. sativa
GRASS subgroup 1
Bradi3g50067.1
LOC_Os02g42970.1
Si017679m
GRMZM2G315140_T01
GRMZM2G354151_T01
Sb04g033570.1
GRASS subgroup 2
LOC_Os06g01480.1
Bradi1g52187.2
Sb10g000460.1
Si006829m
GRMZM2G041668_T01
GRMZM2G440219_T01
GRASS subgroup 3
R. communis
M. truncatula
P. trichocarpa
G. max
Bra029264
Bra029266 BrVND6
AT5G62380.1 AtVND6
DICOT subgroup 3
POPTR_0019s11330.1 PtWND6B
POPTR_0013s11740.1 PtWND6A
Rco29728.m000815
Glyma10g36050.1
Glyma20g31551.1
M. truncatula
Bra007996
Bra016098 BrVND7
AT1G71930.1 AtVND7
DICOT subgroup 4
Sb06g034010.1
ZmAC212859.3
Si022723m
B. distachyon
O. sativa
GRASS subgroup 4
LOC_Os08g01330.1
Bradi3g13117.1
Si014149m
GRMZM2G052239_T01
GRMZM2G048826_T01
Sb07g000470.1
GRASS subgroup 5
0.05

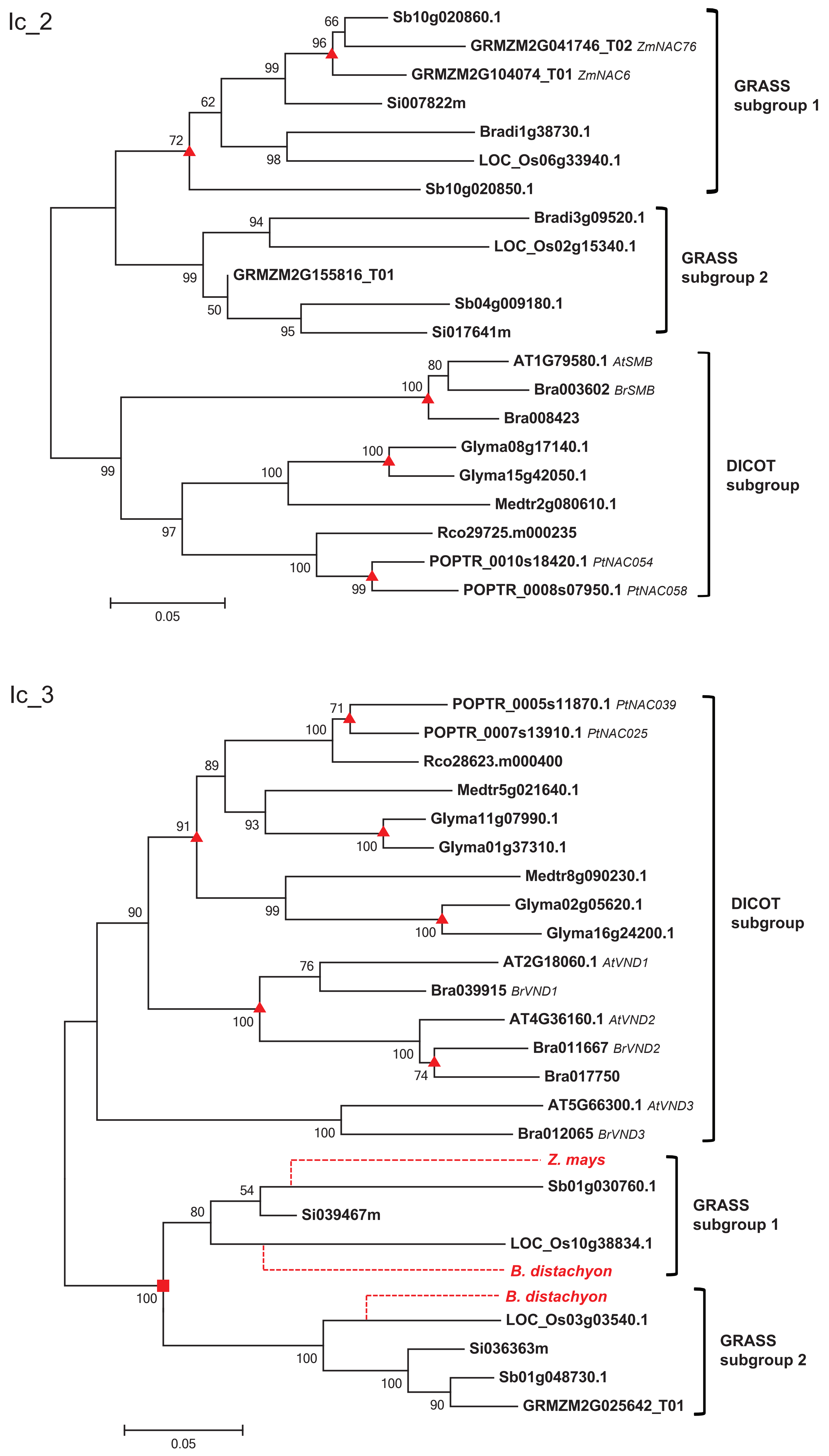

Ic_2
Sb10g020860.1
GRMZM2G041746_T02 ZmNAC76
GRMZM2G104074_T01 ZmNAC6
Si007822m
Bradi1g38730.1
LOC_Os06g33940.1
Sb10g020850.1
GRASS subgroup 1
Bradi3g09520.1
LOC_Os02g15340.1
GRMZM2G155816_T01
Sb04g009180.1
Si017641m
GRASS subgroup 2
AT1G79580.1 AtSMB
Bra003602 BrSMB
Bra008423
Glyma08g17140.1
Glyma15g42050.1
Medtr2g080610.1
Rco29725.m000235
POPTR_0010s18420.1 PtNAC054
POPTR_0008s07950.1 PtNAC058
DICOT subgroup
0.05
Ic_3
POPTR_0005s11870.1 PtNAC039
POPTR_0007s13910.1 PtNAC025
Rco28623.m000400
Medtr5g021640.1
Glyma11g07990.1
Glyma01g37310.1
Medtr8g090230.1
Glyma02g05620.1
Glyma16g24200.1
AT2G18060.1 AtVND1
Bra039915 BrVND1
AT4G36160.1 AtVND2
Bra011667 BrVND2
Bra017750
AT5G66300.1 AtVND3
Bra012065 BrVND3
DICOT subgroup
Z. mays
Sb01g030760.1
Si039467m
LOC_Os10g38834.1
B. distachyon
GRASS subgroup 1
B. distachyon
LOC_Os03g03540.1
Si036363m
Sb01g048730.1
GRMZM2G025642_T01
GRASS subgroup 2
0.05

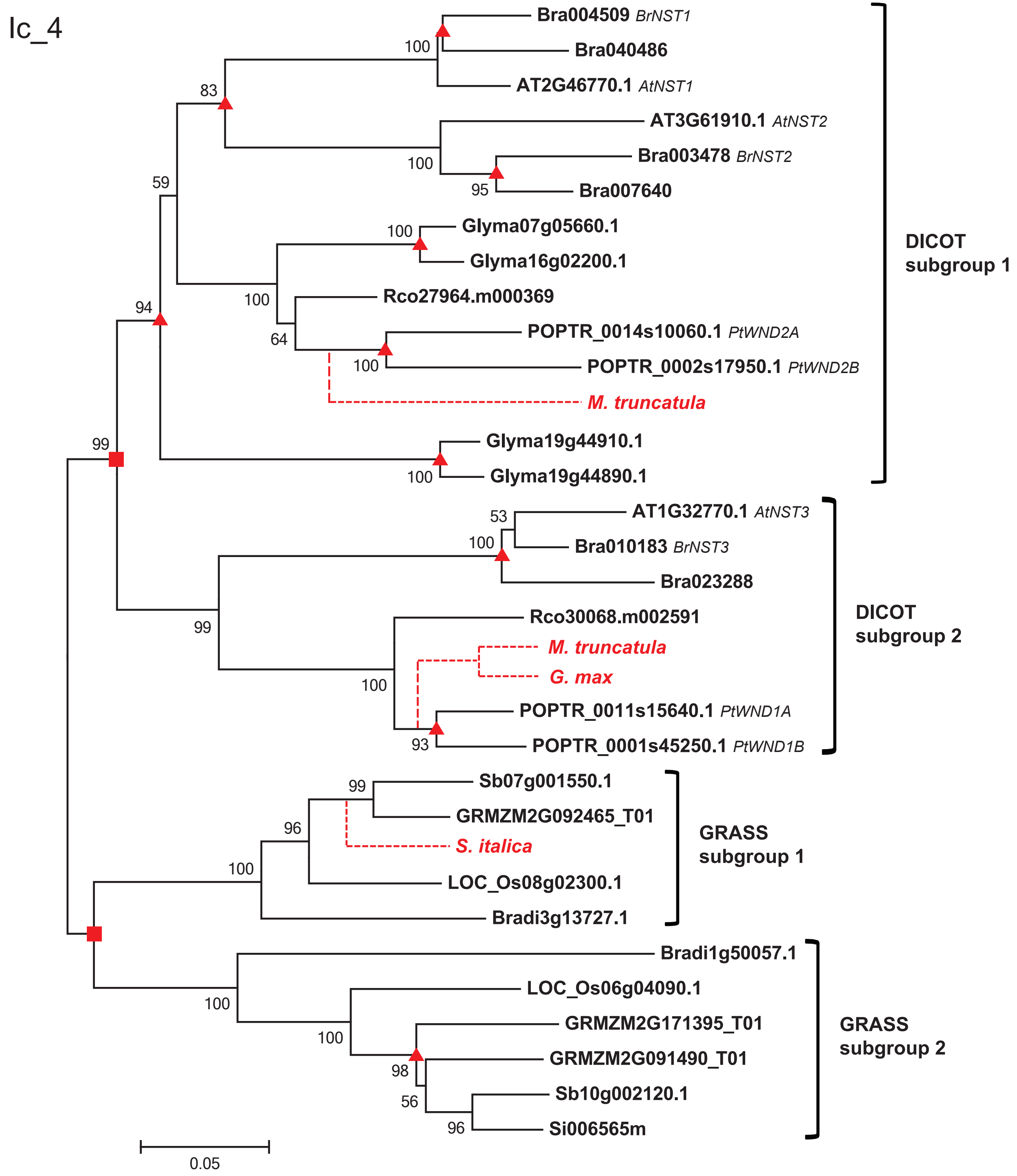

Ic_4
Bra004509 BrNST1
Bra040486
AT2G46770.1 AtNST1
AT3G61910.1 AtNST2
Bra003478 BrNST2
Bra007640
Glyma07g05660.1
Glyma16g02200.1
Rco27964.m000369
POPTR_0014s10060.1 PtWND2A
POPTR_0002s17950.1 PtWND2B
M. truncatula
Glyma19g44910.1
Glyma19g44890.1
DICOT subgroup 1
AT1G32770.1 AtNST3
Bra010183 BrNST3
Bra023288
Rco30068.m002591
M. truncatula
G. max
POPTR_0011s15640.1 PtWND1A
POPTR_0001s45250.1 PtWND1B
DICOT subgroup 2
Sb07g001550.1
GRMZM2G092465_T01
S. italica
LOC_Os08g02300.1
Bradi3g13727.1
GRASS subgroup 1
Bradi1g50057.1
LOC_Os06g04090.1
GRMZM2G171395_T01
GRMZM2G091490_T01
Sb10g002120.1
Si006565m
GRASS subgroup 2
100
83
100
95
59
100
100
64
100
94
99
100
53
100
99
100
93
99
96
100
100
100
98
56
96
0.05

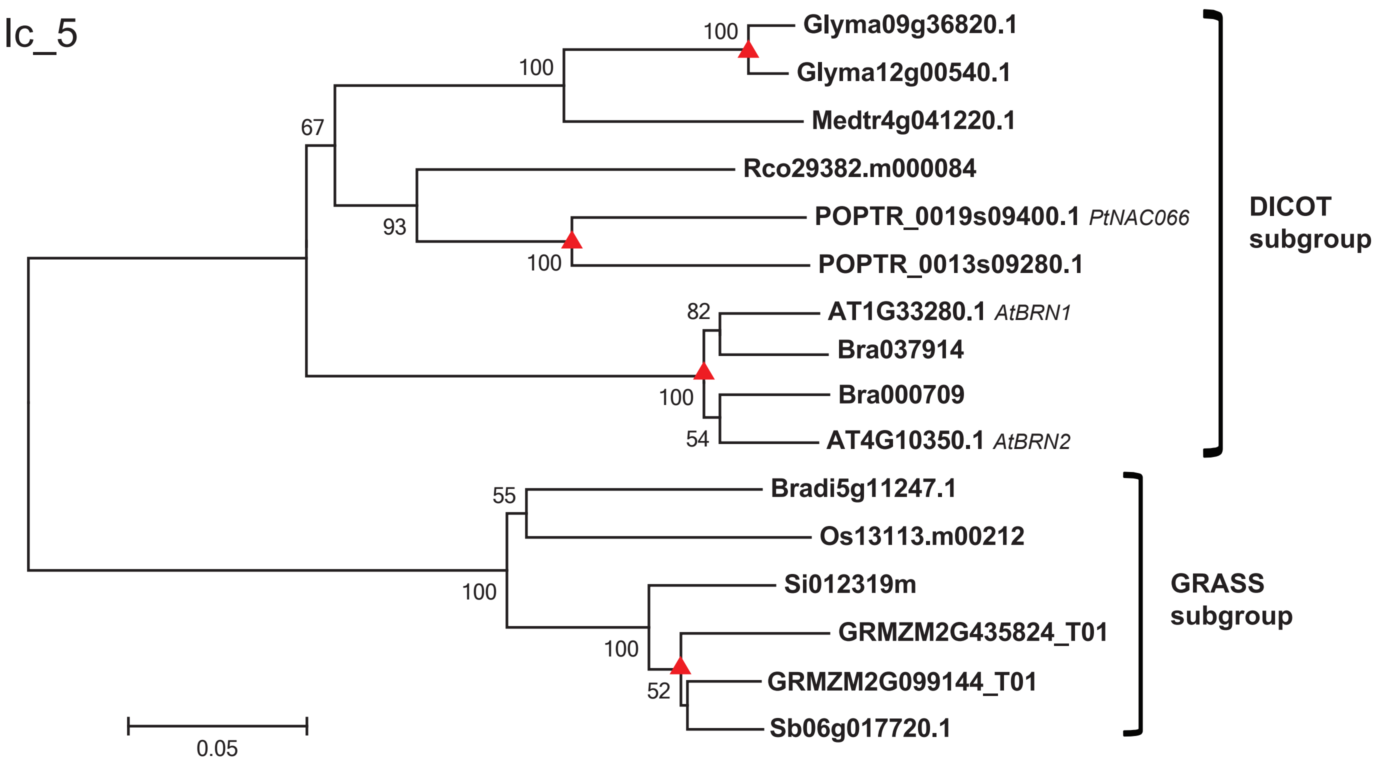
Ic_5
Glyma09g36820.1
Glyma12g00540.1
Medtr4g041220.1
Rco29382.m000084
POPTR_0019s09400.1 PtNAC066
POPTR_0013s09280.1
AT1G33280.1 AtBRN1
Bra037914
Bra000709
AT4G10350.1 AtBRN2
Bradi5g11247.1
Os13113.m00212
Si012319m
GRMZM2G435824_T01
GRMZM2G099144_T01
Sb06g017720.1
DICOT
subgroup
GRASS
subgroup
0.05

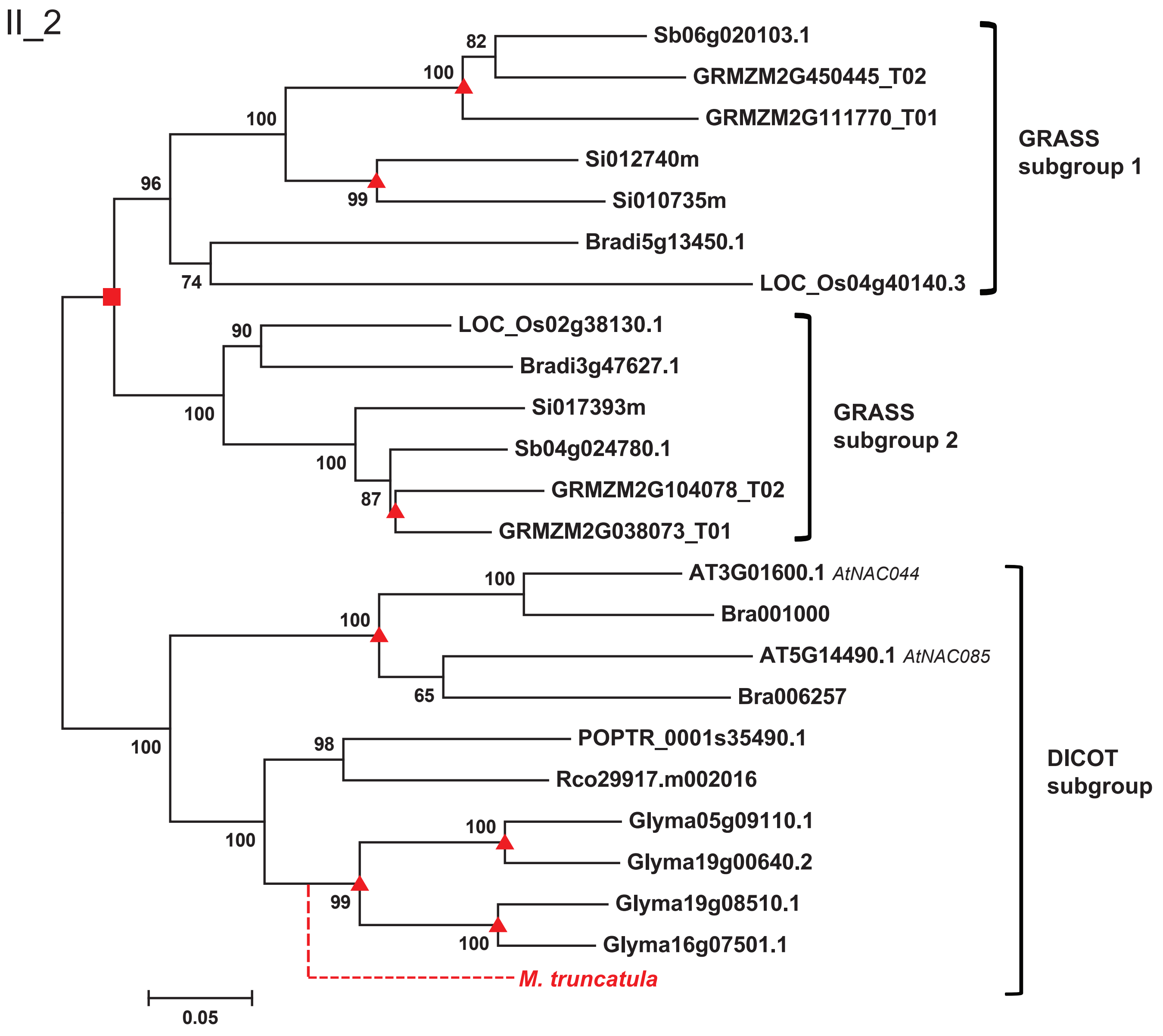
II_2
Sb06g020103.1
GRMZM2G450445_T02
GRMZM2G111770_T01
Si012740m
Si010735m
Bradi5g13450.1
LOC_Os04g40140.3
GRASS
subgroup 1
LOC_Os02g38130.1
Bradi3g47627.1
Si017393m
Sb04g024780.1
GRMZM2G104078_T02
GRMZM2G038073_T01
GRASS
subgroup 2
AT3G01600.1 AtNAC044
Bra001000
AT5G14490.1 AtNAC085
Bra006257
POPTR_0001s35490.1
Rco29917.m002016
Glyma05g09110.1
Glyma19g00640.2
Glyma19g08510.1
Glyma16g07501.1
M. truncatula
DICOT
subgroup
0.05

II_1

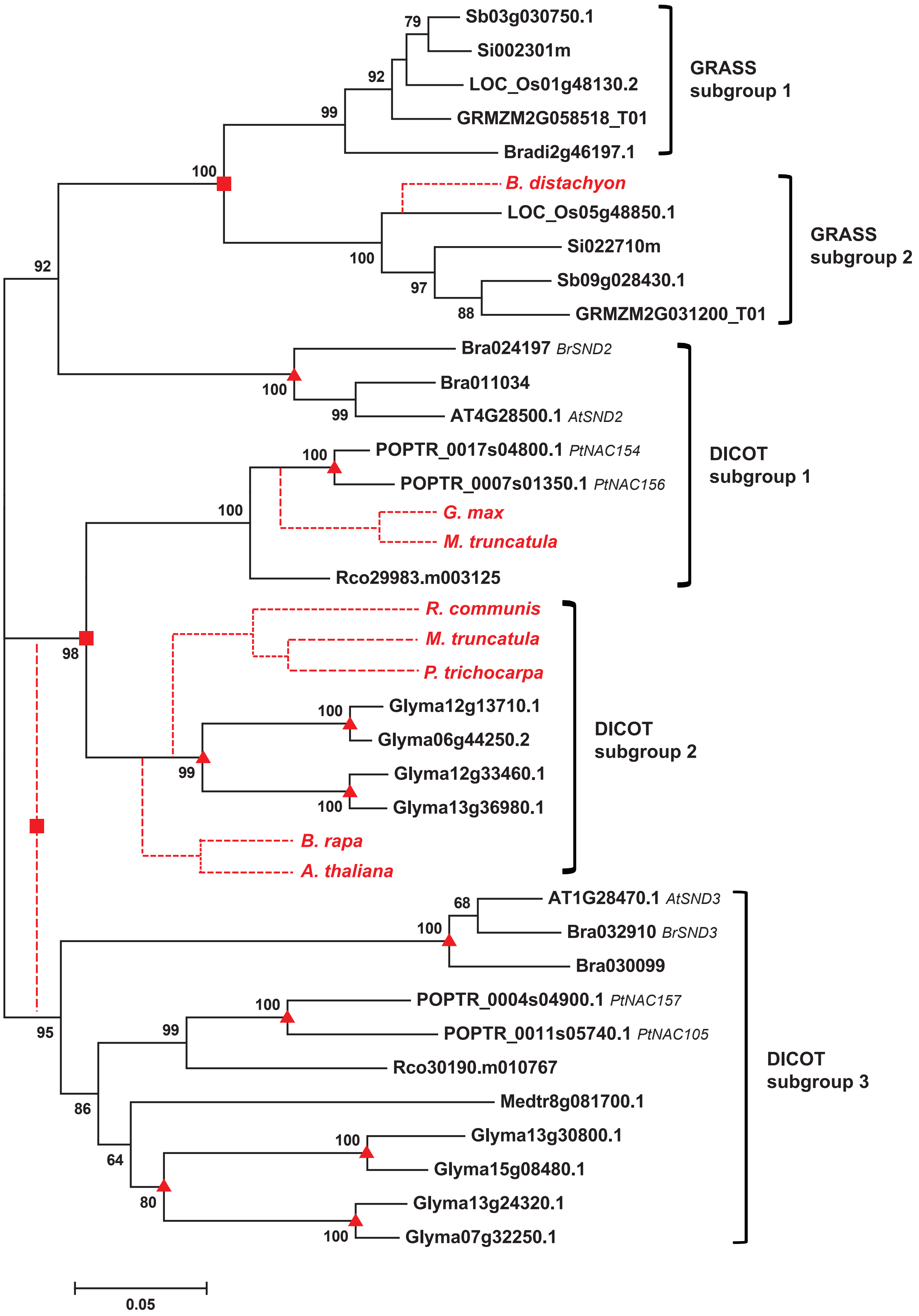

Sb03g030750.1
Si002301m
LOC_Os01g48130.2
GRMZM2G058518_T01
Bradi2g46197.1
GRASS subgroup 1
B. distachyon
LOC_Os05g48850.1
Si022710m
Sb09g028430.1
GRMZM2G031200_T01
GRASS subgroup 2
Bra024197 BrSND2
Bra011034
AT4G28500.1 AtSND2
POPTR_0017s04800.1 PtNAC154
POPTR_0007s01350.1 PtNAC156
G. max
M. truncatula
Rco29983.m003125
DICOT subgroup 1
R. communis
M. truncatula
P. trichocarpa
Glyma12g13710.1
Glyma06g44250.2
Glyma12g33460.1
Glyma13g36980.1
B. rapa
A. thaliana
DICOT subgroup 2
AT1G28470.1 AtSND3
Bra032910 BrSND3
Bra030099
POPTR_0004s04900.1 PtNAC157
POPTR_0011s05740.1 PtNAC105
Rco30190.m010767
Medtr8g081700.1
Glyma13g30800.1
Glyma15g08480.1
Glyma13g24320.1
Glyma07g32250.1
DICOT subgroup 3
79
92
99
100
100
97
88
92
100
99
100
100
98
100
99
100
68
100
100
99
95
86
64
100
80
100
0.05

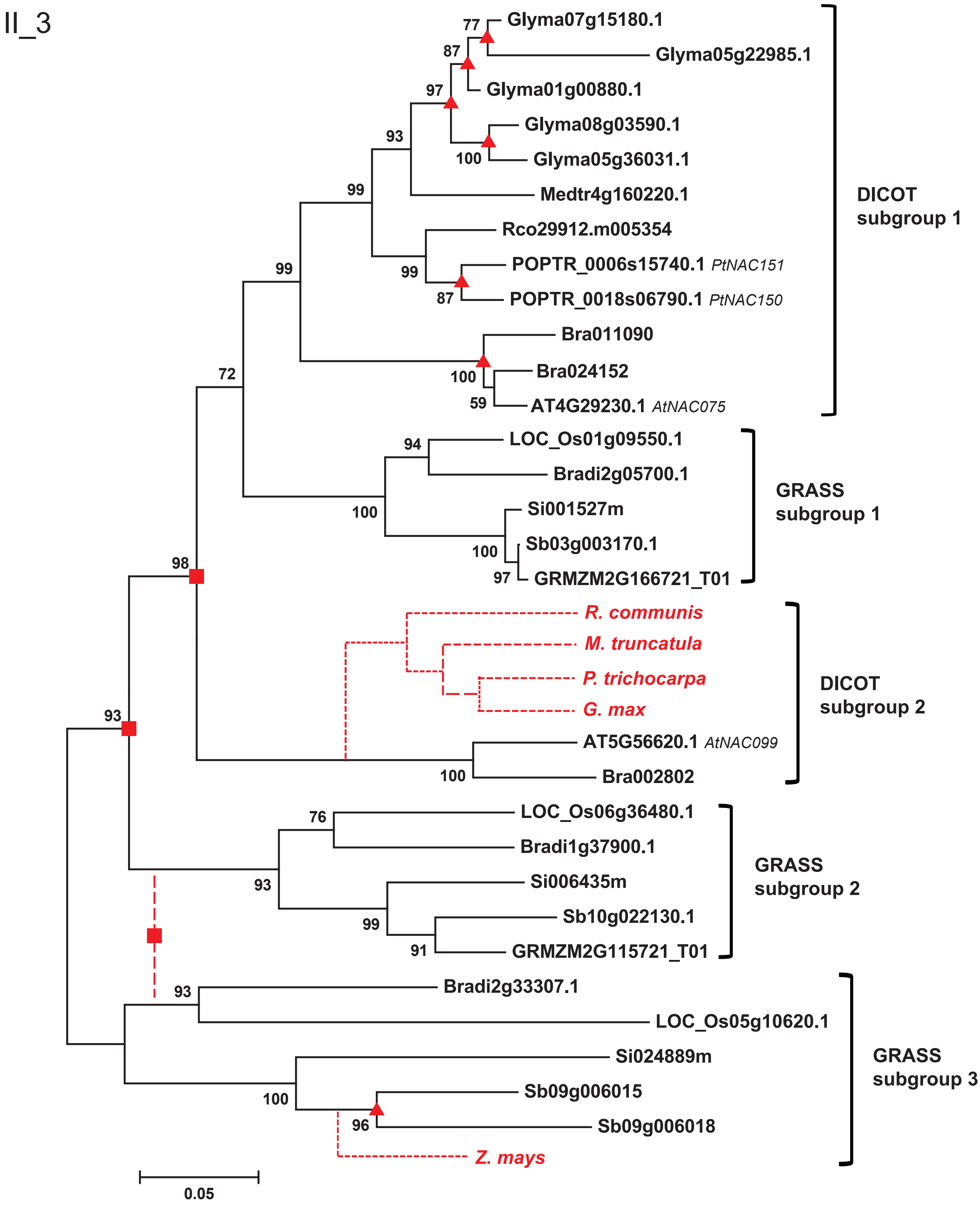
II_3
Glyma07g15180.1
Glyma05g22985.1
Glyma01g00880.1
Glyma08g03590.1
Glyma05g36031.1
Medtr4g160220.1
Rco29912.m005354
POPTR_0006s15740.1 PtNAC151
POPTR_0018s06790.1 PtNAC150
Bra011090
Bra024152
AT4G29230.1 AtNAC075
DICOT subgroup 1
LOC_Os01g09550.1
Bradi2g05700.1
Si001527m
Sb03g003170.1
GRMZM2G166721_T01
GRASS subgroup 1
R. communis
M. truncatula
P. trichocarpa
G. max
AT5G56620.1 AtNAC099
Bra002802
DICOT subgroup 2
LOC_Os06g36480.1
Bradi1g37900.1
Si006435m
Sb10g022130.1
GRMZM2G115721_T01
GRASS subgroup 2
Bradi2g33307.1
LOC_Os05g10620.1
Si024889m
Sb09g006015
Sb09g006018
Z. mays
GRASS subgroup 3
0.05

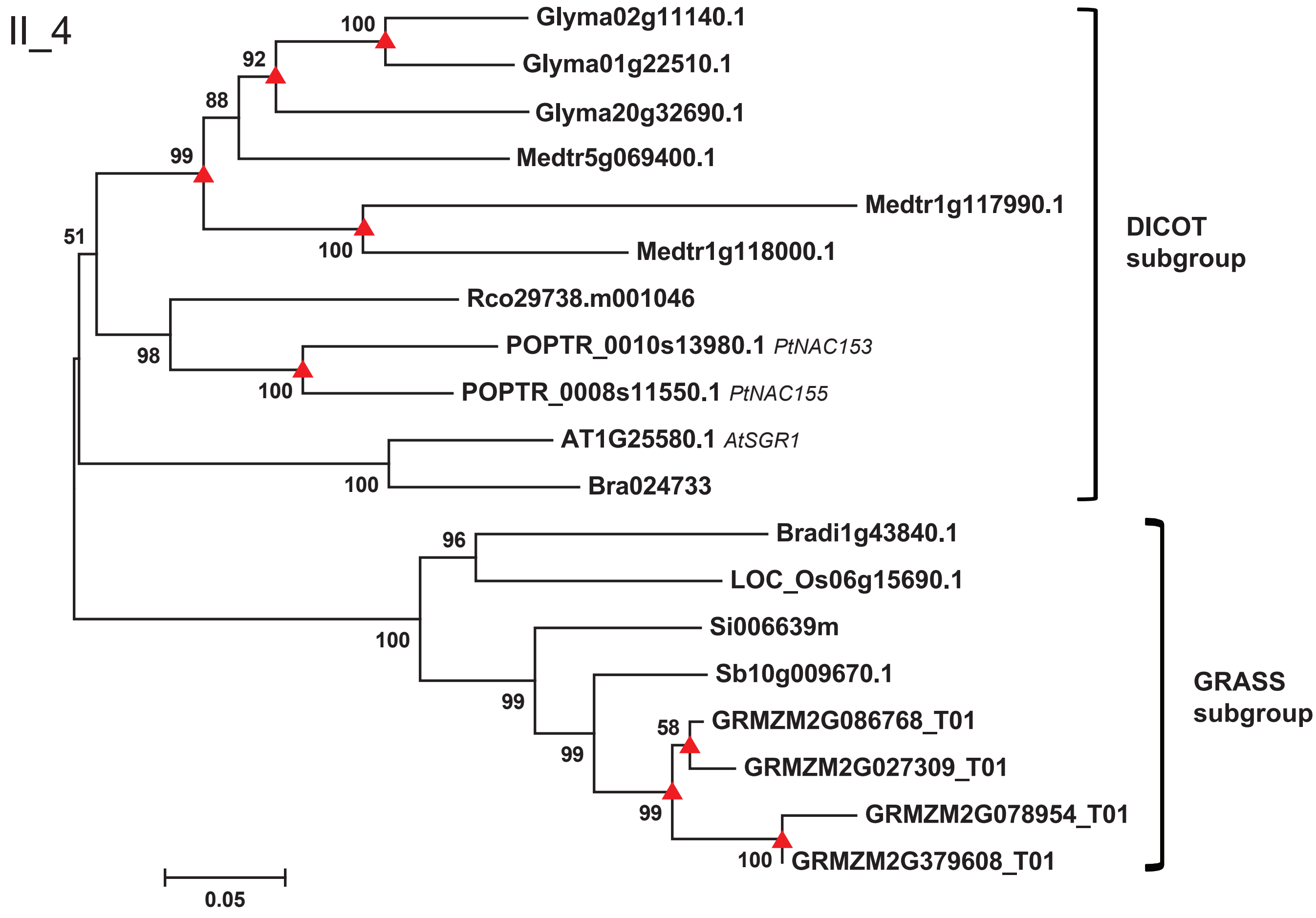
II_4
Glyma02g11140.1
Glyma01g22510.1
Glyma20g32690.1
Medtr5g069400.1
Medtr1g117990.1
Medtr1g118000.1
Rco29738.m001046
POPTR_0010s13980.1 PtNAC153
POPTR_0008s11550.1 PtNAC155
AT1G25580.1 AtSGR1
Bra024733
Bradi1g43840.1
LOC_Os06g15690.1
Si006639m
Sb10g009670.1
GRMZM2G086768_T01
GRMZM2G027309_T01
GRMZM2G078954_T01
GRMZM2G379608_T01
DICOT subgroup
GRASS subgroup
0.05

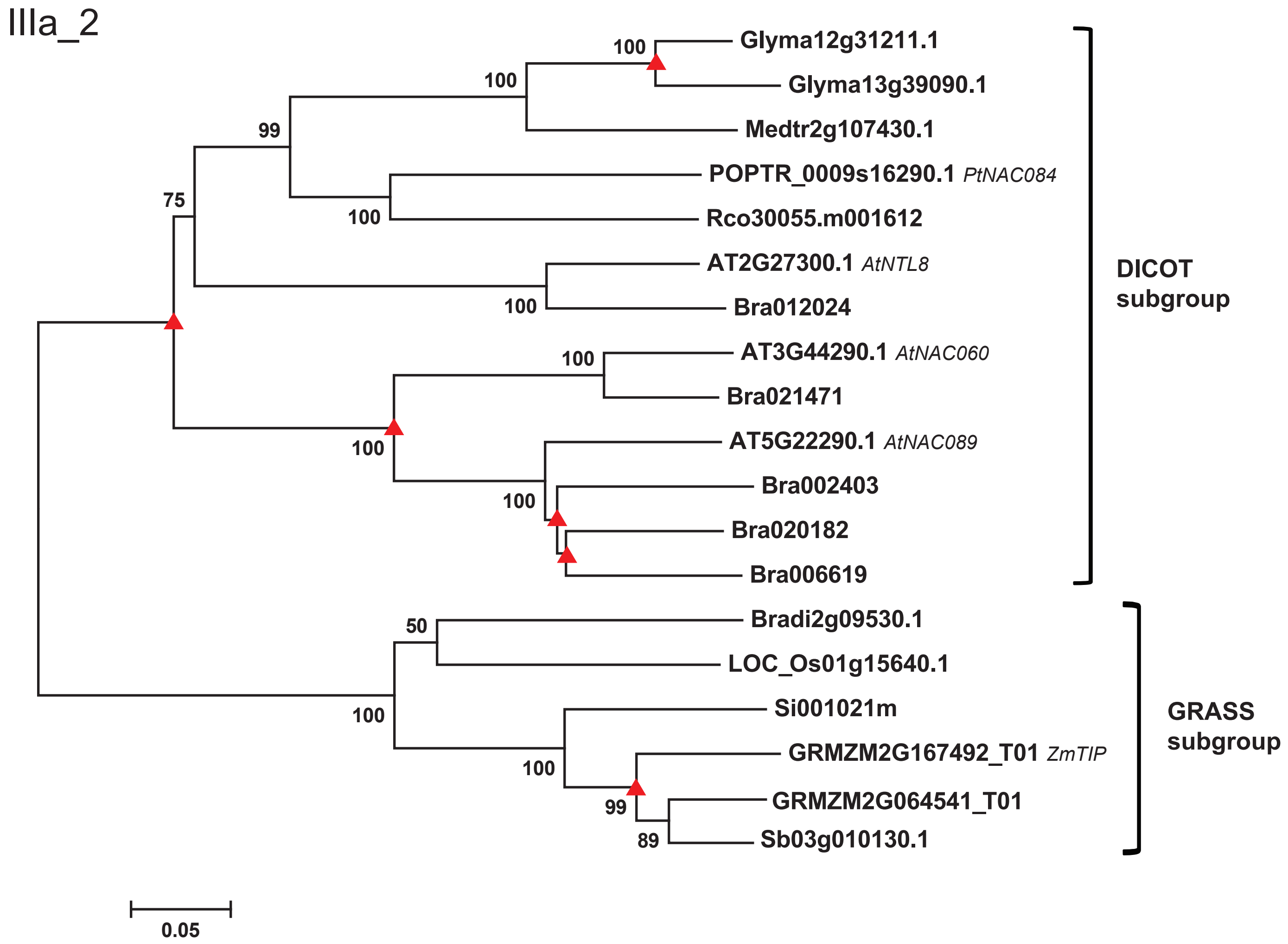
IIIa_2
Glyma12g31211.1
Glyma13g39090.1
Medtr2g107430.1
POPTR_0009s16290.1 PtNAC084
Rco30055.m001612
AT2G27300.1 AtNTL8
Bra012024
AT3G44290.1 AtNAC060
Bra021471
AT5G22290.1 AtNAC089
Bra002403
Bra020182
Bra006619
Bradi2g09530.1
LOC_Os01g15640.1
Si001021m
GRMZM2G167492_T01 ZmTIP
GRMZM2G064541_T01
Sb03g010130.1
DICOT subgroup
GRASS subgroup
0.05

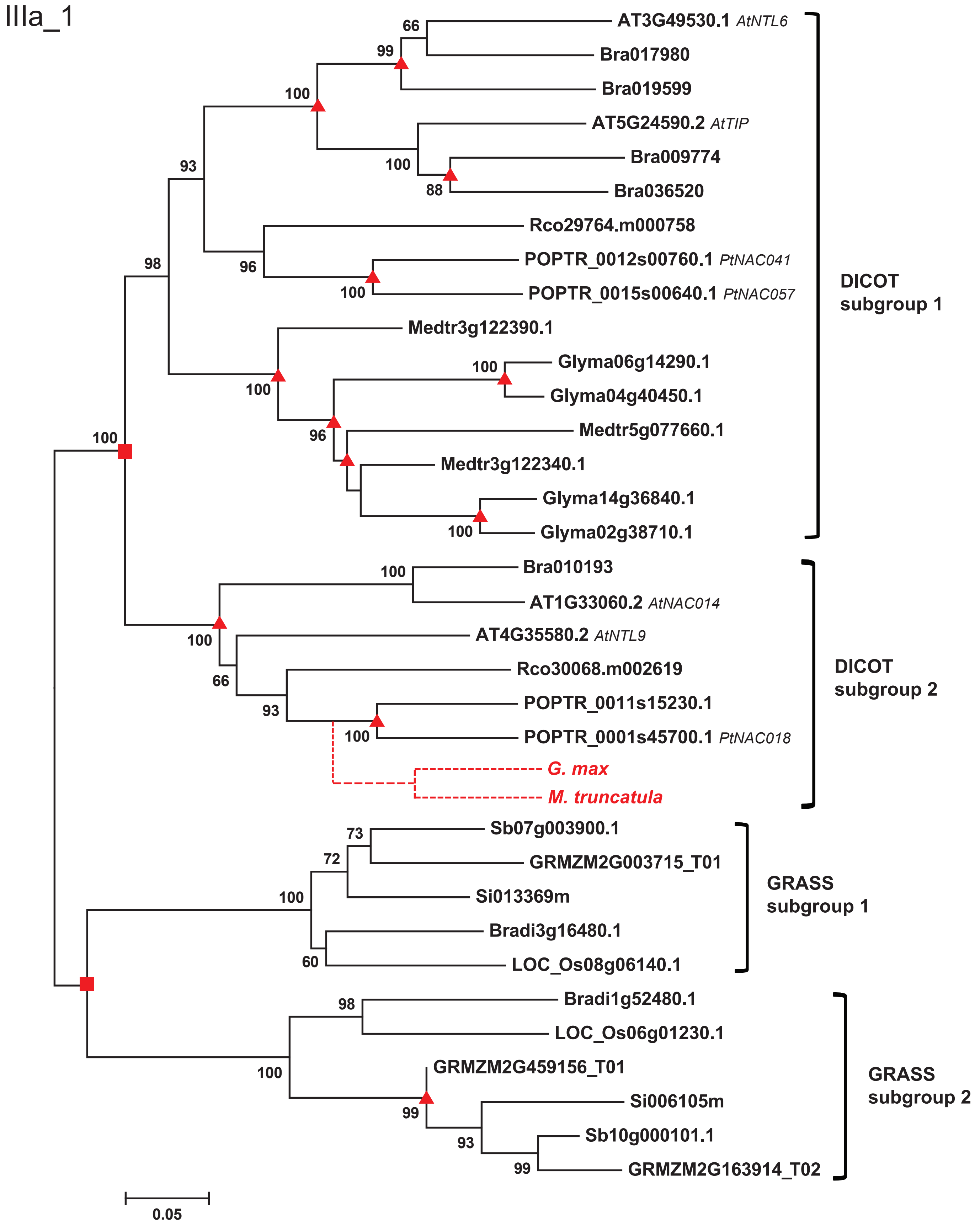

IIIa_1
AT3G49530.1 AtNTL6
Bra017980
Bra019599
AT5G24590.2 AtTIP
Bra009774
Bra036520
Rco29764.m000758
POPTR_0012s00760.1 PtNAC041
POPTR_0015s00640.1 PtNAC057
Medtr3g122390.1
Glyma06g14290.1
Glyma04g40450.1
Medtr5g077660.1
Medtr3g122340.1
Glyma14g36840.1
Glyma02g38710.1
DICOT subgroup 1
Bra010193
AT1G33060.2 AtNAC014
AT4G35580.2 AtNTL9
Rco30068.m002619
POPTR_0011s15230.1
POPTR_0001s45700.1 PtNAC018
G. max
M. truncatula
DICOT subgroup 2
Sb07g003900.1
GRMZM2G003715_T01
Si013369m
Bradi3g16480.1
LOC_Os08g06140.1
GRASS subgroup 1
Bradi1g52480.1
LOC_Os06g01230.1
GRMZM2G459156_T01
Si006105m
Sb10g000101.1
GRMZM2G163914_T02
GRASS subgroup 2
0.05

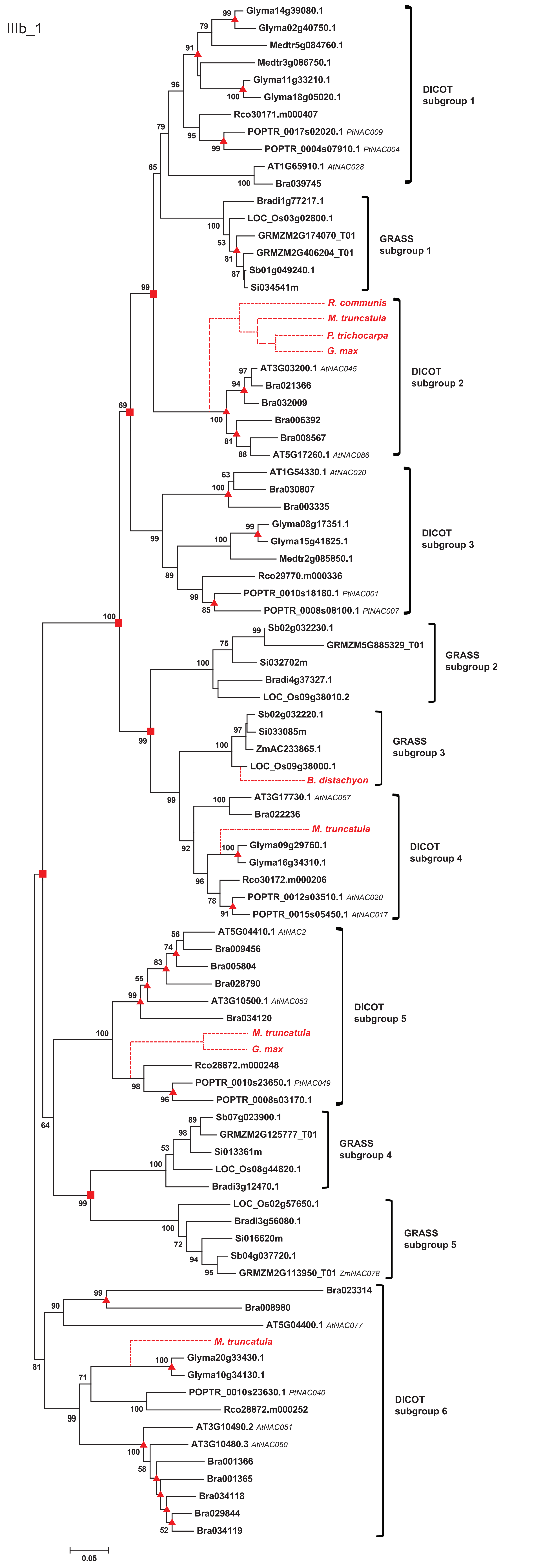
IIIb_1
Glyma14g39080.1
Glyma02g40750.1
Medtr5g084760.1
Medtr3g086750.1
Glyma11g33210.1
Glyma18g05020.1
Rco30171.m000407
POPTR_0017s02020.1 PtNAC009
POPTR_0004s07910.1 PtNAC004
AT1G65910.1 AtNAC028
Bra039745
DICOT subgroup 1
Bradi1g77217.1
LOC_Os03g02800.1
GRMZM2G174070_T01
GRMZM2G406204_T01
Sb01g049240.1
Si034541m
GRASS subgroup 1
R. communis
M. truncatula
P. trichocarpa
G. max
AT3G03200.1 AtNAC045
Bra021366
Bra032009
Bra006392
Bra008567
AT5G17260.1 AtNAC086
DICOT subgroup 2
AT1G54330.1 AtNAC020
Bra030807
Bra003335
Glyma08g17351.1
Glyma15g41825.1
Medtr2g085850.1
Rco29770.m000336
POPTR_0010s18180.1 PtNAC001
POPTR_0008s08100.1 PtNAC007
DICOT subgroup 3
Sb02g032230.1
GRMZM5G885329_T01
Si032702m
Bradi4g37327.1
LOC_Os09g38010.2
GRASS subgroup 2
Sb02g032220.1
Si033085m
ZmAC233865.1
LOC_Os09g38000.1
B. distachyon
GRASS subgroup 3
AT3G17730.1 AtNAC057
Bra022236
M. truncatula
Glyma09g29760.1
Glyma16g34310.1
Rco30172.m000206
POPTR_0012s03510.1 AtNAC020
POPTR_0015s05450.1 AtNAC017
DICOT subgroup 4
AT5G04410.1 AtNAC2
Bra009456
Bra005804
Bra028790
AT3G10500.1 AtNAC053
Bra034120
M. truncatula
G. max
Rco28872.m000248
POPTR_0010s23650.1 PtNAC049
POPTR_0008s03170.1
DICOT subgroup 5
Sb07g023900.1
GRMZM2G125777_T01
Si013361m
LOC_Os08g44820.1
Bradi3g12470.1
GRASS subgroup 4
LOC_Os02g57650.1
Bradi3g56080.1
Si016620m
Sb04g037720.1
GRMZM2G113950_T01 ZmNAC078
GRASS subgroup 5
Bra023314
Bra008980
AT5G04400.1 AtNAC077
M. truncatula
Glyma20g33430.1
Glyma10g34130.1
POPTR_0010s23630.1 PtNAC040
Rco28872.m000252
AT3G10490.2 AtNAC051
AT3G10480.3 AtNAC050
Bra001366
Bra001365
Bra034118
Bra029844
Bra034119
DICOT subgroup 6
0.05

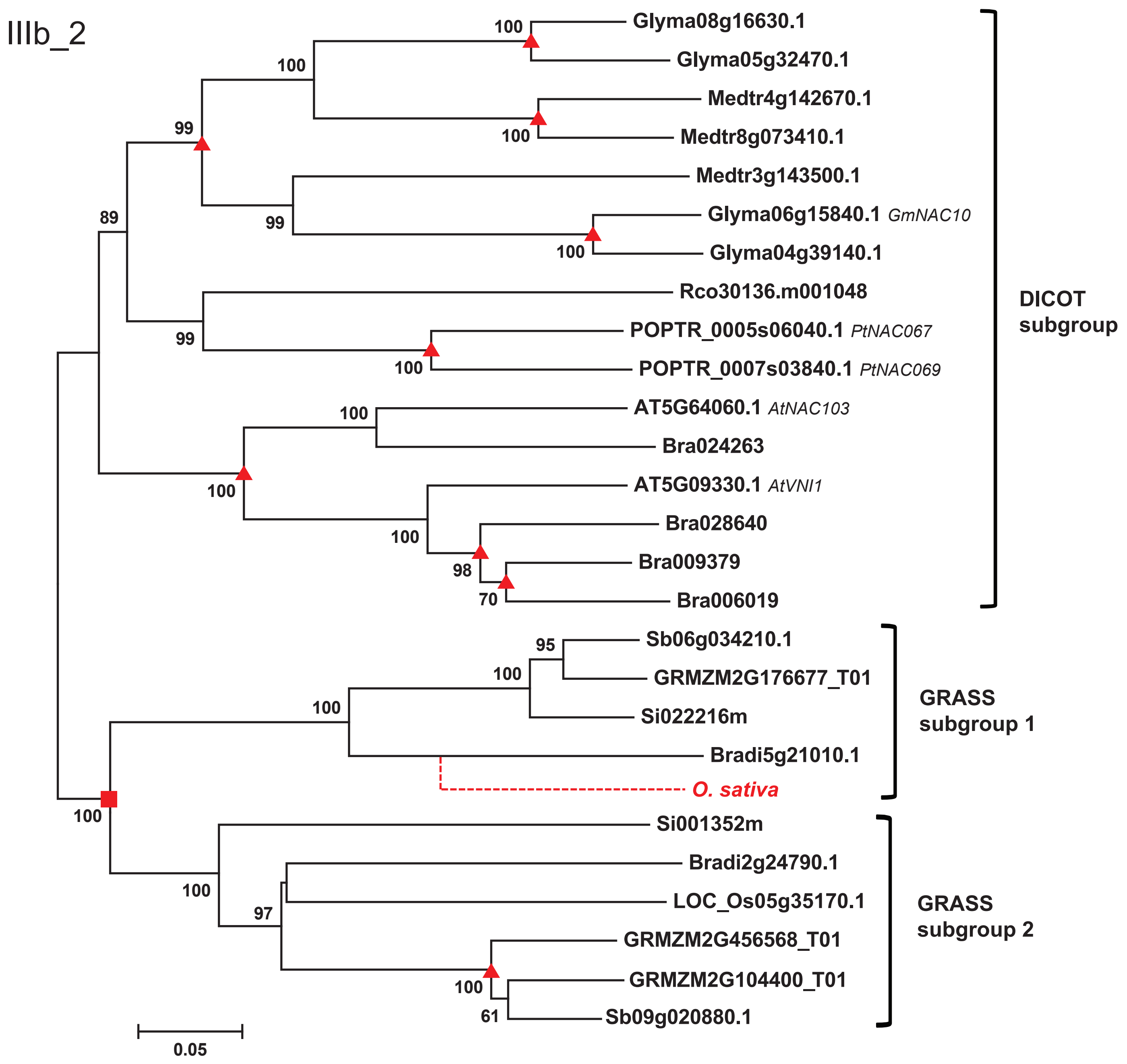
IIIb_2
Glyma08g16630.1
Glyma05g32470.1
Medtr4g142670.1
Medtr8g073410.1
Medtr3g143500.1
Glyma06g15840.1 GmNAC10
Glyma04g39140.1
Rco30136.m001048
POPTR_0005s06040.1 PtNAC067
POPTR_0007s03840.1 PtNAC069
AT5G64060.1 AtNAC103
Bra024263
AT5G09330.1 AtVNI1
Bra028640
Bra009379
Bra006019
Sb06g034210.1
GRMZM2G176677_T01
Si022216m
Bradi5g21010.1
O. sativa
Si001352m
Bradi2g24790.1
LOC_Os05g35170.1
GRMZM2G456568_T01
GRMZM2G104400_T01
Sb09g020880.1
DICOT subgroup
GRASS subgroup 1
GRASS subgroup 2
100
100
100
99
99
100
89
99
100
100
100
100
98
70
95
100
100
100
100
97
100
61
0.05

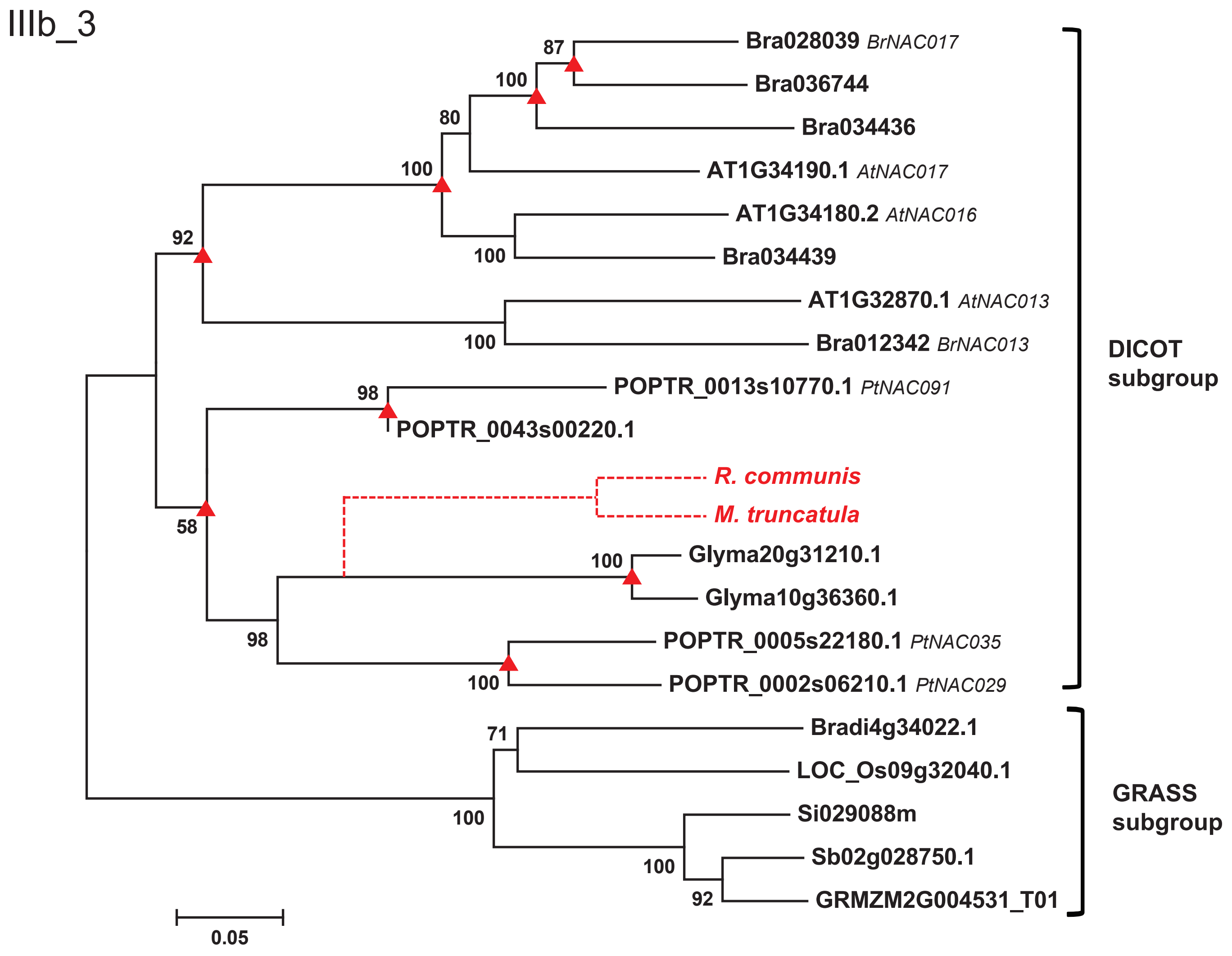
IIIb_3
Bra028039 BrNAC017
Bra036744
Bra034436
AT1G34190.1 AtNAC017
AT1G34180.2 AtNAC016
Bra034439
AT1G32870.1 AtNAC013
Bra012342 BrNAC013
POPTR_0013s10770.1 PtNAC091
POPTR_0043s00220.1
R. communis
M. truncatula
Glyma20g31210.1
Glyma10g36360.1
POPTR_0005s22180.1 PtNAC035
POPTR_0002s06210.1 PtNAC029
Bradi4g34022.1
LOC_Os09g32040.1
Si029088m
Sb02g028750.1
GRMZM2G004531_T01
DICOT subgroup
GRASS subgroup
87
100
80
100
100
92
100
98
58
100
98
100
71
100
100
92
0.05

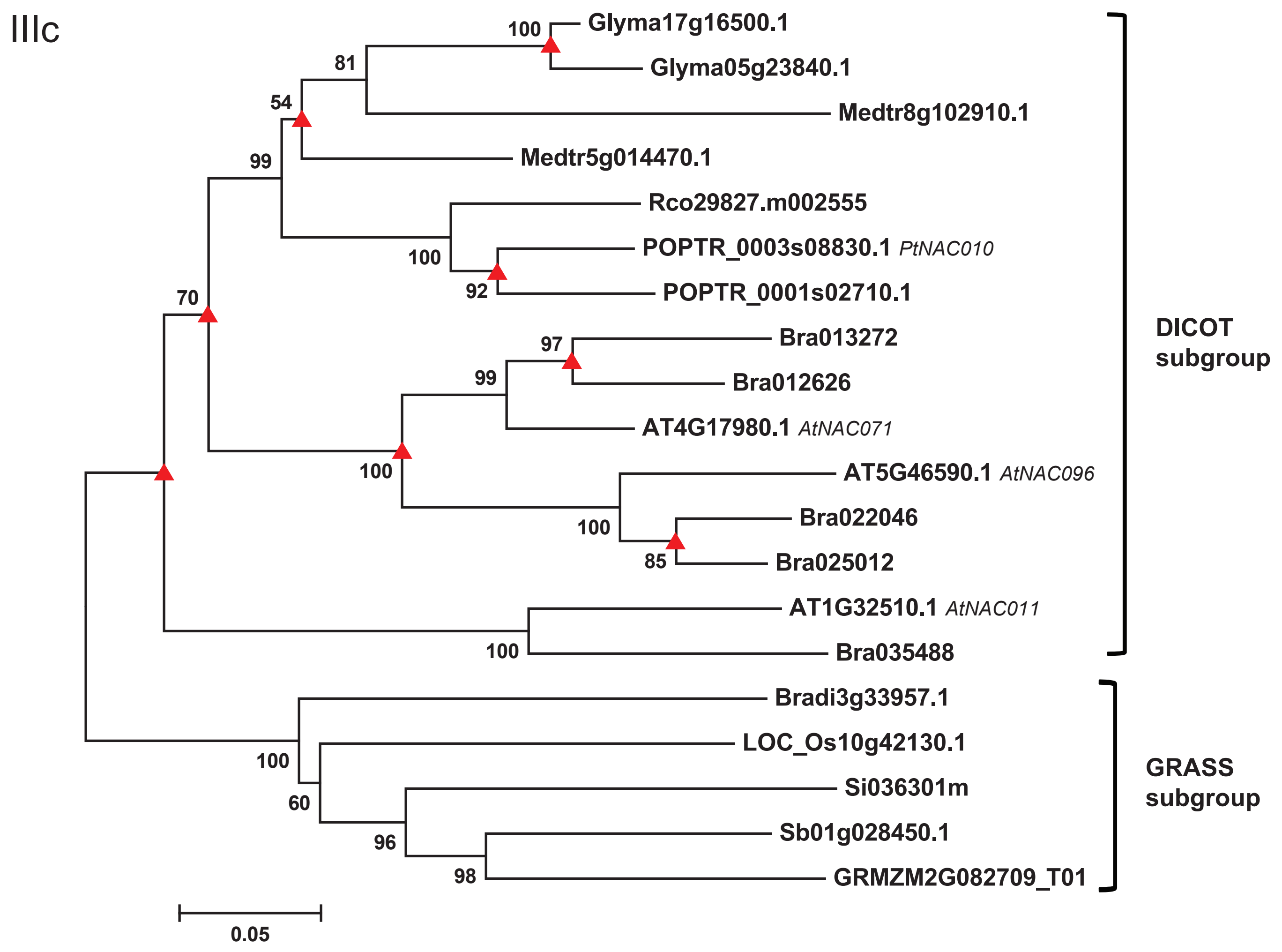
IIIc
Glyma17g16500.1
Glyma05g23840.1
Medtr8g102910.1
Medtr5g014470.1
Rco29827.m002555
POPTR_0003s08830.1 PtNAC010
POPTR_0001s02710.1
Bra013272
Bra012626
AT4G17980.1 AtNAC071
AT5G46590.1 AtNAC096
Bra022046
Bra025012
AT1G32510.1 AtNAC011
Bra035488
Bradi3g33957.1
LOC_Os10g42130.1
Si036301m
Sb01g028450.1
GRMZM2G082709_T01
DICOT subgroup
GRASS subgroup
100
81
54
99
100
92
70
97
99
100
100
85
100
100
60
96
98
0.05

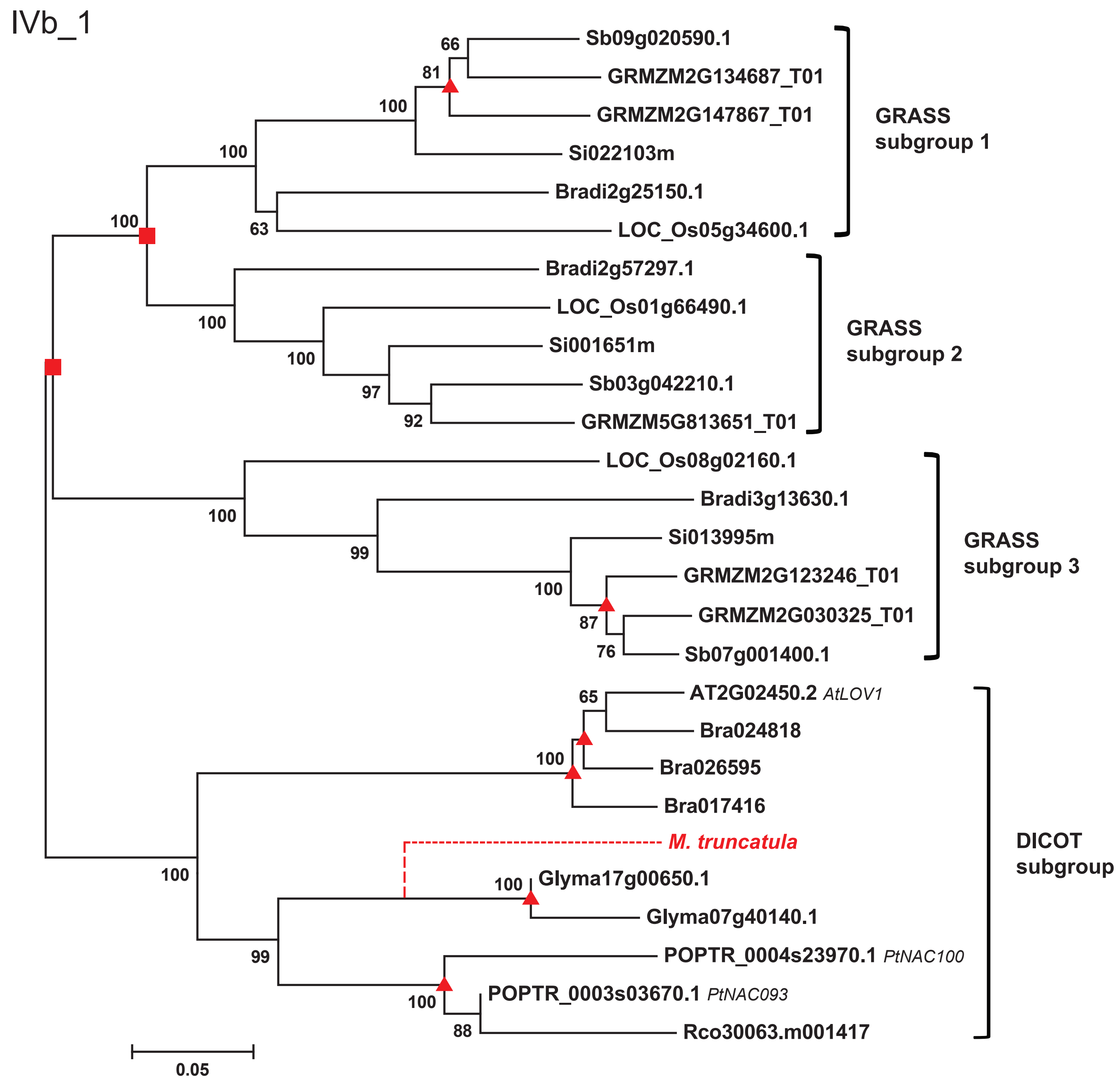
IVb_1
Sb09g020590.1
GRMZM2G134687_T01
GRMZM2G147867_T01
Si022103m
Bradi2g25150.1
LOC_Os05g34600.1
Bradi2g57297.1
LOC_Os01g66490.1
Si001651m
Sb03g042210.1
GRMZM5G813651_T01
LOC_Os08g02160.1
Bradi3g13630.1
Si013995m
GRMZM2G123246_T01
GRMZM2G030325_T01
Sb07g001400.1
AT2G02450.2 AtLOV1
Bra024818
Bra026595
Bra017416
M. truncatula
Glyma17g00650.1
Glyma07g40140.1
POPTR_0004s23970.1 PtNAC100
POPTR_0003s03670.1 PtNAC093
Rco30063.m001417
GRASS subgroup 1
GRASS subgroup 2
GRASS subgroup 3
DICOT subgroup
66
81
100
100
63
100
100
100
97
92
100
99
100
87
76
65
100
100
100
99
100
88
0.05

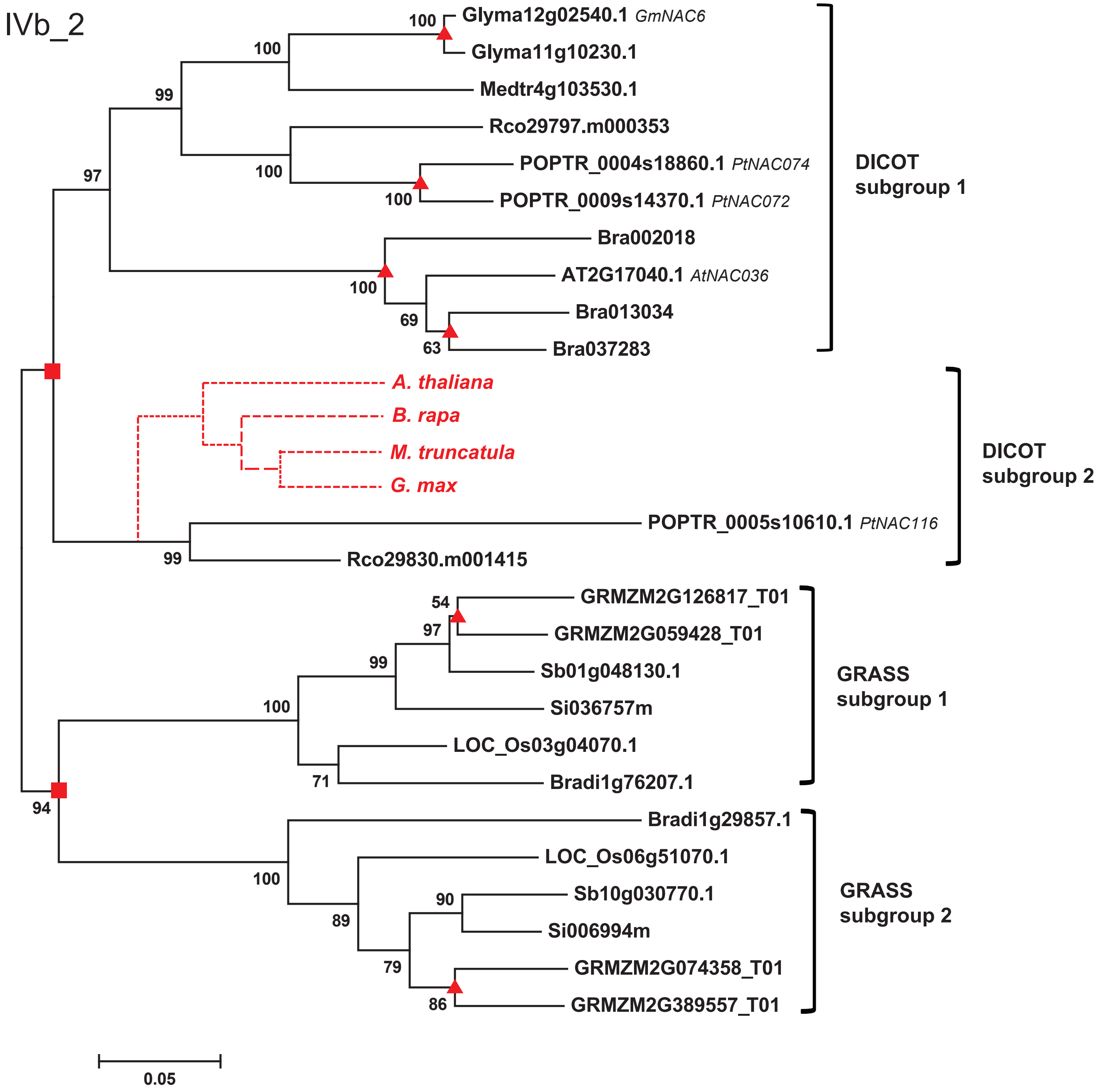

IVb_2
Glyma12g02540.1 GmNAC6
Glyma11g10230.1
Medtr4g103530.1
Rco29797.m000353
POPTR_0004s18860.1 PtNAC074
POPTR_0009s14370.1 PtNAC072
Bra002018
AT2G17040.1 AtNAC036
Bra013034
Bra037283
DICOT subgroup 1
A. thaliana
B. rapa
M. truncatula
G. max
POPTR_0005s10610.1 PtNAC116
Rco29830.m001415
DICOT subgroup 2
GRMZM2G126817_T01
GRMZM2G059428_T01
Sb01g048130.1
Si036757m
LOC_Os03g04070.1
Bradi1g76207.1
GRASS subgroup 1
Bradi1g29857.1
LOC_Os06g51070.1
Sb10g030770.1
Si006994m
GRMZM2G074358_T01
GRMZM2G389557_T01
GRASS subgroup 2
0.05

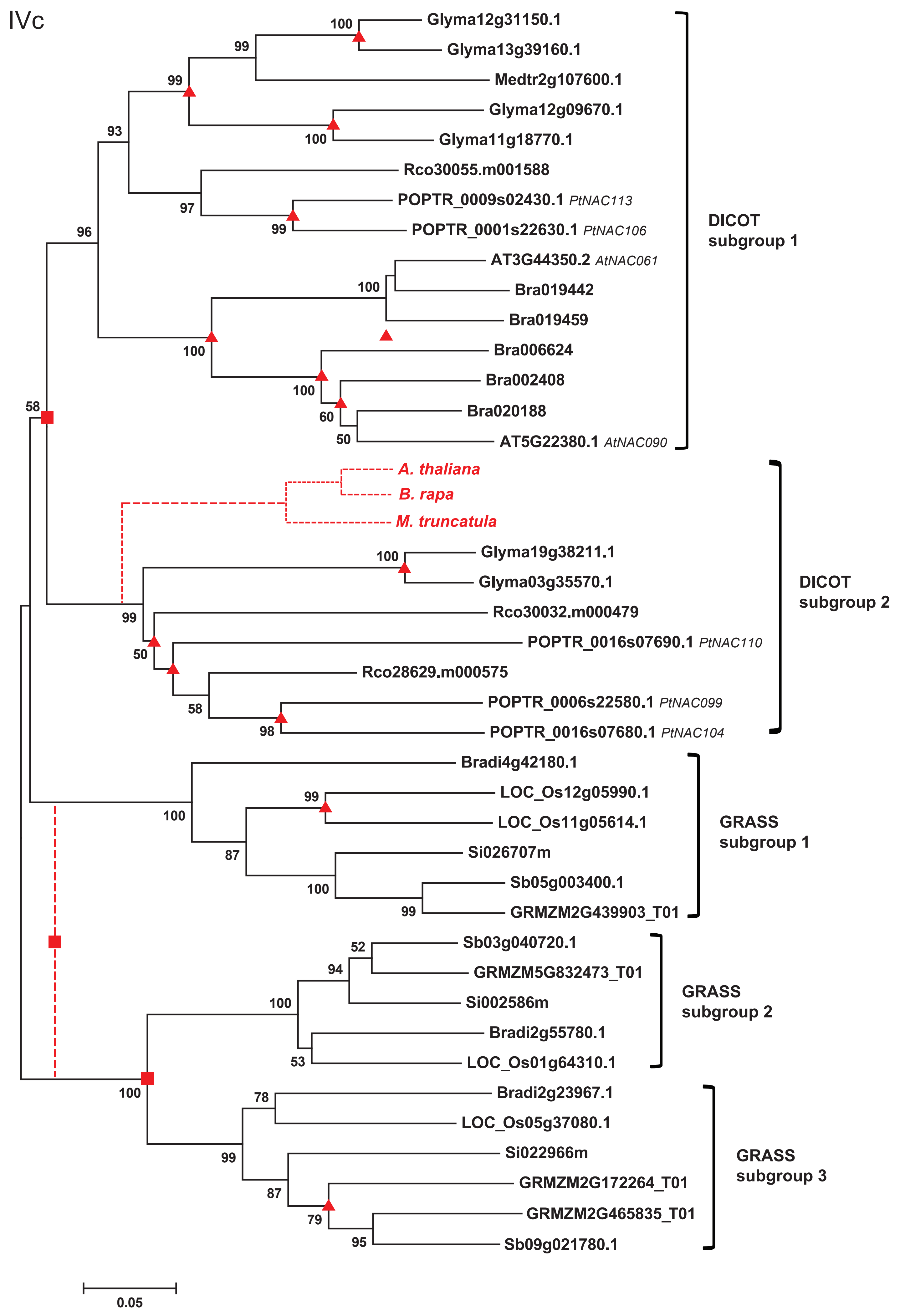
IVc
Glyma12g31150.1
Glyma13g39160.1
Medtr2g107600.1
Glyma12g09670.1
Glyma11g18770.1
Rco30055.m001588
POPTR_0009s02430.1 PtNAC113
POPTR_0001s22630.1 PtNAC106
AT3G44350.2 AtNAC061
Bra019442
Bra019459
Bra006624
Bra002408
Bra020188
AT5G22380.1 AtNAC090
DICOT subgroup 1
A. thaliana
B. rapa
M. truncatula
Glyma19g38211.1
Glyma03g35570.1
Rco30032.m000479
POPTR_0016s07690.1 PtNAC110
Rco28629.m000575
POPTR_0006s22580.1 PtNAC099
POPTR_0016s07680.1 PtNAC104
DICOT subgroup 2
Bradi4g42180.1
LOC_Os12g05990.1
LOC_Os11g05614.1
Si026707m
Sb05g003400.1
GRMZM2G439903_T01
GRASS subgroup 1
Sb03g040720.1
GRMZM5G832473_T01
Si002586m
Bradi2g55780.1
LOC_Os01g64310.1
GRASS subgroup 2
Bradi2g23967.1
LOC_Os05g37080.1
Si022966m
GRMZM2G172264_T01
GRMZM2G465835_T01
Sb09g021780.1
GRASS subgroup 3
0.05

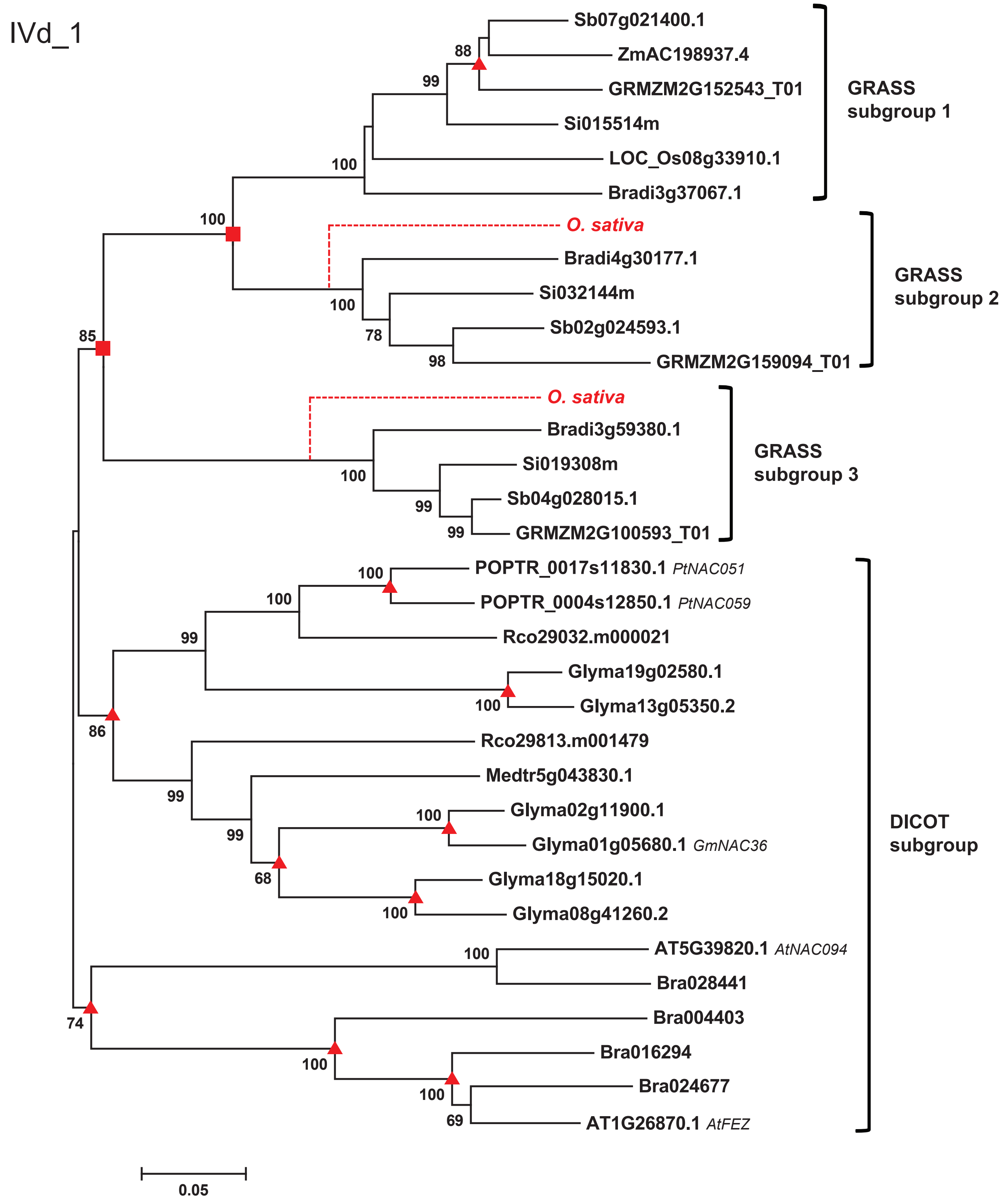
IVd_1
Sb07g021400.1
ZmAC198937.4
GRMZM2G152543_T01
Si015514m
LOC_Os08g33910.1
Bradi3g37067.1
GRASS subgroup 1
O. sativa
Bradi4g30177.1
Si032144m
Sb02g024593.1
GRMZM2G159094_T01
GRASS subgroup 2
O. sativa
Bradi3g59380.1
Si019308m
Sb04g028015.1
GRMZM2G100593_T01
GRASS subgroup 3
POPTR_0017s11830.1 PtNAC051
POPTR_0004s12850.1 PtNAC059
Rco29032.m000021
Glyma19g02580.1
Glyma13g05350.2
Rco29813.m001479
Medtr5g043830.1
Glyma02g11900.1
Glyma01g05680.1 GmNAC36
Glyma18g15020.1
Glyma08g41260.2
AT5G39820.1 AtNAC094
Bra028441
Bra004403
Bra016294
Bra024677
AT1G26870.1 AtFEZ
DICOT subgroup
88
99
100
100
100
100
78
98
85
100
99
99
100
100
99
100
86
99
99
100
68
100
100
74
100
100
69
0.05

IVd_2

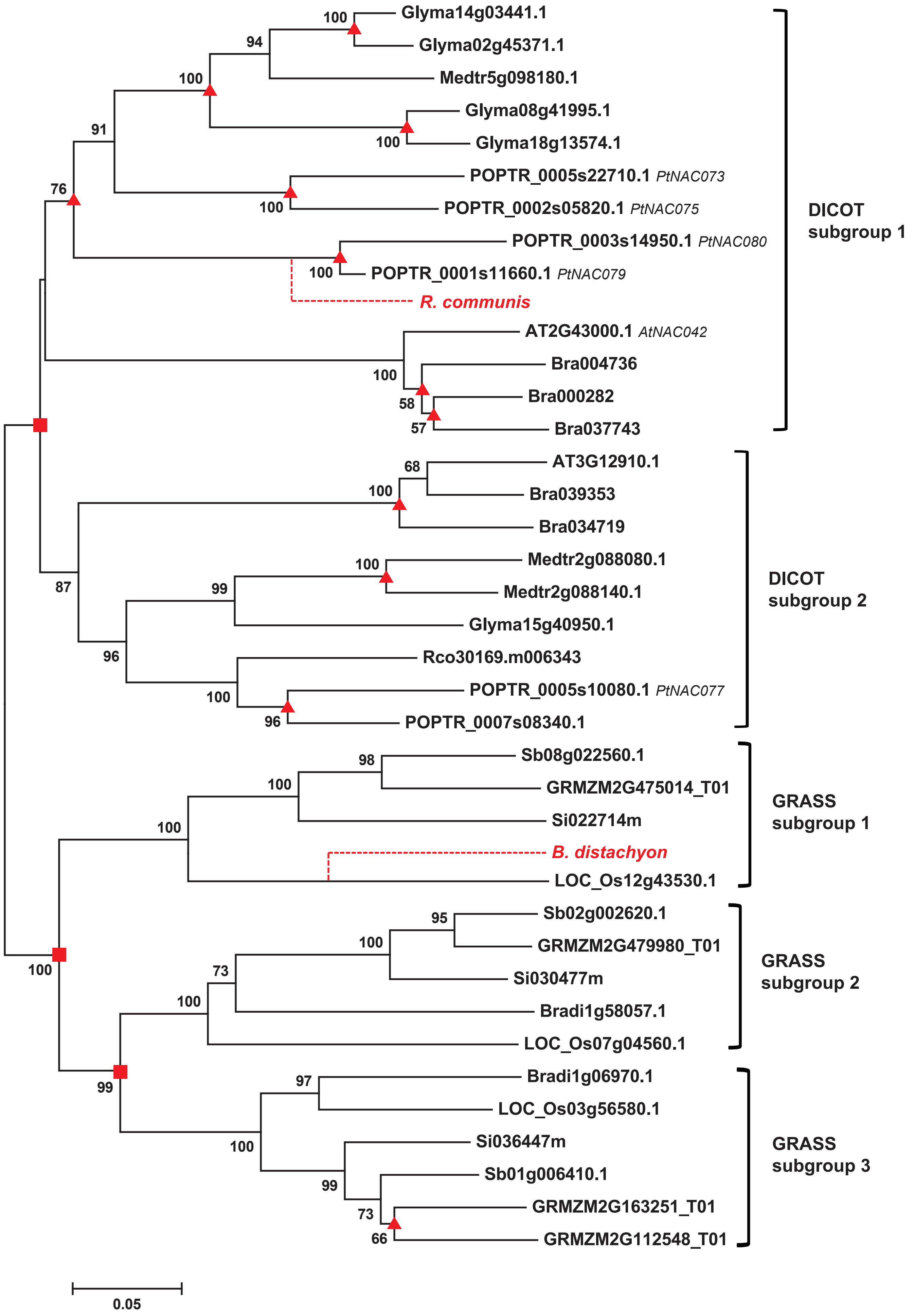
Glyma14g03441.1
Glyma02g45371.1
Medtr5g098180.1
Glyma08g41995.1
Glyma18g13574.1
POPTR_0005s22710.1 PtNAC073
POPTR_0002s05820.1 PtNAC075
POPTR_0003s14950.1 PtNAC080
POPTR_0001s11660.1 PtNAC079
R. communis
AT2G43000.1 AtNAC042
Bra004736
Bra000282
Bra037743
DICOT subgroup 1
AT3G12910.1
Bra039353
Bra034719
Medtr2g088080.1
Medtr2g088140.1
Glyma15g40950.1
Rco30169.m006343
POPTR_0005s10080.1 PtNAC077
POPTR_0007s08340.1
DICOT subgroup 2
Sb08g022560.1
GRMZM2G475014_T01
Si022714m
B. distachyon
LOC_Os12g43530.1
GRASS subgroup 1
Sb02g002620.1
GRMZM2G479980_T01
Si030477m
Bradi1g58057.1
LOC_Os07g04560.1
GRASS subgroup 2
Bradi1g06970.1
LOC_Os03g56580.1
Si036447m
Sb01g006410.1
GRMZM2G163251_T01
GRMZM2G112548_T01
GRASS subgroup 3
0.05

Va_1

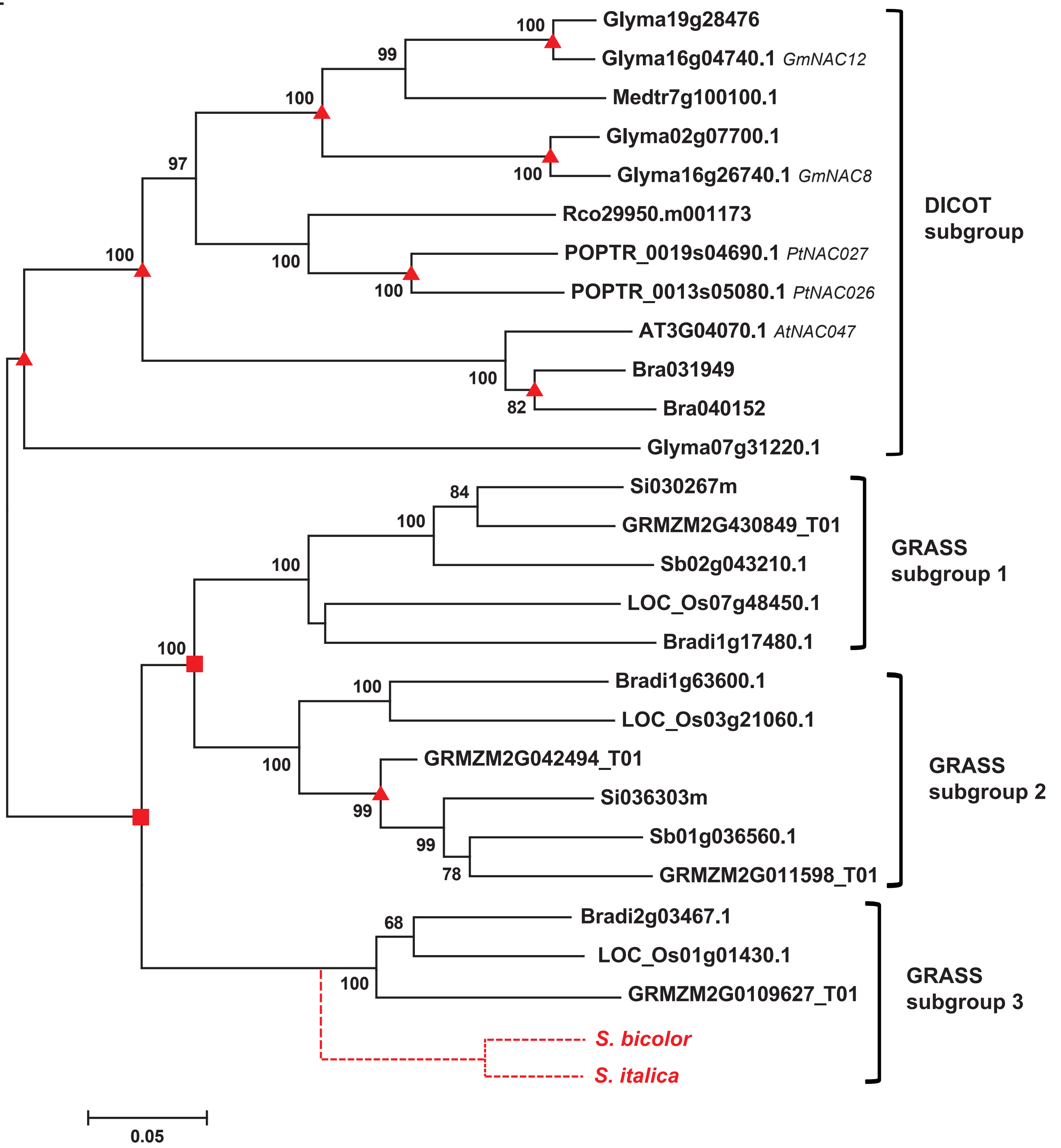

Glyma19g28476
Glyma16g04740.1 GmNAC12
Medtr7g100100.1
Glyma02g07700.1
Glyma16g26740.1 GmNAC8
Rco29950.m001173
POPTR_0019s04690.1 PtNAC027
POPTR_0013s05080.1 PtNAC026
AT3G04070.1 AtNAC047
Bra031949
Bra040152
Glyma07g31220.1
DICOT subgroup
Si030267m
GRMZM2G430849_T01
Sb02g043210.1
LOC_Os07g48450.1
Bradi1g17480.1
GRASS subgroup 1
Bradi1g63600.1
LOC_Os03g21060.1
GRMZM2G042494_T01
Si036303m
Sb01g036560.1
GRMZM2G011598_T01
GRASS subgroup 2
Bradi2g03467.1
LOC_Os01g01430.1
GRMZM2G0109627_T01
S. bicolor
S. italica
GRASS subgroup 3
100
99
100
97
100
100
100
100
100
82
84
100
100
100
100
100
99
99
78
68
100
0.05

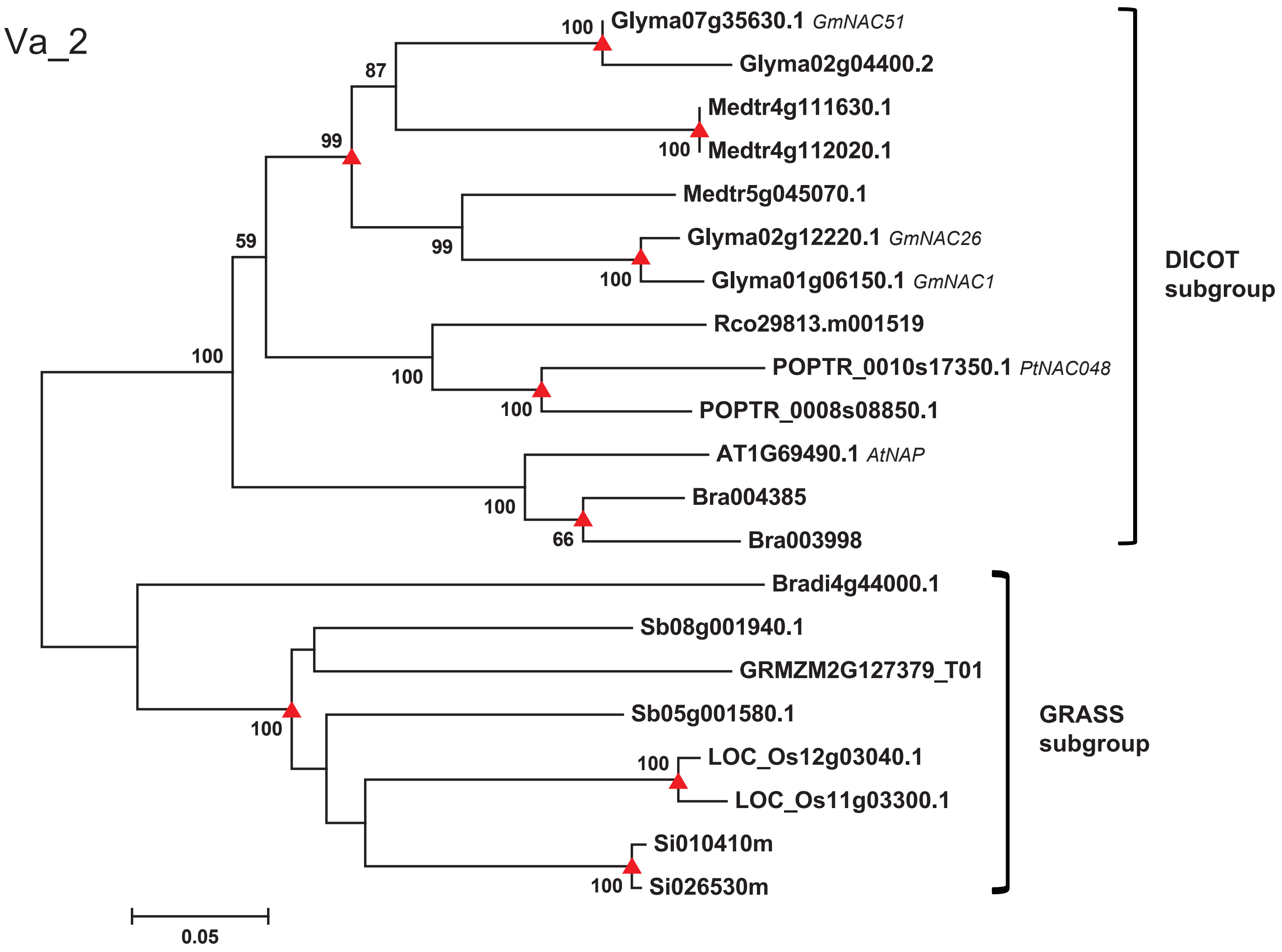
Va_2
Glyma07g35630.1 GmNAC51
Glyma02g04400.2
Medtr4g111630.1
Medtr4g112020.1
Medtr5g045070.1
Glyma02g12220.1 GmNAC26
Glyma01g06150.1 GmNAC1
Rco29813.m001519
POPTR_0010s17350.1 PtNAC048
POPTR_0008s08850.1
AT1G69490.1 AtNAP
Bra004385
Bra003998
Bradi4g44000.1
Sb08g001940.1
GRMZM2G127379_T01
Sb05g001580.1
LOC_Os12g03040.1
LOC_Os11g03300.1
Si010410m
Si026530m
DICOT subgroup
GRASS subgroup
100
87
99
100
99
100
59
100
100
100
100
100
66
100
100
100
0.05

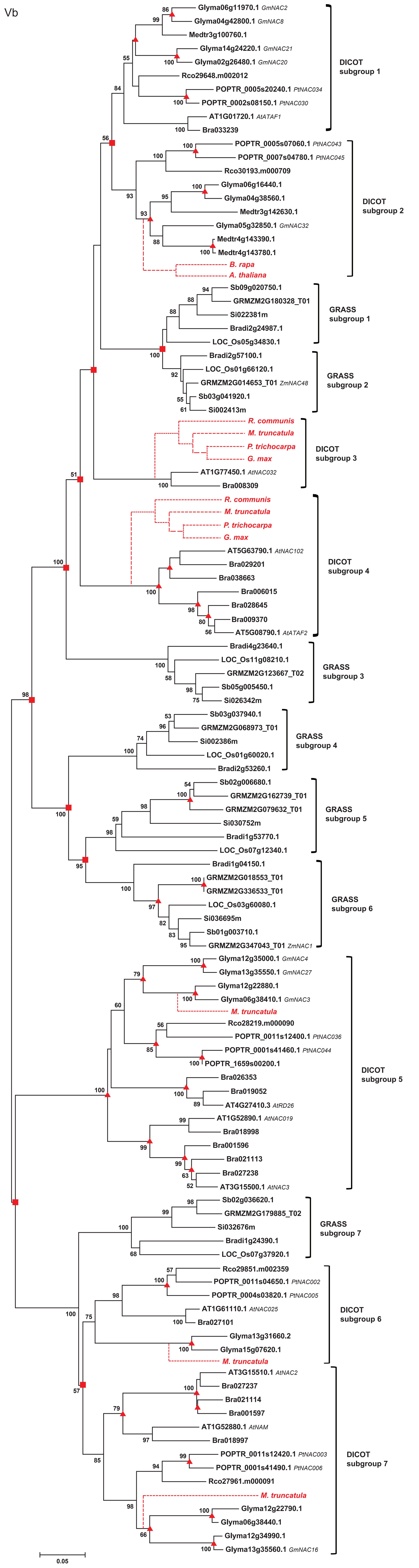

Vb
Glyma06g11970.1 GmNAC2
Glyma04g42800.1 GmNAC8
Medtr3g100760.1
Glyma14g24220.1 GmNAC21
Glyma02g26480.1 GmNAC20
Rco29648.m002012
POPTR_0005s20240.1 PtNAC034
POPTR_0002s08150.1 PtNAC030
AT1G01720.1 AtATAF1
Bra033239
DICOT subgroup 1
POPTR_0005s07060.1 PtNAC043
POPTR_0007s04780.1 PtNAC045
Rco30193.m000709
Glyma06g16440.1
Glyma04g38560.1
Medtr3g142630.1
Glyma05g32850.1 GmNAC32
Medtr4g143390.1
Medtr4g143780.1
B. rapa
A. thaliana
DICOT subgroup 2
Sb09g020750.1
GRMZM2G180328_T01
Si022381m
Bradi2g24987.1
LOC_Os05g34830.1
GRASS subgroup 1
Bradi2g57100.1
LOC_Os01g66120.1
GRMZM2G014653_T01 ZmNAC48
Sb03g041920.1
Si002413m
GRASS subgroup 2
R. communis
M. truncatula
P. trichocarpa
G. max
AT1G77450.1 AtNAC032
Bra008309
DICOT subgroup 3
R. communis
M. truncatula
P. trichocarpa
G. max
AT5G63790.1 AtNAC102
Bra029201
Bra038663
Bra006015
Bra028645
Bra009370
AT5G08790.1 AtATAF2
DICOT subgroup 4
Bradi4g23640.1
LOC_Os11g08210.1
GRMZM2G123667_T02
Sb05g005450.1
Si026342m
GRASS subgroup 3
Sb03g037940.1
GRMZM2G068973_T01
Si002386m
LOC_Os01g60020.1
Bradi2g53260.1
GRASS subgroup 4
Sb02g006680.1
GRMZM2G162739_T01
GRMZM2G079632_T01
Si030752m
Bradi1g53770.1
LOC_Os07g12340.1
GRASS subgroup 5
Bradi1g04150.1
GRMZM2G018553_T01
GRMZM2G336533_T01
LOC_Os03g60080.1
Si036695m
Sb01g003710.1
GRMZM2G347043_T01 ZmNAC1
GRASS subgroup 6
Glyma12g35000.1 GmNAC4
Glyma13g35550.1 GmNAC27
Glyma12g22880.1
Glyma06g38410.1 GmNAC3
M. truncatula
Rco28219.m000090
POPTR_0011s12400.1 PtNAC036
POPTR_0001s41460.1 PtNAC044
POPTR_1659s00200.1
Bra026353
Bra019052
AT4G27410.3 AtRD26
AT1G52890.1 AtNAC019
Bra018998
Bra001596
Bra021113
Bra027238
AT3G15500.1 AtNAC3
DICOT subgroup 5
Sb02g036620.1
GRMZM2G179885_T02
Si032676m
Bradi1g24390.1
LOC_Os07g37920.1
GRASS subgroup 7
Rco29851.m002359
POPTR_0011s04650.1 PtNAC002
POPTR_0004s03820.1 PtNAC005
AT1G61110.1 AtNAC025
Bra027101
Glyma13g31660.2
Glyma15g07620.1
M. truncatula
DICOT subgroup 6
AT3G15510.1 AtNAC2
Bra027237
Bra021114
Bra001597
AT1G52880.1 AtNAM
Bra018997
POPTR_0011s12420.1 PtNAC003
POPTR_0001s41490.1 PtNAC006
Rco27961.m000091
M. truncatula
Glyma12g22790.1
Glyma06g38440.1
Glyma12g34990.1
Glyma13g35560.1 GmNAC16
DICOT subgroup 7
0.05

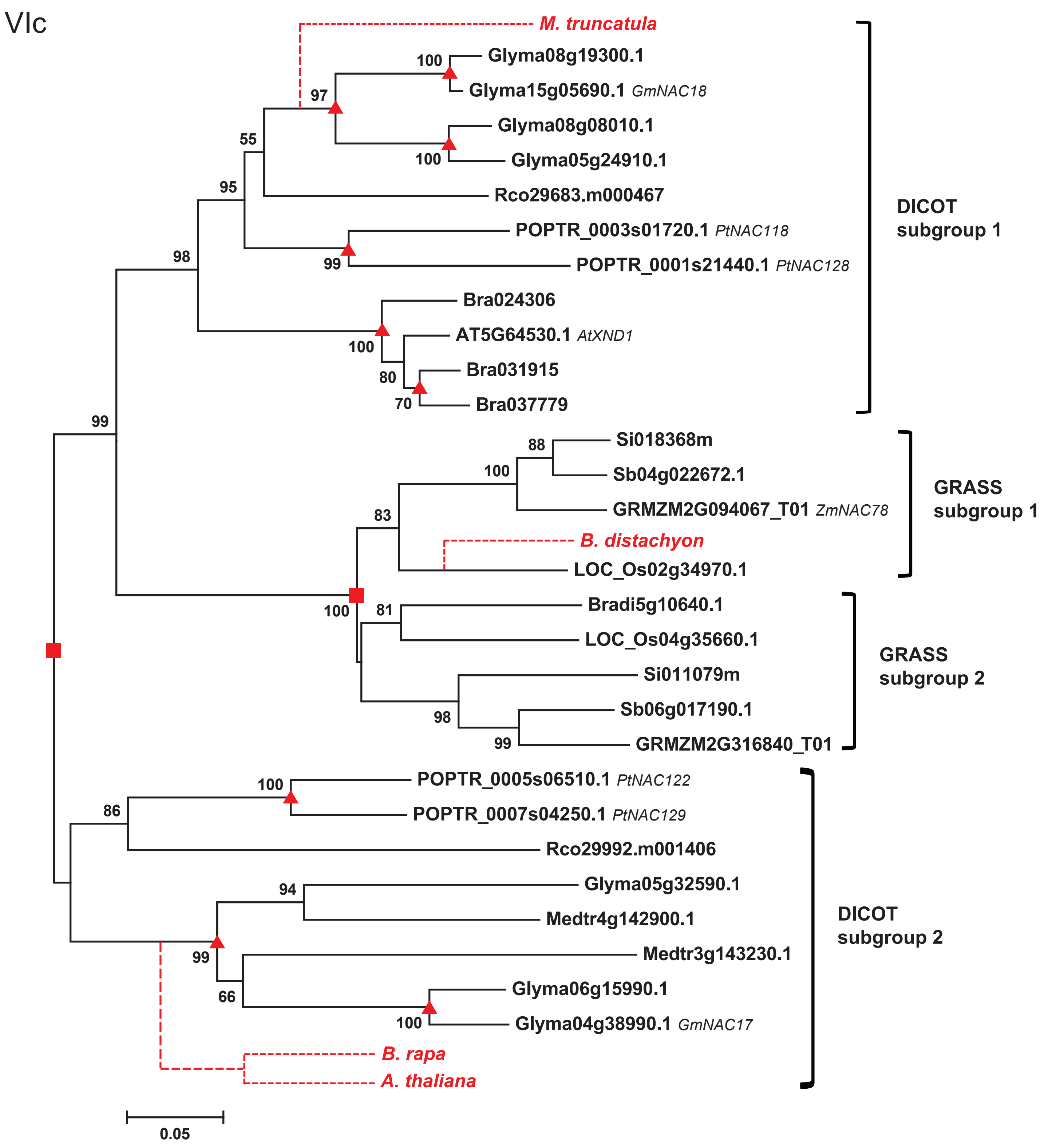

Figure S2. Phylogenetic relationship of sequences within each COGs considered in this study by NJ method with bootstrap support above 50% shown at the nodes. Square boxes indicate ancient duplication events; the triangles indicate recently occurred species-specific duplication events; and the broken lines indicate absent genes, either lost from those species or not yet sequenced. Gene names, based on previous references, are listed after the gene IDs.
