# Supplementary material for: Divergent Evolutionary Patterns of NAC Transcription Factors Are Associated with Diversification and Gene Duplications in Angiosperm
Source: Front Plant Sci. 2017 Jun 30;8:1156. doi: 10.3389/fpls.2017.01156 (PMC5492850; doi:10.3389/fpls.2017.01156)
Supplement: Supplementary file 4 [file Presentation3.PDF]

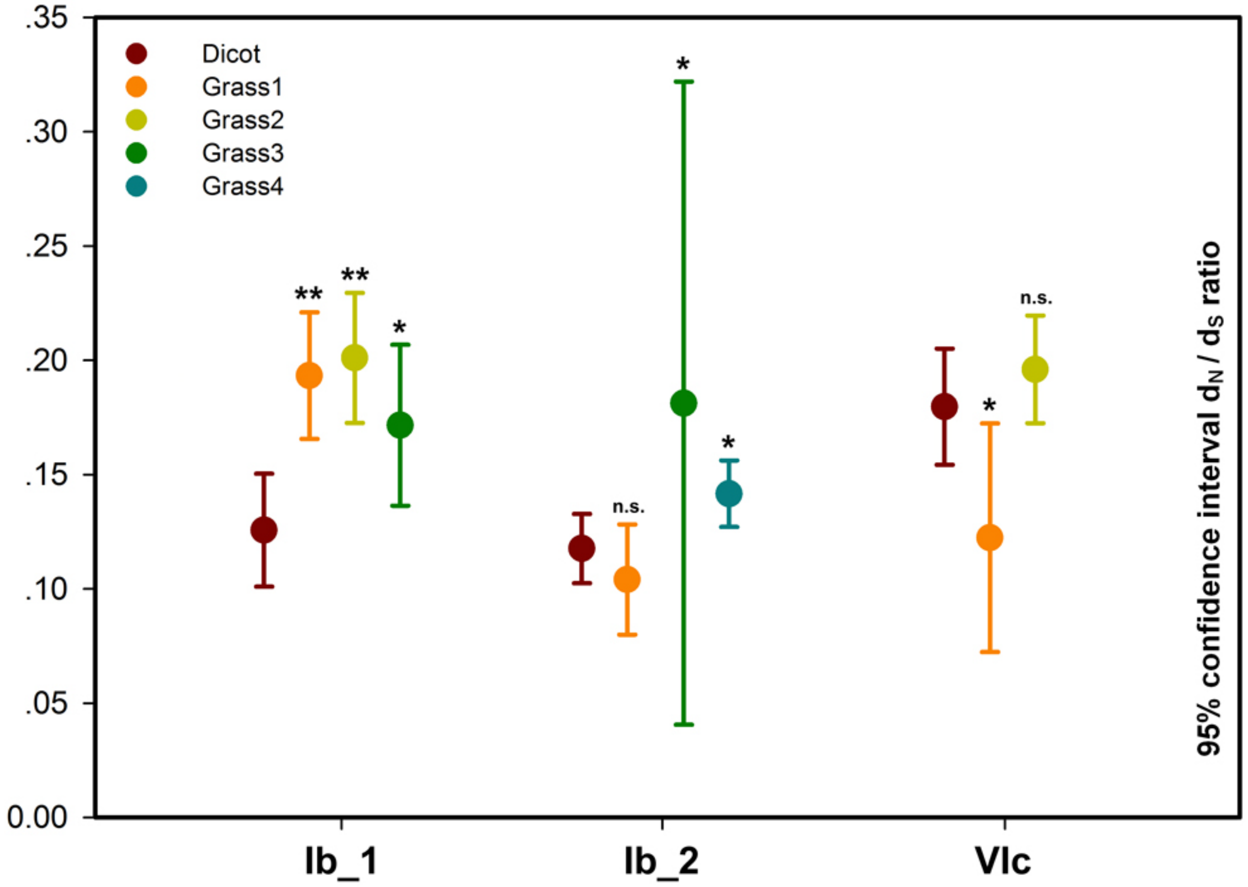

.35
.30
.25
.20
.15
.10
.05
0.00
Dicot
Grass1
Grass2
Grass3
Grass4
**
**
*
*
*
n.s.
n.s.
*
Ib_1
Ib_2
VIc
95% confidence interval dN / dS ratio


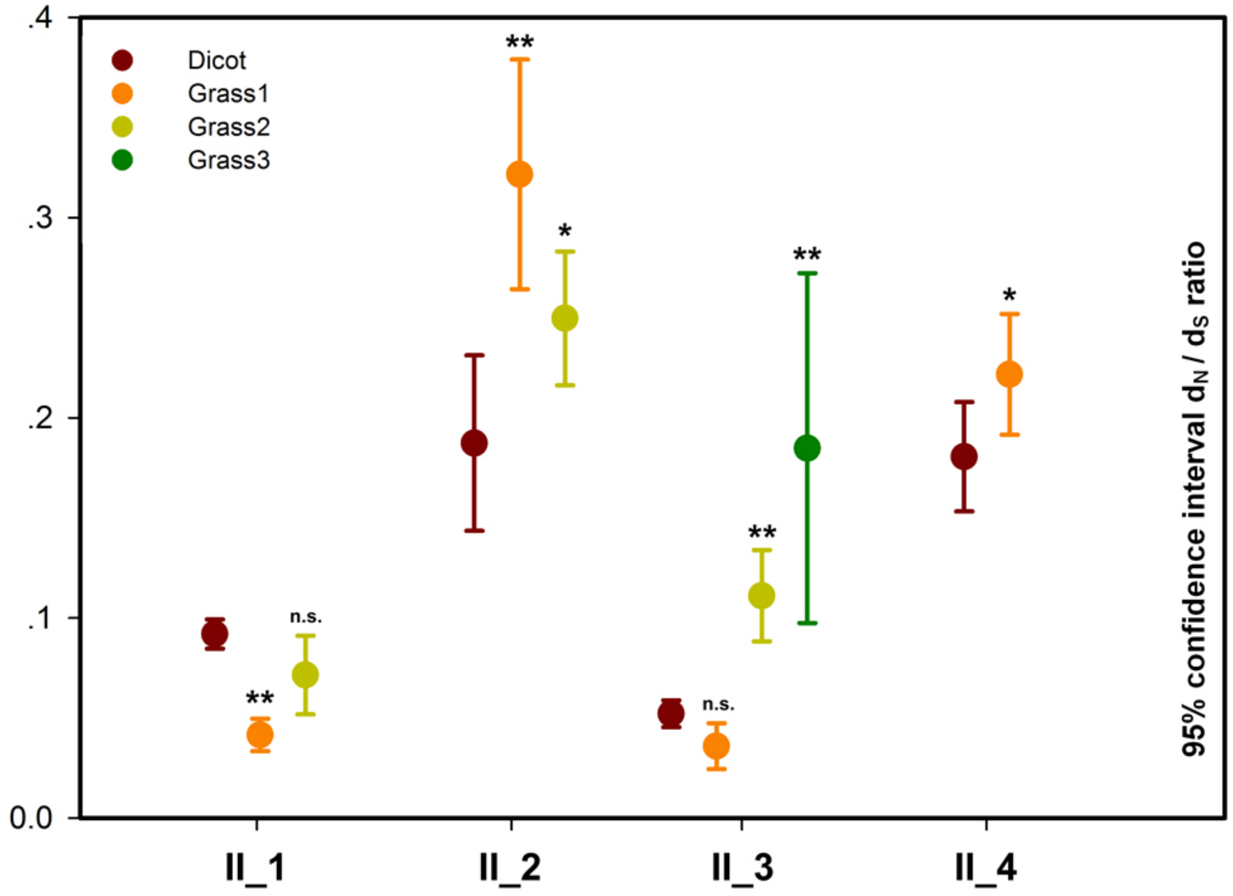

.4
.3
.2
.1
0.0
Dicot
Grass1
Grass2
Grass3
**
*
**
*
**
**
n.s.
n.s.
II_1
II_2
II_3
II_4
95% confidence interval dN / dS ratio

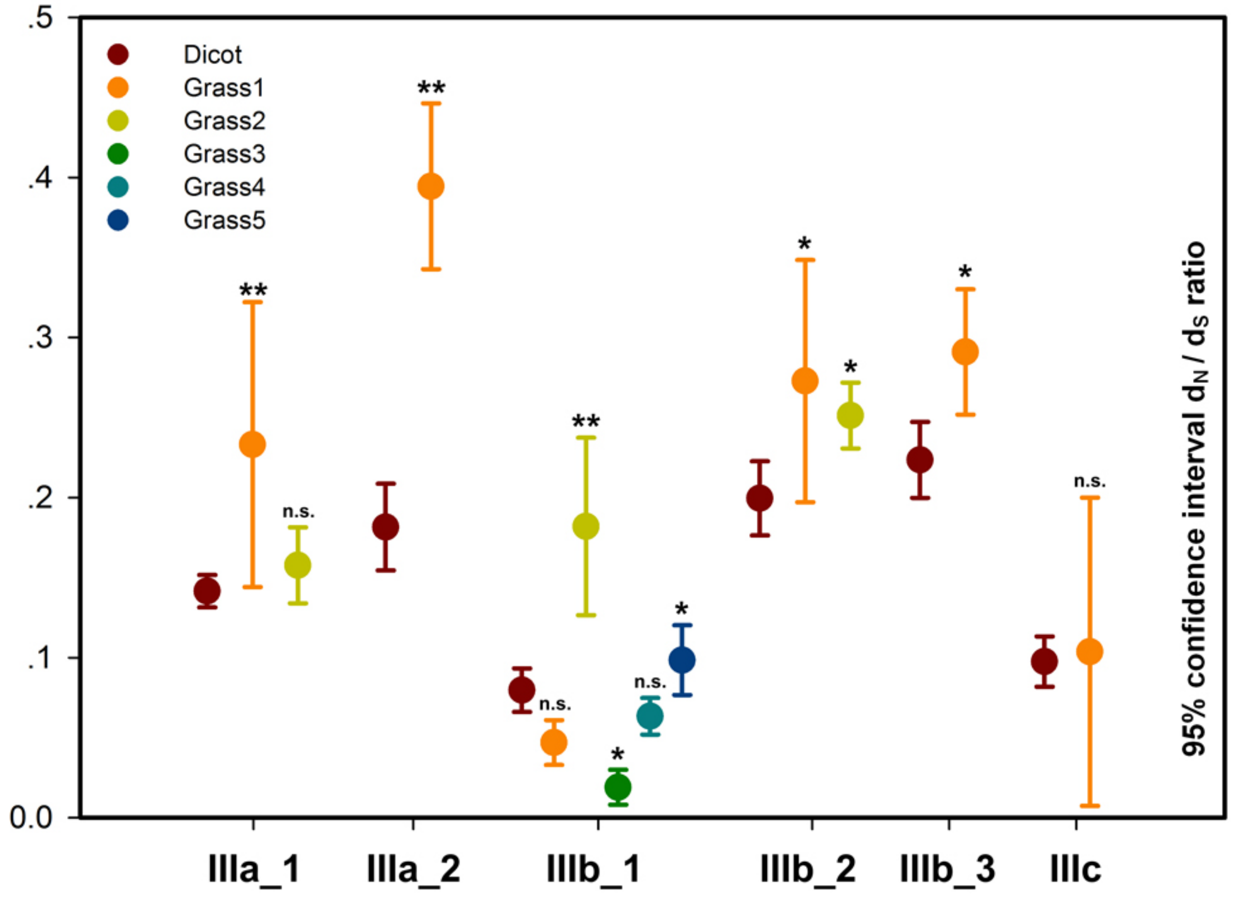
Dicot
Grass1
Grass2
Grass3
Grass4
Grass5
.5
.4
.3
.2
.1
0.0
**
**
*
*
*
n.s.
**
n.s.
*
n.s.
*
n.s.
IIIa_1
IIIa_2
IIIb_1
IIIb_2
IIIb_3
IIIc
95% confidence interval dN / dS ratio

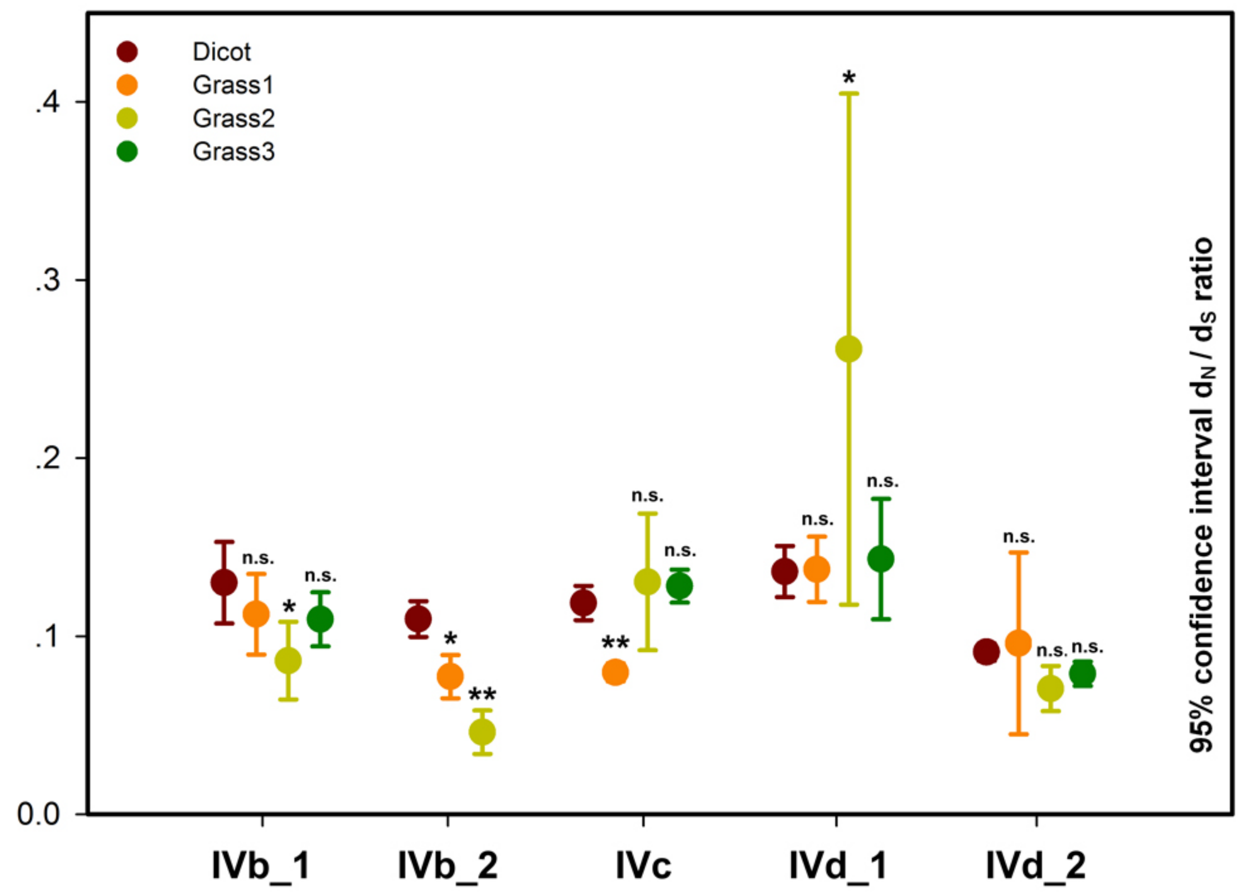
Dicot
Grass1
Grass2
Grass3
.4
.3
.2
.1
0.0
*
n.s.
n.s.
n.s.
n.s.
n.s.
n.s.
*
n.s.
*
**
**
n.s.
n.s.
n.s.
IVb_1
IVb_2
IVc
IVd_1
IVd_2
95% confidence interval dN / dS ratio

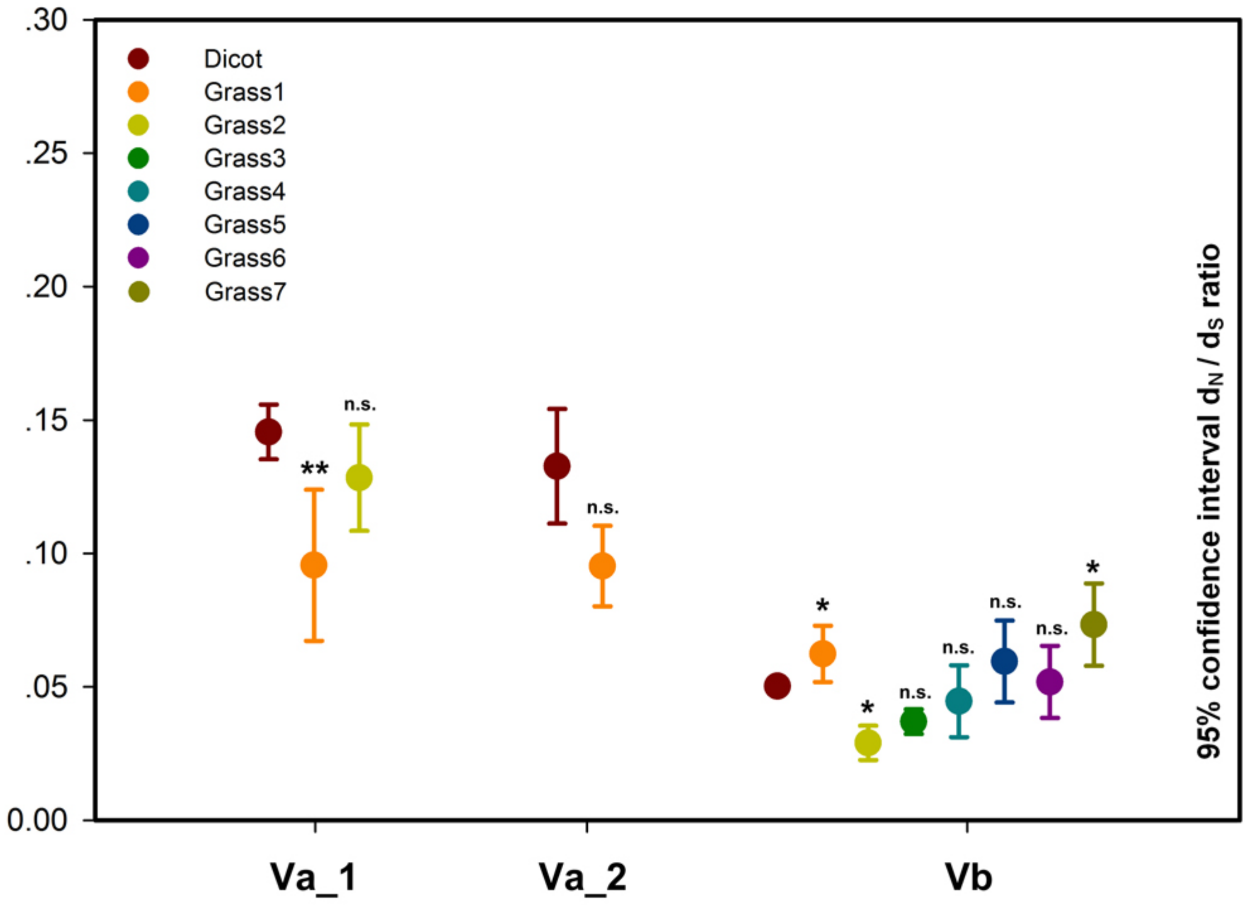


**Figure S3.** Pairwise estimation of $\omega$ values in each dicot or grass subgroup within subfamilies considered in this study. In each of the subfamilies, a significance test on the difference between dicot subgroup and grass subgroup was carried out, and its result, listed in Table S1, is indicated by n.s. (no significance), * ($P < 0.05$), and ** ($P < 0.01$).
